# The effectiveness and safety of lifestyle medicine and integrative therapies in inflammatory arthritis: an umbrella review using a hierarchical evidence gathering approach

**DOI:** 10.3389/fmed.2024.1357914

**Published:** 2024-03-13

**Authors:** Joshua Lin, Jing Liu, Allana O’Fee, Chhiti Pandey, Sarah Benna-Doyle, Alison Maunder, Vibhuti Rao, Simon Alesi, Beverly Ng, Carolyn Ee

**Affiliations:** ^1^School of Medicine, Western Sydney University, Sydney, NSW, Australia; ^2^NICM Health Research Institute, Western Sydney University, Sydney, NSW, Australia; ^3^Monash Centre for Health Research and Implementation, Monash University, Melbourne, VIC, Australia; ^4^Department of Rheumatology, Westmead Hospital, Sydney, NSW, Australia

**Keywords:** rheumatoid arthritis, ankylosing spondylitis, gout, complementary therapies, exercise, diet

## Abstract

**Objective:**

An umbrella review was conducted to provide a comprehensive evaluation of the evidence on lifestyle medicine and integrative therapies for inflammatory arthritis.

**Methods:**

Five electronic databases were searched for umbrella reviews, meta-analyses, and systematic reviews of randomised controlled trials on acupuncture, diet, exercise, herbal medicine, nutrient supplements, and mind–body therapies for rheumatoid arthritis, spondyloarthritis, and gout published from January 2012 to December 2022. The primary outcomes were functional status and quality of life. Quality assessment was performed using the A MeaSurement Tool to Assess systematic Reviews (AMSTAR-2) tool, and the certainty of evidence for our primary outcomes was assessed using the Grading of Recommendations Assessment, Development, and Evaluation (GRADE) approach where possible.

**Results:**

We included 52 reviews. Exercise was beneficial for functional status in both rheumatoid arthritis and spondyloarthritis, with moderate certainty of evidence. Chinese herbal medicine in combination with disease-modifying anti-rheumatic drugs may improve functional status in rheumatoid arthritis (very low certainty evidence). Acupuncture may improve functional status in rheumatoid arthritis and pain in both rheumatoid arthritis and gout; however, the evidence is of very low certainty. Evidence for other therapies was not clinically significant; however, it suggests possible benefits from quercetin and polyunsaturated fatty acids. Yoga may result in a moderate improvement in functional status when used as an adjunct to medication; however, the certainty of evidence is very low. Diet interventions offered inconsistent improvements to functional status in rheumatoid arthritis, spondyloarthritis, and gout with low to very low certainty.

**Conclusion:**

Exercise should be prescribed for people with rheumatoid arthritis and spondyloarthritis. More research is needed to confirm or refute evidence for Chinese herbal medicine, acupuncture, yoga, and anti-inflammatory diets.

## 1 Introduction

Arthritis is a broad term for a group of diseases that cause pain and swelling in joints and connective tissues and encompass approximately 100 conditions (1). Arthritis is estimated to affect 53.2 million adults in the United States, with one in five people living with a diagnosis of arthritis (1). The most prevalent forms of arthritis include osteoarthritis, characterised by cartilage deterioration with a loss of joint space, and inflammatory arthritides (IA) such as rheumatoid arthritis (RA), spondyloarthritis, and gout (2). Although the pathogenesis of this latter group is diverse, IA have a chronic and progressive natural history that, if uncontrolled, may lead to irreversible joint damage (3).

The potential impact of IA on the quality of life is significant. People with RA and ankylosing spondylitis are more likely to experience persistent ongoing symptoms such as chronic pain and fatigue, as well as functional disability from progressive joint damage, resulting in substantial deficits to both physical and mental health when compared with the general population (4). Permanent work disability occurs in over a third of people (37%) with RA, and this impact is evident globally (5). The onset of disability may be rapid, with up to 20–30% of work disability occurring within the first 5 years of symptom onset (6).

The aim of treatment in IA is to relieve pain and stiffness and target the underlying disease process to restore function and prevent progressive joint damage (7). Pharmacological management is a mainstay of treatment with the use of disease-modifying anti-rheumatic drugs (DMARDs) to control disease activity. In RA and spondyloarthritis, this finding may involve conventional synthetic DMARDs such as methotrexate, as well as more novel biological and targeted synthetic drugs (8, 9). In chronic gout, pharmacotherapy with the use of urate-lowering therapy is strongly recommended for those with frequent flares, subcutaneous tophi, or radiographic evidence of joint damage (10).

There is currently an increased inclusion of lifestyle interventions such as dietary changes and exercise in clinical guidelines. Exercise is recognised as an essential part of disease management in spondylarthritis (8) and is strongly recommended in RA (9), although there is less certainty for other lifestyle recommendations in RA and gout (9, 10).

However, many people with IA also report using complementary and integrative therapies. While an estimated 47% of people with RA use complementary therapies worldwide, only 30% of patients report this use to their physician (11). For people with ankylosing spondylitis, the prevalence of integrative therapy usage is reported to be over 40% (12). The prevalence of integrative therapy usage in gout may be less than that in other rheumatic diseases at 23.9%, although the available research is limited (13).

Complementary medicine describes healthcare approaches not traditionally considered part of conventional medical care or originating outside of usual Western practice (14). Complementary medicine refers to a diverse range of practices and therapies, including acupuncture, mind–body therapies, and herbal medicines (14, 15). Integrative medicine describes the coordinated and multimodal use of conventional health approaches and complementary therapies to promote the overall wellbeing. This review will use the term “integrative therapies” to refer to these non-mainstream approaches (16).

The prevalence of integrative therapy utilisation may reflect patient-reported priorities for care in arthritis, which include the control of physical symptoms and the achievement of normalcy, self-efficacy, and general wellbeing (17). In Australia, users of integrative medicine tend to be well engaged with conventional health services and highly educated, but with multiple or chronic diseases causing a lower than average quality of life (15, 18). A scoping review elucidated the use of natural products in individuals with RA and reported that decreased pain intensity, improvement of sleep, alleviation of symptoms, health promotion, reduced swelling, reduced fatigue, and improved activity level were some of the criteria used by individuals with RA to assess whether a natural product was effective and were reasons for the use of integrative therapies (11). Integrative therapies may offer patients additional means of achieving their desired health outcomes.

However, common concerns about integrative therapy use reported by medical practitioners include: a lack of comfort in answering questions about integrative therapies, and a lack of high-quality experimental evidence regarding their efficacy and safety (14).

The available high-level evidence for integrative therapies in the specific population of IA is limited. To the best of our knowledge, an umbrella review focussing on integrative therapies for chronic IA has not yet been conducted. Clinical guidelines from national rheumatology associations are limited. The American College of Rheumatology (ACR) recently published a guideline on exercise, diet, and integrative therapies in RA, the first to our knowledge; however, the scope is limited to RA alone and does not include herbal therapies (19).

Given the prevalence of integrative therapy utilisation and the scarcity of high-level evidence in the population of IA, additional research is necessary to facilitate an informed discussion between patients and practitioners. The aim of this umbrella review is to identify and synthesise existing evidence from umbrella reviews, meta-analyses and systematic reviews of randomised control trials (RCTs) in order to systematically evaluate the effectiveness and safety of lifestyle medicine and integrative therapies in the management of chronic IA.

## 2 Methods

An umbrella review, also known as an overview of reviews, is a systematic approach for the identification of multiple systematic reviews on a related topic for the purpose of collating results for pre-identified outcomes. It may be used to describe the current body of systematic review evidence or adapt existing evidence towards a new clinical question (20).

### 2.1 Protocol registration

Our study was designed and reported in accordance with the PRIOR reporting guidelines (21). A protocol was developed a priori and registered on Open Science Framework (https://osf.io/5y39u/) on 7th February 2024.

### 2.2 Search strategy

A comprehensive search strategy was developed by a team of clinician-researchers, including a rheumatologist and general practitioner, with assistance from a university librarian. One author (JL) conducted a systematic search of five electronic databases (Ovid MEDLINE, PsycINFO, Embase, CINAHL, and Cochrane) up to 16 September 2022. The search was updated on 31 December 2022. A full copy of the search strategy is available in the Supplementary material.

Two of the four authors (JL, CP, CE, and SD) independently assessed the title and abstracts of the identified studies using our selection criteria below. Two of the four authors (JL, SD, CP, and CE) then independently assessed full-text articles. Any disagreements were resolved either by consensus or by discussion with a third author.

### 2.3 Selection criteria

The inclusion criteria for this study were designed using the Population, Intervention, Comparator, Outcome, Study Design (PICOS) model and are summarised in Table 1.

**Table 1:** Inclusion criteria—eligibility criteria.

Population	All adults (≥18) with a diagnosis according to established clinical criteria of chronic inflammatory arthritis, such as rheumatoid arthritis, ankylosing spondylitis, psoriatic arthritis, and gout. The following were excluded: Studies enrolling participants <18 years (where age limitations were not specified, the included studies were evaluated to see if age characteristics were extracted and reviews that did not report age characteristics or did not provide separate data analysis for paediatric and adult populations) were excluded. Studies that evaluated acute arthritis (e.g., septic arthritis, acute gout flare treatment) or mechanical disease (e.g., osteoarthritis) were excluded. Studies on participants without joint involvement as the primary manifestation of disease, such as those that enrolled patients with psoriasis or cutaneous lupus without specifying arthritis, were excluded.
Interventions	Adjunctive or stand-alone treatment with any type of acupuncture, Chinese herbal medicine, mind–body medicines, nutraceuticals, dietary interventions, massage and/or exercise therapy, or a combination of these with any duration and frequency.
Comparisons	Control can be any comparative control.
Outcomes	Primary outcomes: Functional status and health-related or general quality of life (including instruments like health assessment questionnaire/HAQ and variants, Patient-Reported Outcome Measurement Information System/PROMIS, medical outcomes study/MOS item short score). Secondary outcomes: Pain (e.g., numerical rating, visual analogue, joint pain index, Lysholm scores, and Western Ontario and McMaster Universities Arthritis Index). Clinical assessments of disease activity or clinical response (e.g., disease activity score-28/DAS28, tender joint count/TJC, swollen joint count/SJC, duration of morning stiffness/DMS, and ACR20), inflammatory biomarkers (e.g., C-reactive protein/CRP, erythrocyte sedimentation rate/ESR, and serum uric acid/sUA), and symptom control. Safety and tolerability data including side effects, adverse reactions, adverse events, and attrition. Any studies whose primary outcomes are listed above were included. Studies whose primary outcomes were not listed above (e.g., depressive or anxious symptoms, fatigue) were excluded.
Study design	All umbrella reviews, meta-analyses, and systematic reviews of data from randomised controlled trials (RCTs) meet the following criteria: Published within the last 10 years. Clear inclusion criteria. Describes a systematic search and data extraction procedure. Reports relevant quality assessment of included studies, e.g., Risk of Bias for randomised controlled trials in a systematic review/meta-analysis, and A MeaSurement Tool to Assess systematic Reviews (AMSTAR)/Risk of Bias Assessment Tool for Systematic Reviews (ROBIS) or similar validated instrument for systematic reviews in an umbrella review. English language. Reviews that included non-randomised trial designs were included only if a separate analysis of data from randomised trials was available. Published conference abstracts and clinical guidelines were included if they included an umbrella or systematic review that fulfilled the above criteria.

### 2.4 Data collection and analysis

#### 2.4.1 Hierarchal evidence gathering

Due to the broad scope of the research question and resource limitations, it was not feasible to gather data from all studies. We have previously developed a systematic approach towards data gathering, favouring the top tiers of evidence (22). One author (JL) reviewed the extracted data to identify the most recent and highest tier of evidence available using the following hierarchy in order: (1) umbrella reviews; (2) network meta-analyses; (3) meta-analysis of double-blind randomised control trials (RCTs); (4) meta-analysis of RCTs; and (5) systematic reviews. The selection was verified by a second author (CE).

Where there were multiple high-tier reviews available using the same PICO criteria, the study with the most recent search date was chosen. Where an older umbrella review or network meta-analysis covered the same topic as newer meta-analyses, the latest meta-analysis was used to update the results of the prior review. If there was doubt regarding the overlap of evidence, underlying references were compared, and a citation matrix was produced. If the overlap was high or greater (>15%) as described in Pieper 2014, the review with the most recent search end date was retained (23, 24). Where there was a partial overlap between included studies, only the most recent data for a specific intervention-control comparison were extracted.In this way we aimed to avoid overlap and present the most recent data from the highest tier of evidence for each individual comparison of intervention versus comparator in specific patient groups and for specific outcomes.

#### 2.4.2 Data extraction

One review author (JL) extracted relevant information from the included studies, including study design, population, intervention and comparator details, and outcome information. Ten percent of the included studies were extracted in duplicate and verified by a second author (CE) to ensure consistency. Outcomes were extracted as weighted or standardised mean differences with confidence intervals for continuous outcomes, risk ratios or odds ratios for dichotomous outcomes, and the Grading of Recommendations Assessment, Development, and Evaluation (GRADE) (25) quality rating was extracted for primary outcomes where available.

#### 2.4.3 Quality assessment of included reviews

Included studies were assessed using the A MeaSurement Tool to Assess systematic Reviews (AMSTAR-2) tool (26). This was done in duplicate and independently in pairs by JL, CP, CE, VR, AM, JLiu, and AO, with disagreements resolved by discussion and a third reviewer (CE, AO, and JL) arbitrating if necessary. An overall rating of study quality from “critically low” to “high” was given based on the number of critical and non-critical domains met by the included reviews, as described in Shea et al. (26). To the best of our knowledge, there is no validated tool to assess the methodological quality of umbrella reviews, and these were therefore not assessed for quality.

#### 2.4.4 Certainty of evidence

The GRADE approach (25) was used to assess and report the certainty of evidence. Due to resource limitations, only functional status was assessed. Where functional status was not available, a GRADE assessment was conducted for composite measures of disease activity or clinical response. If the study authors conducted a GRADE assessment for these outcomes, then functional status was extracted directly.

Otherwise, GRADE assessments were conducted independently in duplicate pairs (JLiu, CE, AO, CP, and SA). Disagreements were resolved by discussion with a third reviewer (CE and JLiu), arbitrating if necessary. Not all studies reported enough information to conduct a GRADE assessment or reported a relevant outcome.

## 3 Results

### 3.1 Search results

**Figure 1:**
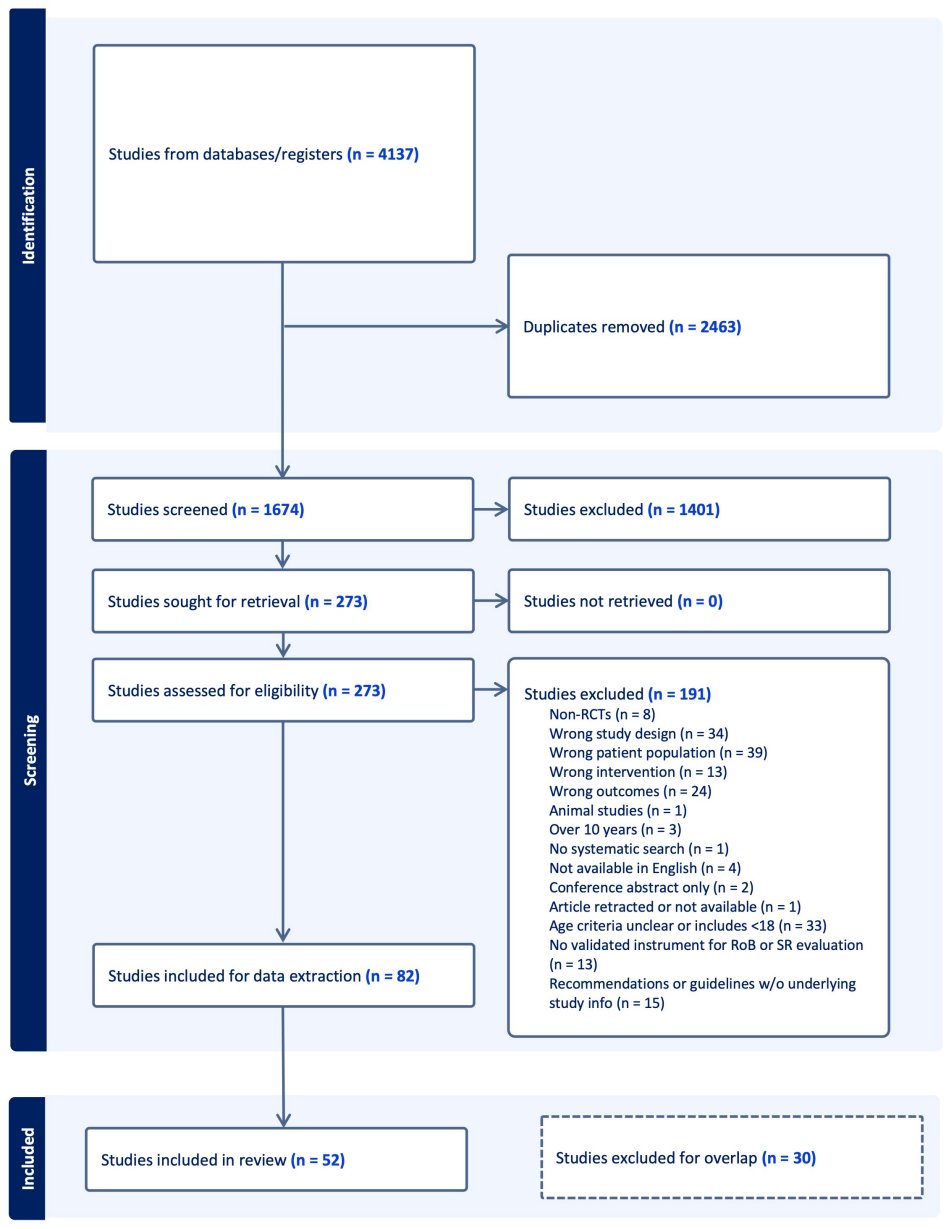
PRISMA flow diagram.

Figure 1 presents the search results, study selection, and inclusion process in a Preferred Reporting Items for Systematic Reviews and Meta-Analyses (PRISMA) flow diagram. After duplicates were removed, we screened 1,674 studies and excluded 1,401 at the title and abstract stage. A total of 82 studies were eligible to be included. Of the 82, 30 studies were subsequently excluded after the hierarchical evidence synthesis screening, leaving 52 studies in our review. A full list of the excluded studies with reasons is provided in Supplementary Table S1.

### 3.2 Study characteristics

Of the 52 included reviews, 38 enrolled participants had rheumatoid arthritis, 12 had spondyloarthritis, and 6 had gout. These 52 reviews include three (27–29) which included both RA and spondyloarthritis patient cohorts and one (30) which included RA and gout patient cohorts. Overall, 5 umbrella reviews, 3 network meta-analyses, 39 meta-analyses, and 5 systematic reviews were included. A broad overview of study information is presented below, with full characteristics of the included studies in Supplementary Table S2.

We found five studies on acupuncture and moxibustion, including one network meta-analysis (31) and four meta-analyses (32–35), covering several acupuncture and related therapies both alone and together with pharmacotherapy. There were three studies on diet interventions, including two meta-analyses (10, 36) and one systematic review (10, 37), encompassing Mediterranean, low-inflammatory, hypocaloric, and a range of other diets, most commonly compared with the usual diet. There were 14 studies evaluating exercise interventions, including three umbrella reviews, (38–40) nine meta-analyses, (27, 41–48), and two systematic reviews (49, 50) covering a range of exercise subtypes and programmes compared with both alternative treatments (active controls) and no physical activity (inactive controls).

There were 17 studies on Chinese herbal medicines, both as monotherapy and combination therapy with conventional pharmacotherapy, including 1 umbrella review, (51) 2 network meta-analyses, (52, 53), and 14 meta-analyses, (54–67). *Tripterygium wilfordii* Hook F extract was the intervention in seven studies, with nine other studies reviewing the glucosides of paeony, specific Chinese herbal decoctions and pills, and pooled analyses of Chinese herbal medicines. Non-Chinese herbal medicines were reviewed in five studies: one umbrella review (28), two meta-analyses, (10, 30) and two systematic reviews (10, 68, 69) covering spices and plant extracts primarily against placebo.

We found nine studies on nutrient supplements, including one umbrella review, (28) seven meta-analyses, (10, 29, 70–74), and one systematic review (10, 37). These evaluated omega fatty acids/fish oils, vitamins, probiotics, and other supplements against a variety of comparators, including placebo, medication, and other nutrient supplements. Mind–body therapies, including yoga, Tai Chi, and mindfulness programmes, were reviewed in three studies, all of which are meta-analyses (75–77).

### 3.3 Quality assessment

The methodological quality of the included reviews was mostly critically low or low (Table 2). Common critical flaws included not providing a list of excluded studies with reasons (item 7) or evidence of a protocol (item 2). A complete list of AMSTAR-2 findings is provided in Supplementary Table S3.

**Table 2:** AMSTAR-2 assessment of included studies*

Author	Critical flaws	Outcome	Author	Critical flaws	Outcome
Bjork (27)	—	Moderate	Ortolan (50)	7, 15	Critically low
Byrnes (49)	2, 7	Critically low	Pecourneau (43)	2, 7, 13, 15	Critically low
Daily (54)	7	Low	Philippou (69)	7, 15	Critically low
Feng (55)	7	Low	Regnaux (44)	15	Low
Fitzgerald (10)	2, 7	Critically low	Schonenberger (36)	7	Low
Geng (56)	—	Moderate	Sieczkowska (45)	7, 11, 15	Critically low
Gkiouras (70)	7	Low	Sigaux (74)	7	Low
Guan (71)	2, 7	Critically low	Sobue (46)	2, 7, 11, 13	Critically low
Han (57)	2, 7	Critically low	Sun (35)	2, 7, 11	Critically low
Jo (59)	7	Low	Wan (31)	7, 13	Critically low
Kou (72)	2, 7, 13, 15	Critically low	Wang (53)	7, 13	Critically low
Letarouilly (68)	2, 7	Critically low	Wang (63)	2, 7, 15	Critically low
Li (58)	7,	Low	Wang (62)	7, 13	Critically low
Li (52)	7, 13	Critically low	Williams (47)	2	Low
Li (32)	2, 7, 15	Critically low	Xu (64)	7	Low
Liang (42)	2, 7, 11	Critically low	Ye (76)	2	Low
Liang (41)	9, 15	Critically low	Ye (48)	7, 15	Critically low
Liu (60)	2, 7, 13, 15	Critically low	Zeng (29)	2, 15	Critically low
Luo (61)	7	Low	Zeng (30)	15	Low
Lu (34)	2, 7, 13	Critically low	Zhang (65)	2, 7	Critically low
Lu (33)	7, 11, 13	Critically low	Zheng (66)	2, 7, 13	Critically low
Mudano (75)	11	Low	Zhou (67)	2, 7, 13, 15	Critically low
Nguyen (73)	2, 7, 9, 13, 15	Critically low	Zhou (77)	2, 13, 15	Critically low
Ortolan (37)	2, 7, 15	Critically low			

*Umbrella reviews not assessed.

### 3.4 Findings: integrative therapies for rheumatoid arthritis

We included 38 reviews on integrative therapies for rheumatoid arthritis, covering acupuncture, diet interventions, exercise, Chinese and other herbal medicines, nutrient supplements, and mind–body interventions. A full summary of the findings is provided in Supplementary Table S4.

#### 3.4.1 Acupuncture and related techniques for rheumatoid arthritis

We included four reviews on acupuncture and moxibustion (one NMA and three MAs) (31–33, 35). Needle or laser acupuncture resulted in a clinically non significant improvement in functional status compared to sham or oral medication (32) but there was no additional improvement when combined with Western medicine (33). There may be no clinical improvement in disease activity with moxibustion in combination with a DMARD compared with DMARD alone but the evidence is very uncertain. Electroacupuncture was the only other subtype of acupuncture that improved disease activity in combination with a DMARD; however, these findings were based on a single trial (31).

Laser or needle acupuncture improved pain, quality of life, and biomarkers against a pooled control of sham or oral medications (32). When combined with Western medicine, needle acupuncture improved biomarkers, joint counts, and pain, and a number of other acupuncture subtypes improved biomarkers (33). Moxibustion had a mixed clinical response, improving ACR50 (American College of Rheumatology response criteria) but not ACR70 (35).

These reviews reported a limited number of adverse effects (AE). Minor skin irritation and swelling were the most common AE in the acupuncture group, as were gastrointestinal disturbances in the DMARD group.

#### 3.4.2 Diet interventions for rheumatoid arthritis

One MA reported on diet interventions. Pooled analysis with data from various anti-inflammatory diets (Mediterranean, vegetarian, vegan and ketogenic diets) suggest there may be improved functional status in comparison to an omnivorous diet which approached clinical significance (36), however the evidence is very uncertain. Anti-inflammatory diets also improved pain and SJC in the same pooled analysis; however, no improvements were reported for TJC, ESR, or CRP (36). Detailed information on AEs was not reported.

#### 3.4.3 Exercise for rheumatoid arthritis

There were six reviews on exercise interventions (one umbrella review and five meta-analyses). An umbrella review reported that a pooled analysis of exercise types, including aerobic, strength, aquatic, and combined aerobic/strength exercise, improved functional status, but either strength training or aquatic exercise independently did not (40). A meta-analysis conducted in 2022 reported that aerobic exercise may result in a clinically significant improvement in functional status compared to control, but the evidence is very uncertain (48). Another meta-analysis reported that physical activity and exercise were more effective for activity performance than inactive controls (no treatment, usual care) but not more effective than active controls involving alternative physical activity or treatment (27).

Overall, both aerobic and a pooled exercise group were effective for reducing pain; however, an improvement in biomarkers was only found for strength training (40, 48). Exercise did not demonstrate efficacy for modifying disease activity, irrespective of intervention type (40, 45, 46). Two reviews reported on safety, with no adverse events occurring in the intervention groups (47, 48).

#### 3.4.4 Chinese herbal medicine for rheumatoid arthritis

There were 14 studies on Chinese herbal medicines, including two reviews on various Chinese herbal medicines as a pooled group (57, 59), seven on *Tripterygium willfordii* Hook F extract (TwHF^2^) (also known as *leigongteng* or thundergod vine) (51, 53, 56, 60, 63, 64, 66), and five on other specific medicines (52, 54, 55, 61, 62).

Chinese herbal medicines combined with DMARDs may improve functional status compared with DMARDs alone but the evidence is very uncertain (57). However, as monotherapy, they may not improve functional status compared to conventional pharmacotherapy (59). Chinese herbal medications, either as monotherapy or in combination with DMARDs, improved pain, joint counts, and biomarkers compared to pharmacotherapy alone (57, 59). Both comparisons were also associated with a reduced incidence of AEs.

Monotherapy with TwHF extract showed an improved clinical response (ACR20) when compared with most DMARDs except cyclosporine A (53). Additionally, a combination of TwHF with methotrexate also showed improved disease activity when compared with placebo or other medications (56). With regard to other outcomes, TwHF, either as a monotherapy or a combination therapy with various DMARDs, demonstrated benefit for biomarkers, disease activity, and pain across most comparisons, although the impact on joint counts was mixed (51, 56, 60, 63, 66). When compared with DMARDs, monotherapy with TwHF resulted in fewer AEs (63). However, no significant difference in AEs was found when TwHF monotherapy was compared with placebo or when combined with DMARDs (63, 64, 66).

A network meta-analysis compared four different compounds (*Bai Ju GuiZhi Decoction*/BHGZD, *Dang Gui Nian Tong Decoction*/DGNTD, *Si-Miao Pill*/SMP, and *Xuan Bi Decoction*/XBD) in combination with DMARDs against DMARDs alone and found that all four formulations reduced biomarkers. However, only XBD was superior to DMARDs for disease activity, although this was based on a single trial, and only BHGZD improved clinical response (ACR20). The highest reporting of AEs was in the groups receiving DMARDs, while the lowest was in the groups receiving SMP and DGNTD (52).

The combination of modified *Si-Miao Pill* and Western medicine reduced disease activity and biomarkers with fewer AEs in comparison with conventional pharmacotherapy (62). *GuiZhi-ShaoYao-ZhiMu Decoction*/GSZD, either as monotherapy or a combination therapy, resulted in improved joint counts and ESR as compared to conventional medication (54). Furthermore, a combination of GSZD and methotrexate also showed improved joint counts and biomarkers with fewer AEs when compared with placebo or other medications (55). Glucosides of peony in combination with a DMARD were reported by the review authors to have improved functional status based on one trial however we note a discrepancy where HAQ score was higher in the intervention group suggesting deterioration of functional status. Glucosides of paeony improved clinical response (ACR20/50/70), disease activity, and pain with a reduced incidence of AEs in the intervention group; however, we also note the discrepancy with pain outcomes, where pain scores were reported to be higher in the intervention group, but review authors noted this as an improvement (61).

#### 3.4.5 Other herbal medicines for rheumatoid arthritis

We found four studies reviewing other non-Chinese herbal medicines (one umbrella review (28), one meta-analysis (30), and two systematic reviews (68, 69)). A systematic review reported that several spices, including garlic, ginger, cinnamon, and saffron, demonstrated mixed improvements in disease activity, pain, and biomarkers; however, these improvements were from single, small trials, albeit all double-blind placebo-controlled, and assessed as a low risk of bias (68). A meta-analysis reported that curcumin may result in clinically non-significant improvement in disease activity, biomarkers and joint counts, but the evidence is very uncertain. There was no difference between groups for AEs (30). One systematic review reported that evening primrose oil decreased disease activity and pain; however, this evidence is observed from two single trials (69).

An umbrella review reported no benefit in functional status, pain, disease activity, or quality of life with pomegranate, *aloe vera*, or rose hip powder. A single RCT reported improvements in disease activity, joint count, and ESR with a combined intervention of ginger, curcumin, and black pepper (28). The overall certainty of evidence for other herbal medicines for RA ranged from very low to low, with many outcomes only supported by single, small RCTs.

#### 3.4.6 Nutrient supplements for rheumatoid arthritis

Nutrient supplements were reported by seven studies (one umbrella review (28) and six meta-analyses (29, 70–74)). Probiotics did not result in any change in disease activity, joint counts, or ESR, and no serious AEs were reported (29). Vitamin K and folic acid compared to placebo were of no benefit for functional status or disease activity (73). Vitamin D improved disease activity but not pain, with mixed improvements in biomarkers and index counts (71). There was no difference between Vitamin E and pooled controls for pain (73), although a subsequent MA reported benefits for disease activity and joint counts, with a mixed impact on biomarkers (72).

In one MA, omega-3 fatty acids (FAs) had no benefit for disease activity, pain, TJC, SJC, or biomarkers (70). Conversely, another MA found omega-3 and omega-6 FAs may improve functional status, joint counts, pain, disease activity and ESR, but the evidence is very uncertain. Greater effects were observed in higher doses (>2 g/day) and after a 3 months duration (74).

An umbrella review on a heterogenous range of nutrient supplements found that quercetin may be beneficial for functional status though this is limited by very low certainty evidence, potassium improved disease activity, pain, joint counts and biomarkers, and one trial reported that mussel extracts improved disease activity (28). Antioxidants may improve functional status and pain, but not other outcomes. The additional interventions in the review showed no significant effects.

#### 3.4.7 Mind–body interventions for rheumatoid arthritis

We found three meta-analyses evaluating mind-body interventions (75–77). Yoga may improve functional status when used as an adjunct to conventional medication and when compared alone against any non-yoga intervention although not as stand-alone therapy against usual care (76). However these findings are limited by very uncertain evidence. Mindfulness-based interventions may result in a non-clinically significant reduction in disease activity in comparison with routine nursing but the evidence is very uncertain (77). Tai Chi, compared with no exercise or other types of exercise, did not benefit functional status (75).

With regard to pain, yoga resulted in a small reduction in disease activity, however, no significant differences (clinically or statistically) were found for the other mind–body therapies. One study reported withdrawals due to AEs and found no significant differences between groups (75).

### 3.5 Integrative therapies for spondyloarthritis

We included 12 reviews on exercise, diet interventions, and nutrients for spondyloarthritis (see Supplementary Table S5). We did not find any systematic review evidence on acupuncture or herbal medicines for spondyloarthritis.

#### 3.5.1 Exercise for spondyloarthritis

A total of 9 studies reported on exercise, including overall exercise and exercise subtypes against active and inactive controls (two umbrella reviews (38, 39), five meta-analyses (27, 41–44), and two systematic reviews (49, 50)). Overall, exercise resulted in clinically non-significant improvements in functional status measured using the Bath Ankylosing Spondylitis Functional Index/BASFI when compared to no intervention (44) and usual care (44) while there may also be clinically non-significant improvements compared to pooled controls but the evidence is very uncertain (43). There was no effect on activity performance when compared to active controls (e.g., other exercise programmes, home exercises, and medications) (27).

Home-based exercise may result in a clinically nonsignificant improvement in BASFI when compared with other exercise types and medical therapy but the evidence is very uncertain (42). There is high certainty of evidence for the benefit of combined aerobic and strength exercise compared to any control for functional status in axial spondyloarthritis and moderate certainty evidence for the benefit of strength exercise on functional status (38). However, no benefit was found for aerobic exercise alone or aquatic exercise (38), and there is very limited evidence of benefit for Pilates (49).

General exercise programmes and most exercise subtypes (home-based, land-based, aquatic, aerobic, aerobic/strength, strength) improved pain and disease activity when compared to no intervention, usual care, or a pooled control (27, 38, 39, 42–44, 50). However, exercise combined with anti-TNF agents did not improve disease activity compared with medication alone (41). Only one meta-analysis reported on AEs, finding a single AE in the intervention group with none in the control group (two trials, *n* = 110) (44).

#### 3.5.2 Diet for spondyloarthritis

One systematic review provided limited information on diet interventions. A hypocaloric diet showed improved disease activity in participants with psoriatic arthritis who had not improved on traditional DMARDs. The frequency of achieving minimal disease activity was correlated with increasing weight loss (37).

#### 3.5.3 Nutrient supplements for spondyloarthritis

Three studies were reported on nutrient supplements (one umbrella review (28), one meta-analysis (29), and one systematic review (37)). A combination of selenium, coenzyme Q10, and vitamin E may improve disease activity in psoriatic arthritis, but the evidence is very uncertain (28). Marine omega-3 fatty acids did not show benefit for functional status, pain, inflammatory markers, or TJC in psoriatic arthritis, while there is low certainty evidence for an improvement in SJC (28).

Alpha-linoleic acid, linoleic acid, and polyunsaturated fatty acid supplementation did not reduce disease activity in ankylosing spondylitis, but the evidence is very uncertain (28). Probiotics compared to a pooled control group in ankylosing spondylitis did not significantly affect functional status (BASFI) or disease activity (BASDAI, TJC, and SJC) or show any differences in AEs (29). High-dose fatty acids (4.55 g) were found to significantly improve disease activity (BASDAI) in ankylosing spondylitis compared to lower doses (1.95 g), although this was based on a single trial (37).

### 3.6 Integrative therapies for gout

We found six reviews on acupuncture, diet, Chinese herbal medicines, other herbal medicines, and nutrient supplements for gout (see Supplementary Table S6). We did not find any reviews on exercise or mind–body therapies.

#### 3.6.1 Acupuncture for gout

Acupuncture, including manual and electroacupuncture, may result in greater serum uric acid reduction as compared to Western medicine but the evidence is very uncertain (34). Additionally, clinically significant benefits were found for pain reduction and mixed effects for inflammatory biomarkers. Fewer AEs were reported in the intervention group.

#### 3.6.2 Diet for gout

There was very low certainty of evidence from a single trial reported in Fitzgerald 2020 that a 6 months purine-limited diet had no effect on serum uric acid or the rate of gout flares (10). The level of dairy protein intake showed no effect on sUA or gout flares (10).

#### 3.6.3 Chinese herbal medicines for gout

Three studies reviewed Chinese herbal medicines, both as a group and *Guizhi-Shaoyao-Zhimu Decoction*/GSZD individually. Chinese herbal medicines as a pooled analysis of either monotherapy or in combination with Western medicines may improve sUA and CRP, but not pain (58). There was very low certainty of evidence that Chinese herbal medicine decoctions may reduce serum uric acid compared to Western medicine in another meta-analysis. The relative risk of AE was reported to be lower in the intervention group (67).The duration of treatment/follow-up was not reported in either meta-analysis.

The GSZD herbal formula may improve sUA both alone and as combined therapy when compared to conventional pharmacotherapy, but the evidence is very uncertain. The GSZD formula, whether as monotherapy or a combination therapy, improved biomarkers (ESR, CRP) and reduced the risk of AE in the intervention group (65). Gout flares were not reported in these reviews.

#### 3.6.4 Other herbal medicines for gout

We found two meta-analyses (10, 30) on other herbal medicines. Based on a single trial, curcumin was not found to be effective in reducing sUA when compared with placebo (30). Similarly, cherry extract also showed no efficacy for reducing sUA or the incidence of gout flares based on one small trial (10).

#### 3.6.5 Nutrient supplements for gout

A meta-analysis conducted to inform the 2020 American College of Rheumatology Guideline for the management of gout reported that Vitamin C resulted in a clinically insignificant sUA reduction compared with starting or increasing allopurinol with no effect on gout flares (based on a single trial) (10).

## 4 Discussion

To the best of our knowledge, this is the first umbrella review to evaluate the evidence on a comprehensive range of lifestyle medicine interventions and integrative therapies for spondyloarthritis and gout. We provide additional evidence on herbal medicines for RA, complementing the 2022 ACR guidelines (19).

For RA, exercise interventions (particularly aerobic exercise) and Chinese herbal medicine in combination with DMARDs resulted in clinically significant improvements in functional status. The evidence evaluating anti-inflammatory diets (e.g., Mediterranean) approached clinical significance (36). There was very low certainty and conflicting evidence on the benefits of various acupuncture modalities, and the results were largely but not clinically significant, except for pain. Evidence for other therapies was very limited and mostly negative or not clinically significant, although the available evidence suggests possible benefits from quercetin and omega-3 and omega-6 fatty acids (28, 74). Yoga resulted in moderate improvements in functional status when used as an adjunct to medication; however, the certainty of evidence was very low (76). Our findings broadly align with recommendations from the ACR guidelines, which offer conditional support for acupuncture, mind–body therapies, and a Mediterranean diet. At present, the ACR conditionally recommends against all supplementation (19).

We found very low to moderate certainty evidence of the benefit of exercise in RA, particularly for functional status, with more limited improvement in inflammatory biomarkers or disease activity. This effect estimate was greater in combined aerobic and strength exercise, with weaker evidence for other subtypes (40, 48). There was moderate certainty that exercise was beneficial against inactive controls (no exercise/usual care) but not active controls (other exercise or treatment). This finding may suggest that the type of exercise is less important than maintaining regular exercise for the improvement of functional status (27). ACR offers a strong recommendation for consistent engagement in exercise to improve the functional status (19), which is consistent with the findings in our review.

Chinese herbal medicines, particularly TwHF, are widely used in China to treat RA (78). TwHF may have a clinically significant benefit for functional status when used as an adjunct to DMARDs, but the certainty of evidence is very low (57). Individual remedies that demonstrated benefit for clinical response (ACR criteria) and disease activity include TwHF extract and glucosides of paeony but with low to very low certainty. A network pharmacology analysis suggests that five main compounds are involved in the mechanism of action of Chinese herbal medicines: quercetin, stigmasterol, sitosterol, kaempferol, and beta-sitosterol. These compound act to mediate inflammatory markers and may have immunomodulatory effects (52). However, reports of hepatic and renal impairment with the ingestion of TwHF have led to warnings issued by the State Food and Drug Administration in China and by the Medicines and Healthcare Products Regulation Agency in the United Kingdom (79). TwHF may result in gastrointestinal, reproductive, dermatologic, haematologic, and cardiovascular AEs (80). A clinical practice guideline on the use of TwHF for RA recommends monitoring of toxicity during administration due to the risk of adverse effects (81). However, a network meta-analysis did not find evidence of an increase in AEs when TwHF monotherapy was compared to DMARDs (53). Evidence on the safety of other Chinese herbal medicines is somewhat limited, as 33% of RCTs in one meta-analysis did not report on AEs. The AEs reported were abnormal liver and renal function tests and gastrointestinal reactions (53). The lowest reporting of AEs was with *Si-Miao Pill* and *Dang Gui Nian Tong Decoction*, whereas the highest reporting was DMARDs alone or *Xuan Bi Decoction* + DMARDs (52). Consideration must also be given to challenges relevant to Chinese herbal medicine use, which include variable quality control and regulation of herbs and a lack of accreditation standards for Chinese medicine practitioners in most countries outside of China. Limited information on dosing regimens and pharmacological assessments of drug levels for herbal medicines add further challenges to the interpretation of the data (82).

In spondyloarthritis, exercise compared to both inactive and pooled controls (usual care, physical therapy, and education) showed very low to moderate certainty of evidence for benefit (43, 44). There was moderate to high certainty of evidence that combined aerobic and strength exercises, or strength exercises alone, have a moderate effect size (SMD ≥ 0.5) on functional status (38). Our study findings are consistent with the European Alliance of Associations for Rheumatology (EULAR) guidelines that encourage regular exercise and recommend combined aerobic and strength exercises to improve functional status (83). However, at present, the evidence is very uncertain about the benefits of other interventions such as diet and nutrient supplements.

Acupuncture may be of benefit for pain reduction in gout (34); however, we are uncertain about the benefits of other interventions, including herbs and nutrient supplements. While we report findings from three reviews (58, 65, 67) on Chinese herbal medicines, there were few clinical outcomes (such as flare prevention) reported; hence, we are uncertain about the benefits of Chinese herbal medicines for the management of gout.

Although most of our findings are very uncertain due to the limitations of the available evidence, many of our studied interventions are relatively low risk and may provide additional benefits to people with inflammatory arthritis. While reporting of AEs was somewhat limited, the available evidence on acupuncture, mind–body therapies, curcumin, and probiotics suggests that there were no serious AEs in the intervention groups, and in fact, reduced AEs in the intervention group were commonly reported. These interventions are often widely available and used, particularly in China, where traditional Chinese medicine (acupuncture and Chinese herbal medicine) is well integrated with Western or mainstream medicine and is used by three-quarters of the population nationwide for the management of multimorbidity and chronic disease (84). There is a need for improved reporting of adverse events from RCTs in order to inform shared decision-making incorporating scientific evidence, clinical expertise, and patient values and preferences.

The strength of our review is its focussed selection of the most recent, hierarchical, and highest-level evidence available. The broad scope of our research question allowed us to identify links between integrative therapies across different pathologies and add to existing guidelines. We used validated tools and processes, such as AMSTAR-2 and GRADE, to assess study quality and confidence in effect estimates. Given the relative lack of existing overviews of integrative therapies in IA, our review provides a more comprehensive summary through a hierarchical evidence-gathering approach.

However, this approach is not without limitation. Only more recent studies were included, which may have resulted in the exclusion of older, yet high-quality reviews. Despite our efforts to minimize overlap, it may be possible that some primary research has been duplicated among the included reviews. Our study selection was limited to reviews of RCTs, which avoids bias arising from non-randomised designs, although it may result in a less comprehensive overview. Despite this limitation, the purpose of our review was to focus on high-level evidence in order to translate this evidence into recommendations for clinical practice. The study design also does not allow for a direct comparison of different interventions. Furthermore, due to resource limitations, we performed a GRADE assessment for our primary outcomes of functional status and quality of life only and cannot comment on the certainty of evidence for other outcomes.

There is a need for further rigorous RCTs and primary research to increase confidence in effect estimates. Many findings were downgraded for a serious risk of bias in underlying studies, inconsistent findings, and small study sizes, resulting in consistently low certainty of evidence. In addition, utilisation of standardised outcome measures, preferentially the use of validated patient-reported outcome measures in addition to biomarkers, would increase reliability and allow for more robust evaluations of efficacy, particularly in herbal medicine interventions. At present, the available evidence evaluating integrative interventions for IA is the most comprehensive for RA. Little to no high-level evidence was found for acupuncture, diet, mind–body, and herbal interventions in spondyloarthritis. Similarly, evaluations of diet, exercise, and mind–body therapies in gout are sparse, indicating a considerable need for further research in these areas. Future reviews should ensure their methodology aligns with established reporting guidelines and, in particular, provide a list of excluded studies with a rationale and evidence of a protocol.

## 5 Conclusion

There is consistent, moderate-quality evidence that exercise is beneficial for functional status in RA and spondyloarthritis. The low to very low overall quality and certainty of evidence for most diet interventions, nutrient supplements, herbal medicines, mind-body therapies and acupuncture across the IA literature precludes our ability to determine benefit. There is a pressing need for further high-quality research on integrative therapies for IA, particularly in spondyloarthritis and gout, to improve the quality of life, reduce symptom burden, and prevent disability.

## Author contributions

JoL: Conceptualization, Data curation, Formal analysis, Methodology, Project administration, Writing – original draft, Writing – review & editing. JiL: Data curation, Formal analysis, Writing – original draft, Writing – review & editing. AO: Data curation, Formal analysis, Project administration, Writing – original draft, Writing – review & editing. CP: Data curation, Formal analysis, Writing – original draft, Writing – review & editing. SB-D: Data curation, Formal analysis, Writing – original draft, Writing – review & editing. AM: Data curation, Formal analysis, Writing – original draft, Writing – review & editing. VR: Data curation, Formal analysis, Writing – original draft, Writing – review & editing. SA: Data curation, Formal analysis, Writing – original draft, Writing – review & editing. BN: Conceptualization, Methodology, Writing – original draft, Writing – review & editing. CE: Conceptualization, Data curation, Formal analysis, Funding acquisition, Methodology, Project administration, Resources, Supervision, Validation, Writing – original draft, Writing – review & editing.

## Footnotes

^2^In traditional Chinese medicine, TwHF is an important extract widely used for various autoimmune-mediated inflammatory diseases, with increasing interest in its anti-inflammatory and immunosuppressive pharmacological characteristics.
