# Supplementary material for: The effectiveness and safety of lifestyle medicine and integrative therapies in inflammatory arthritis: an umbrella review using a hierarchical evidence gathering approach
Source: Front Med (Lausanne). 2024 Mar 13;11:1357914. doi: 10.3389/fmed.2024.1357914 (PMC10965540; doi:10.3389/fmed.2024.1357914)
Supplement: Supplementary file 1 [file Data_Sheet_1.docx]

Supplementary Material

# Supplementary Data

## Search History Details

### Search History for Ovid MEDLINE(R) and EMBASE

1 (Inflammatory arthritis or monoarthritis or oligoarthritis or polyarthritis).mp. 16616

2 exp Arthritis, Rheumatoid/ or Rheumatoid arthritis.mp. or RA.mp. [mp=title, book title, abstract, original title, name of substance word, subject heading word, floating sub-heading word, keyword heading word, organism supplementary concept word, protocol supplementary concept word, rare disease supplementary concept word, unique identifier, synonyms] 196168

3 exp Spondylitis, Ankylosing/ or Ankylosing Spondyl*.mp. or Bechterew* Disease.mp. or spondyloarth*.mp. [mp=title, book title, abstract, original title, name of substance word, subject heading word, floating sub-heading word, keyword heading word, organism supplementary concept word, protocol supplementary concept word, rare disease supplementary concept word, unique identifier, synonyms] 26102

4 (exp Arthritis, Psoriatic/ or PsA.mp. or Psoriatic arthritis.mp. or Arthritis psoriatica.mp. or exp Lupus erythematosus, systemic/ or SLE.mp. or systemic lupus erythematosus.mp. or lupus.mp.) and (exp Arthritis/ or Inflammatory * arthritis.mp. or spondyloarth*.mp. or monoarthritis.mp. or oligoarthritis.mp. or polyarthritis.mp.) 19422

5 exp Gout/ or hyperur?caemi*.mp. or gout*.mp. [mp=title, book title, abstract, original title, name of substance word, subject heading word, floating sub-heading word, keyword heading word, organism supplementary concept word, protocol supplementary concept word, rare disease supplementary concept word, unique identifier, synonyms] 22494

6 1 or 2 or 3 or 4 or 5 249277

7 exp Complementary Therapies/ or Complementary Therap*.mp. or Complementary Medic*.mp. or Alternative Therap*.mp. or Alternative Medic*.mp. [mp=title, book title, abstract, original title, name of substance word, subject heading word, floating sub-heading word, keyword heading word, organism supplementary concept word, protocol supplementary concept word, rare disease supplementary concept word, unique identifier, synonyms] 263630

8 exp Integrative Medicine/ or Integrative Medic*.mp. or Integrative Therap*.mp. [mp=title, book title, abstract, original title, name of substance word, subject heading word, floating sub-heading word, keyword heading word, organism supplementary concept word, protocol supplementary concept word, rare disease supplementary concept word, unique identifier, synonyms] 3939

9 exp Holistic Health/ or Holistic Medic*.mp. or Holistic Therap*.mp. [mp=title, book title, abstract, original title, name of substance word, subject heading word, floating sub-heading word, keyword heading word, organism supplementary concept word, protocol supplementary concept word, rare disease supplementary concept word, unique identifier, synonyms] 8287

10 exp Healthy Lifestyle/ or Lifestyle Medic*.mp. or Lifestyle Interven*.mp. or Lifestyle Medic*.mp. or Lifestyle Therap*.mp. [mp=title, book title, abstract, original title, name of substance word, subject heading word, floating sub-heading word, keyword heading word, organism supplementary concept word, protocol supplementary concept word, rare disease supplementary concept word, unique identifier, synonyms] 20646

11 7 or 8 or 9 or 10 285704

12 exp Acupuncture Therapy/ or exp Acupuncture Points/ or exp Acupuncture, Ear/ or acupuncture.mp. 35330

13 exp Needles/ or needl*.mp. 177527

14 electroacupuncture.mp. or exp Electroacupuncture/ 6513

15 acupoint*.mp. 6501

16 acupressure.mp. or exp Acupressure/ 1645

17 Zhen Jiu.mp. 23

18 Moxibustion.mp. or exp Moxibustion/ 3595

19 Plants, Medicinal/ or Moxa.mp. or Artemisia/ 63957

20 Mugwort.mp. or exp Artemisia/ 3303

21 (traditional Chinese medicine.mp. or exp Medicine, Chinese Traditional/ or Chinese.mp.) adj medicine.mp. [mp=title, book title, abstract, original title, name of substance word, subject heading word, floating sub-heading word, keyword heading word, organism supplementary concept word, protocol supplementary concept word, rare disease supplementary concept word, unique identifier, synonyms] 34832

22 Traditional East Asian medicine.mp. or exp Medicine, East Asian Traditional/ 26180

23 exp Meridians/ or Meridian*.mp. or Meridian therapy.mp. 13294

24 Oriental medicine.mp. 1157

25 12 or 13 or 14 or 15 or 16 or 17 or 18 or 19 or 20 or 21 or 22 or 23 or 24 319074

26 exp Herbal medicine/ 2449

27 exp Drugs, Chinese Herbal/ 50479

28 exp Traditional Chinese Medicine/ 22731

29 exp Herbal/ 0

30 exp Teas, Herbal/ 269

31 exp Plants, Medicinal/ 61808

32 (medicin$ adj5 (chines$ or oriental$ or Tibetan$)).tw. 49321

33 (herbal medicin$ or medicin$ herbal$).tw. 19299

34 Plant$ medicin$.tw. 342

35 Medicin$ plant$.tw. 23594

36 TCM.tw. 13832

37 CHM.tw. 1777

38 26 or 27 or 28 or 29 or 30 or 31 or 32 or 33 or 34 or 35 or 36 or 37 170412

39 exp tai chi/ or Tai chi.mp. or Tai ji.mp. 2358

40 exp yoga/ or yog*.mp. 13571

41 (Asana* or Pranayama or Dharana or Dhyana).mp. 580

42 exp Mind-body therapies/ or mind-body therapies.mp. 46392

43 exp Mindfulness/ or mindfulness*.mp. 11936

44 MB*.mp. 167396

45 exp Meditation/ or Meditat.mp. 3586

46 exp relaxation therapy/ 9959

47 39 or 40 or 41 or 42 or 43 or 44 or 45 or 46 231246

48 exp dietary supplements/ or diet* supplement*.mp. or nutrition* supplement*.mp. [mp=title, book title, abstract, original title, name of substance word, subject heading word, floating sub-heading word, keyword heading word, organism supplementary concept word, protocol supplementary concept word, rare disease supplementary concept word, unique identifier, synonyms] 121096

49 (vitamin* or mineral* or nutrient* or food supplement* or meal replacement* or nutritional supplement* or health supplement* or multivitamin*).mp. [mp=title, book title, abstract, original title, name of substance word, subject heading word, floating sub-heading word, keyword heading word, organism supplementary concept word, protocol supplementary concept word, rare disease supplementary concept word, unique identifier, synonyms] 657411

50 (omega 3 or fish oil* or alpha lipoic acid or alpha linolenic acid or alpha linoleic acid or eicosapentaenoic or docosahexaenoic or fatty acid* or amino acid* or taurine or s-adenosyl methionine or creatine or acetylcysteine or cysteine or probiotic* or tryptophan or tocopherol or alphatocopherol or carotene or retinol or thiamine or riboflavin or niacin or niacinamide or nicotinic acid or pantothenic or pyridox* or biotin or methylfolate or 5-MTH* or levomefolic acid or folate or folinic acid or folic acid or inositol or cyanocobalamin or methylcobalamin or cobalamin or ascorbic acid or cholecalciferol or iron or ferrous or tocopherols or trace element or calcium or phosphorus or magnesium or potassium or manganese or zinc or selenium or boron or chromium or lycopene or isoflav* or flavonoid* or bioflavonoid* or micronutrient or carnitine or curcumin or tumeric).mp. [mp=title, book title, abstract, original title, name of substance word, subject heading word, floating sub-heading word, keyword heading word, organism supplementary concept word, protocol supplementary concept word, rare disease supplementary concept word, unique identifier, synonyms] 3213379

51 48 or 49 or 50 3625026

52 exp Exercise/ or exp Exercise Therapy/ or Exercis*.mp. or Exercise therap*.mp. [mp=title, book title, abstract, original title, name of substance word, subject heading word, floating sub-heading word, keyword heading word, organism supplementary concept word, protocol supplementary concept word, rare disease supplementary concept word, unique identifier, synonyms] 505974

53 exp Sports/ or Aerobic.mp. or Strength.mp. or Endurance.mp. or Athletic.mp. [mp=title, book title, abstract, original title, name of substance word, subject heading word, floating sub-heading word, keyword heading word, organism supplementary concept word, protocol supplementary concept word, rare disease supplementary concept word, unique identifier, synonyms] 693284

54 exp Physical Fitness/ or Physical train*.mp. or Fitness.mp. or Physical activit*.mp. [mp=title, book title, abstract, original title, name of substance word, subject heading word, floating sub-heading word, keyword heading word, organism supplementary concept word, protocol supplementary concept word, rare disease supplementary concept word, unique identifier, synonyms] 243524

55 (exercise training or progressive resistance exercise or strength training or endurance training).mp. [mp=title, book title, abstract, original title, name of substance word, subject heading word, floating sub-heading word, keyword heading word, organism supplementary concept word, protocol supplementary concept word, rare disease supplementary concept word, unique identifier, synonyms] 29917

56 (swim* or run* or jog* or gym* or walk*).mp. [mp=title, book title, abstract, original title, name of substance word, subject heading word, floating sub-heading word, keyword heading word, organism supplementary concept word, protocol supplementary concept word, rare disease supplementary concept word, unique identifier, synonyms] 433468

57 exp Massage/ or massage therap*.mp. or massag*.mp. or myotherap*.mp. [mp=title, book title, abstract, original title, name of substance word, subject heading word, floating sub-heading word, keyword heading word, organism supplementary concept word, protocol supplementary concept word, rare disease supplementary concept word, unique identifier, synonyms] 16829

58 52 or 53 or 54 or 55 or 56 or 57 1444024

59 exp Diet/ or exp Diet Therapy/ or Diet* Modif*.mp. or Diet* Restriction.mp. [mp=title, book title, abstract, original title, name of substance word, subject heading word, floating sub-heading word, keyword heading word, organism supplementary concept word, protocol supplementary concept word, rare disease supplementary concept word, unique identifier, synonyms] 332370

60 exp Food/ or Food habit*.mp. [mp=title, book title, abstract, original title, name of substance word, subject heading word, floating sub-heading word, keyword heading word, organism supplementary concept word, protocol supplementary concept word, rare disease supplementary concept word, unique identifier, synonyms] 1423961

61 (Mediterranean or DASH or Vegetarian or Vegan or Western or Paleolithic or Keto*).mp. [mp=title, book title, abstract, original title, name of substance word, subject heading word, floating sub-heading word, keyword heading word, organism supplementary concept word, protocol supplementary concept word, rare disease supplementary concept word, unique identifier, synonyms] 787260

62 59 or 60 or 61 2345247

63 (umbrella review or meta-review or meta-analy* or metaanaly* or meta reg* or metareg* or systematic review*).tw. or (umbrella review or meta-review or meta-analy* or metaanaly* or meta reg* or metareg* or systematic review*).pt. 413170

64 11 or 25 or 38 or 47 or 51 or 58 or 62 6847420

65 6 and 63 and 64 1070

66 limit 65 to (english language and yr="2012 -Current" and last 10 years) 830

### Search History for Cochrane Database of Systematic Reviews (CDSR)

ID Search Hits

#1 "Inflammatory arthritis" or monoarthritis or oligoarthritis or polyarthritis 1055

#2 [mh "Arthritis, Rheumatoid"] OR rheumatoid arthritis 19440

#3 [mh "Spondylitis, Ankylosing"] OR Ankylosing Spondyl* OR Bechterew* Disease OR spondyloarth* 3286

#4 [mh "Arthritis, Psoriatic"] OR Psoriatic arthritis OR Arthritis psoriatica 3052

#5 [mh Gout] OR gout* OR hyperur?c?emi* or [mh Chondrocalcinosis] or pseudogout or chondrocalcinosis or "Calcium Pyrophosphate Deposition Disease" or "Calcium Pyrophosphate Dihydrate Deposition" 2799

#6 ([mh "Lupus erythematosus, systemic"] OR SLE or systemic lupus erythematosus or lupus or "connective tissue disease" or CTD or [mh "Connective Tissue Diseases"] or [mh "Mixed Connective Tissue Disease"] or "mixed connective tissue disease" or MCTD) AND ([mh Arthritis] or "Inflammatory arthritis" or spondyloarth* or monoarthritis or oligoarthritis or polyarthritis or arthritis) 9366

#7 {OR #1-#6} 28258

#8 [mh "Complementary Therapies"] OR Complementary Therap* or Complementary Medic* or Alternative Therap* or Alternative Medic* 78704

#9 [mh "Integrative Medicine"] OR Integrative Medic* or Integrative Therap* 6466

#10 [mh "Holistic Health"] OR Holistic Medic* or Holistic Therap* 1523

#11 [mh "Healthy Lifestyle"] or Lifestyle Medic* or Lifestyle Interven* or Lifestyle Medic* or Lifestyle Therap* 26796

#12 {OR #8-#11} 108235

#13 [mh "Acupuncture Therapy"] or [mh "Acupuncture Points"] or [mh "Acupuncture, Ear"] or acupuncture or "Zhen Jiu" 21502

#14 [mh Needles] or needl* 22822

#15 [mh "Acupressure"] or acupoint* or acupressure 7457

#16 [mh Moxibustion] or moxibustion 6496

#17 [mh Artemisia] or mugwort 102

#18 [mh Meridians] or meridian or "meridian therapy" 3866

#19 "oriental medicine" 361

#20 {OR #13-#19} 44022

#21 [mh "Herbal Medicine"] or [mh "Drugs, Chinese Herbal"] or [mh "Traditional Chinese Medicine"] or "Traditional Chinese Medicine" 21103

#22 [mh "Medicine, East Asian Traditional"] or "Traditional East Asian medicine" 1746

#23 [mh Herbal] or [mh "Teas, Herbal"] 17

#24 [mh "Plants, Medicinal"] or Moxa 1301

#25 medicin* NEXT/5 (chines* or oriental* or Tibetan*) 3608

#26 herbal medicin* or medicin* herbal* or Plant* medicin* or medicin* plant* 15502

#27 TCM or CHM 8752

#28 {OR #21-#27} 36951

#29 [mh "Tai Chi"] or "Tai chi" or "Tai ji" 1909

#30 [mh Yoga] or yog* 8363

#31 asana* or pranayama or dharana or dhyana 857

#32 [mh "Mind Body Therapies"] or mind-body therap* 9964

#33 [mh Mindfulness] or mindfulness* 9194

#34 [mh Meditation] or meditat* 4819

#35 [mh "Relaxation Therapy"] 2562

#36 {OR #29-#35} 26573

#37 [mh "Dietary Supplements"] 17190

#38 vitamin* OR mineral* OR nutrient* OR food supplement* OR meal replacement* OR nutritional supplement* OR health supplement* OR multivitamin* 93916

#39 "omega 3" OR fish oil* OR "alpha lipoic acid" OR "alpha linolenic acid" OR "alpha linoleic acid" OR eicosapentaenoic OR docosahexaenoic OR fatty acid* OR amino acid* OR taurine OR "s adenosyl methionine" OR creatine OR acetylcysteine OR cysteine OR probiotic* OR tryptophan OR tocopherol OR alphatocopherol OR carotene OR retinol OR thiamine OR riboflavin OR niacin OR niacinamide OR nicotinic acid OR pantothenic OR pyridox* OR biotin OR methylfolate OR 5 MTH* OR "levomefolic acid" OR folate OR "folinic acid" OR "folic acid" OR inositol OR cyanocobalamin OR methylcobalamin OR cobalamin OR "ascorbic acid" OR cholecalciferol OR iron OR ferrous OR tocopherols OR "trace element" OR calcium OR phosphorus OR magnesium OR potassium OR manganese OR zinc OR selenium OR boron OR chromium OR lycopene OR isoflav* OR flavonoid* OR bioflavonoid* OR micronutrient OR carnitine 154989

#40 {OR #37-#29} 43484

#41 [mh Exercise] or [mh "Exercise Therapy"] or exercis* or exercise therap* 146989

#42 [mh Sports] or aerobic or strength or endurance or athletic 88842

#43 [mh "Physical Fitness"] or physical train* or fitness or physical activit* 97624

#44 exercise training or progressive resistance exercise or strength training or endurance training 53988

#45 swim* Or run* OR jog* OR gym* OR walk* 83636

#46 [mh Massage] or massage therap* or massag* or myotherap* 8101

#47 {OR #41-#46} 265875

#48 [mh Diet] or [mh "Diet Therapy"] or Diet* Modif* or Diet* Restriction 43492

#49 [mh Food] or Food habit* 49087

#50 Mediterranean or DASH or Vegetarian or Vegan or Western or Paleolithic or Keto* 53892

#51 {OR #48-#50} 126969

#52 #12 OR #20 or #28 or #36 or #40 or #47 or #51 480608

#53 #7 AND #52 with Cochrane Library publication date Between Jan 2012 and Dec 2022 5402

### Search History for APA PsycINFO and CINAHL Plus through EBSCOHost

S58 S8 AND S56 AND S57 Limiters - Publication Year: 2012-2022

Expanders - Apply equivalent subjects

Search modes - Boolean/Phrase

S57 S13 OR S21 OR S32 OR S40 OR S44 OR S51 OR S55

S56 AB (umbrella review OR meta-review OR meta-analy* OR metaanaly* OR meta reg* OR metareg* OR systematic review*) OR TI (umbrella review OR meta-review OR meta-analy* OR metaanaly* OR meta reg* OR metareg* OR systematic review*)

S55 S52 OR S53 OR S54

S54 Mediterranean or DASH or Vegetarian or Vegan or Western or Paleolithic or Keto*

S53 MA(Food) or Food habit*

S52 MA(Diet) or MA(Diet Therapy) or Diet* Modif* or Diet* Restriction

S51 S45 OR S46 OR S47 OR S48 OR S49 OR S50

S50 MA(Massage) or massage therap* or massag* or myotherap*

S49 swim* Or run* OR jog* OR gym* OR walk*

S48 exercise training or progressive resistance exercise or strength training or endurance training

S47 MA(Physical Fitness) or Physical train* or Fitness or Physical activit*

S46 MA(Sports) or Aerobic or Strength or Endurance or Athletic

S45 MA(Exercise) OR MA(Exercise Therapy) or Exercis* or Exercise therap*

S44 S41 OR S42 OR S43

S43 omega 3 OR fish oil* OR alpha lipoic acid OR alpha linolenic acid OR alpha linoleic acid OR eicosapentaenoic OR docosahexaenoic OR fatty acid* OR amino acid* OR taurine OR s-adenosyl methionine OR creatine OR acetylcysteine OR cysteine OR probiotic* OR tryptophan OR tocopherol OR alphatocopherol OR carotene OR retinol OR thiamine OR riboflavin OR niacin OR niacinamide OR nicotinic acid OR pantothenic OR pyridox* OR biotin OR methylfolate OR 5-MTH* OR levomefolic acid OR folate OR folinic acid OR ...

S42 vitamin* OR mineral* OR nutrient* OR food supplement* OR meal replacement* OR nutritional supplement* OR health supplement* OR multivitamin*

S41 MA(Dietary Supplements)

S40 S33 OR S34 OR S35 OR S36 OR S37 OR S38 OR S39

S39 MA(relaxation therapy)

S38 MA (Meditation) or meditat*

S37 MA (Mindfulness) or mindfulness*

S36 MA(Mind Body Therapies) or mind-body therapies

S35 Asana* or Pranayama or Dharana or Dhyana

S34 MA(Yoga) or yog*

S33 MA(Tai Chi) or Tai chi or Tai ji

S32 S22 OR S23 OR S24 OR S25 OR S26 OR S27 OR S28 OR S29 OR S30 OR S31

S31 TCM OR CHM

S30 Plant* medicin*

S29 herbal medicin* or medicin* herbal*

S28 medicin* N5 (chines* or oriental* or Tibetan*)

S27 MA(Plants, Medicinal) or moxa

S26 MA(Herbal) or MA(Teas, Herbal)

S25 MA(Medicine, East Asian Traditional) or Traditional East Asian medicine

S24 MA(Traditional Chinese Medicine) or traditional Chinese medicine or Oriental medicine

S23 MA(Drugs, Chinese Herbal)

S22 MA(Herbal medicine)

S21 S14 OR S15 OR S16 OR S17 OR S18 OR S19 OR S20

S20 MA(Meridians) or Meridian* or Meridian therapy

S19 MA(Artemisia) or mugwort

S18 MA(Moxibustion) or moxibustion

S17 MA(Acupressure) or acupoint* or acupressure or Zhen Jiu

S16 MA(Electroacupuncture) or Electroacupuncture

S15 MA(Needles) or needl*

S14 MA(Acupuncture Therapy) or MA(Acupuncture Points) or MA(Acupuncture, Ear) or acupuncture

S13 S9 OR S10 OR S11 OR S12

S12 MA(Healthy Lifestyle) or Lifestyle Medic* or Lifestyle Interven* or Lifestyle Medic* or Lifestyle Therap*

S11 MA(Holistic Health) or Holistic Medic* or Holistic Therap*

S10 MA(Integrative Medicine) or Integrative Medic* or Integrative Therap*

S9 MA(Complementary Therapies) or Complementary Therap* or Complementary Medic* or Alternative Therap* or Alternative Medic*

S8 S1 OR S2 OR S3 OR S4 OR S5 OR S6 OR S7

S7 Inflammatory arthritis or monoarthritis or oligoarthritis or polyarthritis

S6 (MA(Lupus erythematosus, systemic) or SLE or systemic lupus erythematosus or lupus or MA(Connective Tissue Diseases) or MA(Mixed Connective Tissue Disease) or connective tissue disease or MCTD) AND (MA(Arthritis) or inflammatory *arthritis or spondyloarth* or monoarthritis or oligoarthritis or polyarthritis or arthritis)

S5 MA(Gout) or hyperur?caemi* or hyperur?cemi* or MA(Chondrocalcinosis) or pseudogout or chondrocalcinosis or Calcium Pyrophosphate Deposition Disease or Calcium Pyrophosphate Dihydrate Deposition

S4 MA(Arthritis, Psoriatic) or PsA or Psoriatic arthritis or Arthritis psoriatica

S3 MA(Spondylitis, Ankylosing) or Ankylosing Spondyl* or Bechterew* Disease or spondyloarth*

S2 MA(Arthritis, Rheumatoid) or Rheumatoid arthritis

S1 Inflammatory arthritis or monoarthritis or oligoarthritis or polyarthritis

# Supplementary Tables

## Supplementary Table 1 – List of excluded studies

| Lead Author | Published Year | Title | Exclusion reason |
| --- | --- | --- | --- |
| Abdulrazaq, M.^1^ | 2017 | Effect of omega-3 polyunsaturated fatty acids on arthritic pain: A systematic review | Age criteria unclear or includes <18 |
| Ahc, Media^2^ | 2021 | Dietary Influences on Rheumatoid Disease | No systematic search |
| Akram, A.^3^ | 2021 | Impact of Change in Lifestyle and Exercise on Cognitive Function in Patients With Rheumatoid Arthritis: A Systematic Review | Non-RCTs |
| Al-Qubaeissy, K^4^ | 2013 | The effectiveness of hydrotherapy in the management of rheumatoid arthritis: a systematic review | Included data overlaps with another study |
| Alexanderson, H.^5^ | 2020 | Exercise therapy in patients with idiopathic inflammatory myopathies and systemic lupus erythematosus - A systematic literature review | Wrong patient population |
| Andrés, M.^6^ | 2021 | Dietary supplements for chronic gout | Included data overlaps with another study |
| Antico, A.^7^ | 2012 | Can supplementation with vitamin D reduce the risk or modify the course of autoimmune diseases? A systematic review of the literature | Wrong outcomes |
| Aqaeinezhad Rudbane, S. M^8^ | 2018 | The efficacy of probiotic supplementation in rheumatoid arthritis: a meta-analysis of randomized, controlled trials | No validated instrument for RoB or SR evaluation |
| Bagherniya, M^9^ | 2021 | The Clinical Use of Curcumin for the Treatment of Rheumatoid Arthritis: A Systematic Review of Clinical Trials | Included data overlaps with another study |
| Baillet, A.^10^ | 2012 | Efficacy of resistance exercises in rheumatoid arthritis: meta-analysis of randomized controlled trials | Over 10 years |
| Balchin, C^11^ | 2022 | Acute effects of exercise on pain symptoms, clinical inflammatory markers and inflammatory cytokines in people with rheumatoid arthritis: a systematic literature review | Included data overlaps with another study |
| Batterhan, Stephanie I.^12^ | 2013 | Systematic review and meta-analysis comparing land and aquatic exercise for people with hip or knee arthritis on function, mobility, and other health outcomes (reprinted from 2011) | Over 10 years |
| Bawa, F. L.^13^ | 2015 | Does mindfulness improve outcomes in patients with chronic pain? Systematic review and meta-analysis | Wrong study design |
| Bellan, M.^14^ | 2020 | Is cholecalciferol a potential disease-modifying anti-rheumatic drug for the management of rheumatoid arthritis? | No validated instrument for RoB or SR evaluation |
| Bergstra, S. A.^15^ | 2014 | A systematic review into the effectiveness of hand exercise therapy in the treatment of rheumatoid arthritis | No validated instrument for RoB or SR evaluation |
| Bird, J. K.^16^ | 2021 | The effect of long chain omega-3 polyunsaturated fatty acids on muscle mass and function in sarcopenia: A scoping systematic review and meta-analysis | Wrong patient population |
| Bloomfield, H^17^ | 2016 | Effects on Health Outcomes of a Mediterranean Diet With No Restriction on Fat Intake: A Systematic Review and Meta-analysis | Included data overlaps with another study |
| Bodur, H.^18^ | 2018 | Turkish League Against Rheumatism Consensus Report: Recommendations For Management of Axial Spondyloarthritis | No validated instrument for RoB or SR evaluation |
| Boison, D.^19^ | 2019 | Costus afer: A Systematic Review of Evidence-Based Data in support of Its Medicinal Relevance | No validated instrument for RoB or SR evaluation |
| Boniface, G.^20^ | 2020 | A systematic review exploring the evidence reported to underpin exercise dose in clinical trials of rheumatoid arthritis | Wrong outcomes |
| Boudjani, R^21^ | 2022 | Impact of different types of exercise programs on ankylosing spondylitis: a systematic review and meta-analysis | Age criteria unclear or includes <18 |
| Burghardt, R^22^ | 2019 | The impact of physical activity on serum levels of inflammatory markers in rheumatoid arthritis: a systematic literature review | Included data overlaps with another study |
| Bussing, A.^23^ | 2012 | Effects of yoga interventions on pain and pain-associated disability: a meta-analysis | Wrong patient population |
| Cai, G.^24^ | 2015 | Vitamin D in ankylosing spondylitis: review and meta-analysis | Wrong intervention |
| Cai, X.^25^ | 2018 | The bone-protecting efficiency of Chinese medicines compared with Western medicines in rheumatoid arthritis: A systematic review and meta-analysis of comparative studies | Wrong outcomes |
| Calder, P. C.^26^ | 2013 | Omega-3 polyunsaturated fatty acids and inflammatory processes: nutrition or pharmacology? | Wrong study design |
| Carneiro, S.^27^ | 2021 | Brazilian Society of Rheumatology 2020 guidelines for psoriatic arthritis | Recommendations or guidelines w/o underlying study info |
| Carsons, S. E.^28^ | 2017 | Treatment Guidelines for Rheumatologic Manifestations of Sjogren's Syndrome: Use of Biologic Agents, Management of Fatigue, and Inflammatory Musculoskeletal Pain | Wrong patient population |
| Casagrande, P. O.^29^ | 2022 | Effects of yoga on depressive symptoms, anxiety, sleep quality, and mood in patients with rheumatic diseases: systematic review and meta-analysis | Wrong outcomes |
| Charoenngam, N.^30^ | 2021 | Vitamin D and Rheumatic Diseases: A Review of Clinical Evidence | Wrong study design |
| Chen, L.^31^ | 2020 | The Efficacy and Mechanism of Chinese Herbal Medicines in Lowering Serum Uric Acid Levels: A Systematic Review | Wrong outcomes |
| Chen, P. E.^32^ | 2019 | Effectiveness of Cherries in Reducing Uric Acid and Gout: A Systematic Review | Wrong outcomes |
| Chen, X. M.^33^ | 2015 | Systemic Review and Meta-Analysis of the Clinical Efficacy and Adverse Effects of Zhengqing Fengtongning Combined with Methotrexate in Rheumatoid Arthritis | Age criteria unclear or includes <18 |
| Chen, X. M.^34^ | 2018 | Systematic review and meta-analysis of the efficacy and safety of biqi capsule in rheumatoid arthritis patients | Age criteria unclear or includes <18 |
| Choi, T. Y.^35^ | 2021 | Evidence map of cupping therapy | Wrong intervention |
| Cohen, M.^36^ | 2012 | Rosehip - an evidence based herbal medicine for inflammation and arthritis | Wrong study design |
| Cramer, H.^37^ | 2013 | Yoga for rheumatic diseases: a systematic review | Age criteria unclear or includes <18 |
| Cramp, F.^38^ | 2013 | Health behaviour change interventions for the promotion of physical activity in rheumatoid arthritis: a systematic review | Wrong outcomes |
| Cramp, F.^39^ | 2013 | Non-pharmacological interventions for fatigue in rheumatoid arthritis | Wrong outcomes |
| Crevelario De Melo, R.^40^ | 2021 | Effectiveness and safety of yoga to treat chronic and acute pain: A rapid review of systematic reviews | Wrong patient population |
| Daien, C.^41^ | 2022 | Dietary recommendations of the French Society for Rheumatology for patients with chronic inflammatory rheumatic diseases | Recommendations or guidelines w/o underlying study info |
| Daily, J^42^ | 2016 | Efficacy of Turmeric Extracts and Curcumin for Alleviating the Symptoms of Joint Arthritis: A Systematic Review and Meta-Analysis of Randomized Clinical Trials | Included data overlaps with another study |
| Demmelmaier, I.^43^ | 2018 | How Are Behavioral Theories Used in Interventions to Promote Physical Activity in Rheumatoid Arthritis? A Systematic Review | Wrong outcomes |
| Deng, K. F.^44^ | 2022 | Meta-analysis and trial sequential analysis on blood uric acid and joint function in gouty arthritis treated with fire needling therapy in comparison with western medication<sup></sup> | Age criteria unclear or includes <18 |
| Diao, M.^45^ | 2022 | Peripheral vitamin D levels in ankylosing spondylitis: A systematic review and meta-analysis | Wrong intervention |
| DiRenzo, D^46^ | 2018 | Systematic Review and Meta-analysis: Mindfulness-Based Interventions for Rheumatoid Arthritis | Included data overlaps with another study |
| Djalilova, D. M.^47^ | 2019 | Impact of Yoga on Inflammatory Biomarkers: A Systematic Review | Wrong patient population |
| Dos Santos, L. P.^48^ | 2021 | The effects of resistance training with blood flow restriction on muscle strength, muscle hypertrophy and functionality in patients with osteoarthritis and rheumatoid arthritis: A systematic review with meta-analysis | Wrong intervention |
| Ebrahimzadeh, A^49^ | 2021 | Effects of curcumin supplementation on inflammatory biomarkers in patients with Rheumatoid Arthritis and Ulcerative colitis: A systematic review and meta-analysis | Included data overlaps with another study |
| Elma, O.^50^ | 2020 | Do nutritional factors interact with chronic musculoskeletal pain? A systematic review | Wrong patient population |
| Feng, Z. T.^51^ | 2018 | A systemic review and meta-analysis of the clinical efficacy and safety of total glucosides of peony combined with methotrexate in rheumatoid arthritis | Age criteria unclear or includes <18 |
| Ford, A^52^ | 2018 | Dietary Recommendations for Adults With Psoriasis or Psoriatic Arthritis From the Medical Board of the National Psoriasis Foundation: A Systematic Review | Included data overlaps with another study |
| Forsyth, C^53^ | 2018 | The effects of the Mediterranean diet on rheumatoid arthritis prevention and treatment: a systematic review of human prospective studies | Included data overlaps with another study |
| Foulkes, A. C.^54^ | 2018 | What's new in psoriasis treatment? An analysis of systematic reviews published in 2015 | Wrong patient population |
| Fraenkel, L.^55^ | 2021 | 2021 American College of Rheumatology Guideline for the Treatment of Rheumatoid Arthritis | Wrong intervention |
| Franco, A. S.^56^ | 2017 | Vitamin D supplementation and disease activity in patients with immune-mediated rheumatic diseases: A systematic review and meta-analysis | Age criteria unclear or includes <18 |
| Gandhi, G. R.^57^ | 2021 | Anti-inflammatory natural products as potential therapeutic agents of rheumatoid arthritis: A systematic review | Animal studies |
| Garza, V^58^ | 2020 | Unconventional Pain Management for People With Systemic Lupus Erythematosus: A Systematic Literature Review | No validated instrument for RoB or SR evaluation |
| Geenen, R.^59^ | 2018 | EULAR recommendations for the health professional's approach to pain management in inflammatory arthritis and osteoarthritis | Recommendations or guidelines w/o underlying study info |
| Geneen, L. J.^60^ | 2017 | Physical activity and exercise for chronic pain in adults: an overview of Cochrane Reviews | Wrong patient population |
| Genel, F^61^ | 2020 | Health effects of a low-inflammatory diet in adults with arthritis: a systematic review and meta-analysis | Included data overlaps with another study |
| Gilbey, A.^62^ | 2012 | Health benefits of deer and elk velvet antler supplements: a systematic review of randomised controlled studies | Wrong patient population |
| Gilbey, A.^63^ | 2013 | A systematic review of reviews of systematic reviews of acupuncture | Age criteria unclear or includes <18 |
| Gillani, S. W.^64^ | 2018 | Management of diabetes and arthritis -A systematic review | Wrong patient population |
| Gioxari, A.^65^ | 2018 | Intake of omega-3 polyunsaturated fatty acids in patients with rheumatoid arthritis: A systematic review and meta-analysis | Age criteria unclear or includes <18 |
| Gualdron, A. J.^66^ | 2012 | Alternative medicine as treatment of osteoarthritis and rheumatoid arthritis. Systematic review of literature and meta-analysis | Not available in English |
| Gwinnutt, J. M.^67^ | 2022 | 2021 EULAR recommendations regarding lifestyle behaviours and work participation to prevent progression of rheumatic and musculoskeletal diseases | Recommendations or guidelines w/o underlying study info |
| Hall, A.^68^ | 2017 | Effectiveness of Tai Chi for Chronic Musculoskeletal Pain Conditions: Updated Systematic Review and Meta-Analysis | Wrong patient population |
| Hammond, A.^69^ | 2016 | The effectiveness of home hand exercise programmes in rheumatoid arthritis: a systematic review | Included data overlaps with another study |
| Harpham, C^70^ | 2022 | The effect of exercise training programs with aerobic components on C-reactive protein, erythrocyte sedimentation rate and self-assessed disease activity in people with ankylosing spondylitis: A systematic review and meta-analysis | Included data overlaps with another study |
| He, Y.^71^ | 2021 | Analysis of the Most-Cited Systematic Review or Meta-Analysis in Acupuncture Research | Wrong study design |
| Hoving, J. L.^72^ | 2014 | Non‚Äêpharmacological interventions for preventing job loss in workers with inflammatory arthritis | Wrong outcomes |
| Hu, J.^73^ | 2020 | Moxibustion for the treatment of ankylosing spondylitis: a systematic review and meta-analysis | Age criteria unclear or includes <18 |
| Hu, X.^74^ | 2020 | Effects of exercise programmes on pain, disease activity and function in ankylosing spondylitis: A meta-analysis of randomized controlled trials | Age criteria unclear or includes <18 |
| Huang, X.^75^ | 2019 | Efficacy and safety of external application of Traditional Chinese Medicine for the treatment of acute gouty arthritis: a systematic review and Meta-analysis | Wrong patient population |
| Huang, Y.^76^ | 2019 | Synergistic and Hepatoprotective Effect of Total Glucosides of Paeony on Ankylosing Spondylitis: A Systematic Review and Meta-Analysis | Age criteria unclear or includes <18 |
| Huo, X.^77^ | 2021 | Efficacy and Safety of Acupuncture with Western Medicine for Rheumatoid Arthritis: A Systematic Review and Meta-analysis | Included data overlaps with another study |
| Huston, P.^78^ | 2016 | Health benefits of tai chi: What is the evidence? | Wrong study design |
| Ilchovska, D. D.^79^ | 2021 | An Overview of the NF-kB mechanism of pathophysiology in rheumatoid arthritis, investigation of the NF-kB ligand RANKL and related nutritional interventions | Wrong outcomes |
| Imoto, A. M.^80^ | 2021 | Evidence for the efficacy of Tai Chi for treating rheumatoid arthritis: an overview of systematic reviews | Age criteria unclear or includes <18 |
| Jabbari, M.^81^ | 2022 | Is collagen supplementation friend or foe in rheumatoid arthritis and osteoarthritis? A comprehensive systematic review | Age criteria unclear or includes <18 |
| Katchamart, W.^82^ | 2017 | Evidence-based recommendations for the diagnosis and management of rheumatoid arthritis for non-rheumatologists: Integrating systematic literature research and expert opinion of the Thai Rheumatism Association | Recommendations or guidelines w/o underlying study info |
| Kazemi, A.^83^ | 2020 | Effect of probiotic and synbiotic supplementation on inflammatory markers in health and disease status: A systematic review and meta-analysis of clinical trials | Wrong patient population |
| Kelley, G. A.^84^ | 2015 | Effects of exercise on depression in adults with arthritis: a systematic review with meta-analysis of randomized controlled trials | Wrong outcomes |
| Kelley, G. A.^85^ | 2016 | Exercise reduces depressive symptoms in adults with arthritis: Evidential value | Wrong outcomes |
| Kelley, G. A.^86^ | 2018 | Aerobic Exercise and Fatigue in Rheumatoid Arthritis Participants: A Meta-Analysis Using the Minimal Important Difference Approach | Wrong study design |
| Kelley, G. A.^87^ | 2018 | Community-deliverable exercise and anxiety in adults with arthritis and other rheumatic diseases: a systematic review with meta-analysis of randomised controlled trials | Wrong outcomes |
| Kelley, G.A.^88^ | 2014 | Effects of exercise on depressive symptoms in adults with arthritis and other rheumatic disease: a systematic review of meta-analyses | Wrong outcomes |
| Kessler, J.^89^ | 2021 | Psoriatic arthritis and physical activity: a systematic review | No validated instrument for RoB or SR evaluation |
| Khabbazi, A.^90^ | 2020 | A Systematic Review of the Potential Effects of Nigella sativa on Rheumatoid Arthritis | Age criteria unclear or includes <18 |
| Klaps, S.^91^ | 2022 | The Influence of Exercise Intensity on Psychosocial Outcomes in Musculoskeletal Disorders: A Systematic Review | Wrong patient population |
| Ko, S. H.^92^ | 2019 | Lifestyle changes for treating psoriasis | Wrong patient population |
| Lane, B. ^93^ | 2022 | The effectiveness of group and home‐based exercise on psychological status in people with ankylosing spondylitis: A systematic review and meta‐analysis. | Wrong outcomes |
| Lane, B.^94^ | 2022 | The effectiveness of group and home-based exercise on psychological status in people with ankylosing spondylitis: A systematic review and meta-analysis | Wrong outcomes |
| Larkin, L.^95^ | 2014 | Correlates of physical activity in adults with rheumatoid arthritis: a systematic review | Wrong study design |
| Lee, J. A.^96^ | 2014 | Bee venom acupuncture for rheumatoid arthritis: a systematic review of randomised clinical trials | Wrong study design |
| Lee, M. S.^97^ | 2012 | Systematic reviews of t'ai chi: an overview | over 10 years |
| Lee, W^98^ | 2013 | Acupuncture for gouty arthritis: a concise report of a systematic and meta-analysis approach | Included data overlaps with another study |
| Lee, Y. H.^99^ | 2016 | Vitamin D level in rheumatoid arthritis and its correlation with the disease activity: a meta-analysis | Wrong study design |
| Lee, Y^100^ | 2012 | Omega-3 polyunsaturated fatty acids and the treatment of rheumatoid arthritis: a meta-analysis | Included data overlaps with another study |
| Li, H.^101^ | 2015 | Efficacy of tripterygium glycosides tablet in treating ankylosing spondylitis: a systematic review and meta-analysis of randomized controlled trials | Age criteria unclear or includes <18 |
| Li, J.^102^ | 2016 | Effects of acupuncture on rheumatoid arthritis: A systematic review and meta-analysis | Age criteria unclear or includes <18 |
| Li, T. P.^103^ | 2019 | Applications and potential mechanisms of herbal medicines for rheumatoid arthritis treatment: a systematic review | Wrong study design |
| Li, X.^104^ | 2019 | Therapeutic Potential of omega-3 Polyunsaturated Fatty Acids in Human Autoimmune Diseases | Wrong study design |
| Li, Z.^105^ | 2022 | Improvement of Acupuncture Therapy on Relapse of Patients with Gouty Arthritis: A Pairwise and Bayesian Network Meta-analysis | Wrong outcomes |
| Liang, H.^106^ | 2020 | The comparative efficacy of supervised- versus home-based exercise programs in patients with ankylosing spondylitis: A meta-analysis | Included data overlaps with another study |
| Liang, H.^107^ | 2021 | Efficacy and Safety of Acupuncture Combined with Herbal Medicine in Treating Gouty Arthritis: Meta-Analysis of Randomized Controlled Trials | Wrong patient population |
| Lin, J.^108^ | 2016 | Serum Vitamin D Level and Rheumatoid Arthritis Disease Activity: Review and Meta-Analysis | Wrong study design |
| Lin, S. S.^109^ | 2020 | Efficacy and Safety of Sinomenine Preparation for Ankylosing Spondylitis: A Systematic Review and Meta-Analysis of Clinical Randomized Controlled Trials | Age criteria unclear or includes <18 |
| Liu, B.^110^ | 2021 | Clinical safety of total glucosides of paeony adjuvant therapy for rheumatoid arthritis treatment: a systematic review and meta-analysis | Non-RCTs |
| Liu, W. H.^111^ | 2020 | Development trend and current situation of acupuncture-moxibustion indications | Wrong study design |
| Liu, W.^112^ | 2016 | Effects and safety of Sinomenine in treatment of rheumatoid arthritis contrast to methotrexate: a systematic review and Meta-analysis | Age criteria unclear or includes <18 |
| Liu, Y.^113^ | 2013 | Extracts of Tripterygium wilfordii Hook F in the Treatment of Rheumatoid Arthritis: A Systemic Review and Meta-Analysis of Randomised Controlled Trials | Age criteria unclear or includes <18 |
| Lowe, J.^114^ | 2020 | A systematic review of the effects of probiotic administration in inflammatory arthritis | Included data overlaps with another study |
| Ma, S. Y.^115^ | 2018 | Cupping therapy for treating ankylosing spondylitis: The evidence from systematic review and meta-analysis | Age criteria unclear or includes <18 |
| Ma, Y.^116^ | 2019 | Common trace metals in rheumatoid arthritis: A systematic review and meta-analysis | Wrong study design |
| Macedo, M.S.^117^ | 2014 | Systematic review summary - Non-pharmacological interventions for fatigue in rheumatoid arthritis | Wrong study design |
| Macfarlane, G. J.^118^ | 2012 | A systematic review of evidence for the effectiveness of practitioner-based complementary and alternative therapies in the management of rheumatic diseases: rheumatoid arthritis | Age criteria unclear or includes <18 |
| Manara, M.^119^ | 2013 | Italian Society of Rheumatology recommendations for the management of gout | Recommendations or guidelines w/o underlying study info |
| Marikar Bawa, F. L.^120^ | 2015 | Does mindfulness improve outcomes in patients with chronic pain? Systematic review and meta-analysis | Wrong patient population |
| Marques, A.^121^ | 2021 | Effectiveness of self-management interventions in inflammatory arthritis: a systematic review informing the 2021 EULAR recommendations for the implementation of self-management strategies in patients with inflammatory arthritis | Wrong intervention |
| Martins, N. A.^122^ | 2014 | Exercise and ankylosing spondylitis with New York modified criteria: a systematic review of controlled trials with meta-analysis | Non-RCTs |
| McKenna, S.^123^ | 2017 | Does exercise impact on sleep for people who have rheumatoid arthritis? A systematic review | Wrong outcomes |
| McKenna, S.^124^ | 2018 | Exercise Effects On Depressive and Anxiety Symptoms, Fatigue And Pain in Rheumatoid Arthritis: A Meta-Analysis: 1953 Board #214 May 31 3:30 PM - 5:00 PM...American College of Sports Medicine Annual Meeting, May 29-June 2, 2018, Minneapolis, Minnesota | Conference abstract only |
| Medrado, L. N.^125^ | 2022 | Effectiveness of aquatic exercise in the treatment of inflammatory arthritis: systematic review | Non-RCTs |
| Miles, E.A.^126^ | 2012 | Influence of marine n-3 polyunsaturated fatty acids on immune function and a systematic review of their effects on clinical outcomes in rheumatoid arthritis | Wrong study design |
| Millner, J. R.^127^ | 2016 | Exercise for ankylosing spondylitis: An evidence-based consensus statement | Wrong study design |
| Mohammed, A. T.^128^ | 2017 | The therapeutic effect of probiotics on rheumatoid arthritis: a systematic review and meta-analysis of randomized control trials | Age criteria unclear or includes <18 |
| Moi, J. H.^129^ | 2013 | Lifestyle interventions for acute gout | Wrong intervention |
| Moi, J. H.^130^ | 2014 | Lifestyle interventions for the treatment of gout: a summary of 2 Cochrane systematic reviews | Wrong study design |
| Musumeci, L.^131^ | 2022 | Citrus Flavonoids and Autoimmune Diseases: A Systematic Review of Clinical Studies | Wrong study design |
| Nagy, G.^132^ | 2022 | EULAR points to consider for the management of difficult-to-treat rheumatoid arthritis | Recommendations or guidelines w/o underlying study info |
| Nelson, J^133^ | 2020 | Do Interventions with Diet or Dietary Supplements Reduce the Disease Activity Score in Rheumatoid Arthritis? A Systematic Review of Randomized Controlled Trials | Included data overlaps with another study |
| Nelson, N.L.^134^ | 2017 | Massage Therapy for Pain and Function in Patients With Arthritis: A Systematic Review of Randomized Controlled Trials | Wrong patient population |
| Ng, J. Y.^135^ | 2020 | Rheumatoid arthritis and osteoarthritis clinical practice guidelines provide few complementary and alternative medicine therapy recommendations: a systematic review | Recommendations or guidelines w/o underlying study info |
| Nielsen, S. M.^136^ | 2018 | Nutritional recommendations for gout: An update from clinical epidemiology | Wrong study design |
| Nishishinya A.^137^ | 2019 | Efficacy of acupuncture in rheumatic diseases with spine involvement: Systematic review | Wrong patient population |
| O'Dwyer, T.^138^ | 2014 | Exercise therapy for spondyloarthritis: a systematic review | Non-RCTs |
| O'Dwyer, T.^139^ | 2015 | Physical activity in spondyloarthritis: a systematic review | Wrong study design |
| Orla, G.^140^ | 2020 | Modified Diet Use in Adults with Temporomandibular Disorders related to Rheumatoid Arthritis: A Systematic Review | Wrong intervention |
| Osthoff, A.R. ^141^ | 2018 | 2018 EULAR recommendations for physical activity in people with inflammatory arthritis and osteoarthritis | Recommendations or guidelines w/o underlying study info |
| Palabindala, V.^142^ | 2017 | 2017 - Guideline: In acute gout, steroids, NSAIDs, or low-dose colchicine are recommended; lifestyle changes are not supported | Recommendations or guidelines w/o underlying study info |
| Pan, X.^143^ | 2014 | Quality Assessment of Controlled Trials Evaluating Chinese Herbal Medicine in Patients With Rheumatoid Arthritis: A Systematic Review | conference abstract only |
| Pan, X.^144^ | 2017 | Systematic review of the methodological quality of controlled trials evaluating Chinese herbal medicine in patients with rheumatoid arthritis | Wrong study design |
| Parperis, K.^145^ | 2021 | Management of calcium pyrophosphate crystal deposition disease: A systematic review | Wrong intervention |
| Parvova, I.^146^ | 2019 | ANalysis of adverse drug reactions in the treatment of rheumatological diseases with biological medicinal products- A systematic review of scientific publications | Wrong intervention |
| Pei, J.^147^ | 2021 | Mindfulness-Based Cognitive Therapy for Treating Chronic Pain A Systematic Review and Meta-analysis | Wrong patient population |
| Pereira, L.^148^ | 2022 | Tailoring gut microbiota with a combination of Vitamin K and probiotics as a possible adjuvant in the treatment of rheumatic arthritis: a systematic review | Wrong study design |
| Peres, D.^149^ | 2017 | The practice of physical activity and cryotherapy in rheumatoid arthritis: systematic review | Non-RCTs |
| Peter, W.F.^150^ | 2021 | Clinical Practice Guideline for Physical Therapist Management of People With Rheumatoid Arthritis | Recommendations or guidelines w/o underlying study info |
| Phang, J. K.^151^ | 2018 | Complementary and alternative medicine for rheumatic diseases: A systematic review of randomized controlled trials | Wrong patient population |
| Philippou, E.^152^ | 2019 | Dietary intake, dietary interventions, nutrient supplements and rheumatoid arthritis: systematic review of the evidence | Wrong study design |
| Porras, M.^153^ | 2019 | Effects of Mediterranean diet on the treatment of rheumatoid arthritis | Wrong study design |
| Pourhabibi-Zarandi, F.^154^ | 2021 | Curcumin and rheumatoid arthritis: A systematic review of literature | Wrong study design |
| Qiu, R.^155^ | 2018 | Comparison of the efficacy of dispensing granules with traditional decoction: A systematic review and meta-analysis | Wrong patient population |
| Raad, T.^156^ | 2021 | Dietary Interventions with or without Omega-3 Supplementation for the Management of Rheumatoid Arthritis: A Systematic Review | Included data overlaps with another study |
| Ramos, A.^157^ | 2018 | Acupuncture for rheumatoid arthritis | No validated instrument for RoB or SR evaluation |
| Rausch Osthoff, A. K.^158^ | 2018 | Effects of exercise and physical activity promotion: Meta-analysis informing the 2018 EULAR recommendations for physical activity in people with rheumatoid arthritis, spondyloarthritis and hip/knee osteoarthritis | Wrong outcomes |
| Resende, G. G.^159^ | 2020 | The Brazilian Society of Rheumatology guidelines for axial spondyloarthritis - 2019 | Recommendations or guidelines w/o underlying study info |
| Rongen-van Dartel, S. A.^160^ | 2015 | Effect of Aerobic Exercise Training on Fatigue in Rheumatoid Arthritis: A Meta-Analysis | Wrong outcomes |
| Roodenrijs, N. M. T.^161^ | 2021 | Pharmacological and non-pharmacological therapeutic strategies in difficult-to-treat rheumatoid arthritis: a systematic literature review informing the EULAR recommendations for the management of difficult-to-treat rheumatoid arthritis | Wrong patient population |
| Sahebari, M.^162^ | 2019 | Selenium and Autoimmune Diseases: A Review Article | No validated instrument for RoB or SR evaluation |
| Salmon, Victoria E.^163^ | 2017 | Physical activity interventions for fatigue in rheumatoid arthritis: a systematic review | Wrong outcomes |
| Sanchez, P^164^ | 2022 | Efficacy of Probiotics in Rheumatoid Arthritis and Spondyloarthritis: A Systematic Review and Meta-Analysis of Randomized Controlled Trials | Included data overlaps with another study |
| Santos, E. J. F.^165^ | 2021 | Portuguese multidisciplinary recommendations for non-pharmacological and non-surgical interventions in patients with rheumatoid arthritis | Recommendations or guidelines w/o underlying study info |
| Santos, E^166^ | 2019 | Effectiveness of non-pharmacological and non-surgical interventions for rheumatoid arthritis: an umbrella review | Included data overlaps with another study |
| Saracoglu, I.^167^ | 2017 | The effectiveness of specific exercise types on cardiopulmonary functions in patients with ankylosing spondylitis: a systematic review | Wrong outcomes |
| Sawant, O.^168^ | 2021 | Management of psoriatic arthritis: An overview of synthetic, recombinant dna, monoclonal antibody and nature-derived agents | No validated instrument for RoB or SR evaluation |
| Seca, S^169^ | 2019 | Effectiveness of Acupuncture on Pain, Physical Function and Health-Related Quality of Life in Patients with Rheumatoid Arthritis: A Systematic Review of Quantitative Evidence | Included data overlaps with another study |
| Senftleber, N. K.^170^ | 2017 | Marine Oil Supplements for Arthritis Pain: A Systematic Review and Meta-Analysis of Randomized Trials | Wrong patient population |
| Sharma, M.^171^ | 2014 | Yoga as an alternative and complementary approach for arthritis: a systematic review | No validated instrument for RoB or SR evaluation |
| Shekelle, P. G.^172^ | 2017 | Management of Gout: A Systematic Review in Support of an American College of Physicians Clinical Practice Guideline | Wrong intervention |
| Shen, B.^173^ | 2019 | Effects of moxibustion on pain behaviors in patients with rheumatoid arthritis: A meta-analysis | Wrong patient population |
| Sieczkowska, S.M.^174^ | 2019 | Effect of yoga on the quality of life of patients with rheumatic diseases: Systematic review with meta-analysis | Wrong patient population |
| Sieczkowska, S.M.^175^ | 2020 | Effects of resistance training on the health-related quality of life of patients with rheumatic diseases: Systematic review with meta-analysis and meta-regression | Wrong patient population |
| Singh, J. A.^176^ | 2016 | 2015 American College of Rheumatology Guideline for the Treatment of Rheumatoid Arthritis | Wrong intervention |
| Singh, J.A.^177^ | 2019 | Special Article: 2018 American College of Rheumatology/National Psoriasis Foundation Guideline for the Treatment of Psoriatic Arthritis | Wrong patient population |
| Smolen, J. S.^178^ | 2014 | Treating spondyloarthritis, including ankylosing spondylitis and psoriatic arthritis, to target: recommendations of an international task force | Wrong intervention |
| Straube, S.^179^ | 2015 | Vitamin D for the treatment of chronic painful conditions in adults | Wrong patient population |
| Tam, L. S.^180^ | 2019 | 2018 APLAR axial spondyloarthritis treatment recommendations | Recommendations or guidelines w/o underlying study info |
| Tang, X.^181^ | 2021 | The Effect of Chinese Medicine Compound in the Treatment of Rheumatoid Arthritis on the Level of Rheumatoid Factor and Anti-Cyclic Citrullinated Peptide Antibodies: A Systematic Review and Meta-Analysis | Wrong patient population |
| Tanski, W.^182^ | 2022 | The Relationship between Fatty Acids and the Development, Course and Treatment of Rheumatoid Arthritis | Wrong study design |
| Tao, W.^183^ | 2022 | Investigation of the Clinical Efficacy of Acupuncture Combined with Traditional Chinese Medicine Fumigation in the Treatment of Rheumatoid Arthritis by Meta-Analysis | Age criteria unclear or includes <18 |
| Theodoridis, X.^184^ | 2021 | Effectiveness of oral vitamin D supplementation in lessening disease severity among patients with psoriasis: A systematic review and meta-analysis of randomized controlled trials | Wrong patient population |
| Tierney, M.^185^ | 2012 | Physical activity in rheumatoid arthritis: a systematic review | Wrong study design |
| Trinh, K.^186^ | 2022 | The Effect of Acupuncture on Hand and Wrist Pain Intensity, Functional Status, and Quality of Life in Adults: A Systematic Review | Wrong patient population |
| Tsiogkas, S. G.^187^ | 2021 | Effect of Crocus sativus (Saffron) Intake on Top of Standard Treatment, on Disease Outcomes and Comorbidities in Patients with Rheumatic Diseases: Synthesis without Meta-Analysis (SWiM) and Level of Adherence to the CONSORT Statement for Randomized Controlled Trials Delivering Herbal Medicine Interventions | Age criteria unclear or includes <18 |
| van den Berg, R.^188^ | 2012 | First update of the current evidence for the management of ankylosing spondylitis with non-pharmacological treatment and non-biologic drugs: a systematic literature review for the ASAS/EULAR management recommendations in ankylosing spondylitis | No validated instrument for RoB or SR evaluation |
| Vedder, D.^189^ | 2019 | Dietary Interventions for Gout and Effect on Cardiovascular Risk Factors: A Systematic Review | Wrong patient population |
| Verhoeven, F.^190^ | 2019 | Aerobic exercise for axial spondyloarthritis - its effects on disease activity and function as compared to standard physiotherapy: A systematic review and meta-analysis | Non-RCTs |
| Wagenaar, C. A.^191^ | 2021 | The Effect of Dietary Interventions on Chronic Inflammatory Diseases in Relation to the Microbiome: A Systematic Review | Wrong study design |
| Wang, F.^192^ | 2019 | Baguan Therapy Is an Alternative in Treating Ankylosing Spondylitis: A Systematic Review Based on Existing Randomized Controlled Trials | Age criteria unclear or includes <18 |
| Wang, H. R.^193^ | 2018 | A systematic review and meta-analysis of randomized controlled trials: Skin-patch of Chinese herbal medicine for patients with acute gouty arthritis | Wrong patient population |
| Wang, J.^194^ | 2013 | Content analysis of systematic reviews on effectiveness of traditional Chinese medicine | Wrong study design |
| Wang, X.^195^ | 2017 | Treatment of rheumatoid arthritis with combination of methotrexate and Tripterygium wilfordii: A meta-analysis | Age criteria unclear or includes <18 |
| Wang, Y.^196^ | 2018 | Integrative effect of yoga practice in patients with knee arthritis: A PRISMA-compliant meta-analysis | Wrong patient population |
| Wang, Y.^197^ | 2022 | Efficacy of Duhuo Jisheng Decoction in Treating Ankylosing Spondylitis: Clinical Evidence and Potential Mechanisms | Age criteria unclear or includes <18 |
| Ward, L.^198^ | 2013 | Yoga for functional ability, pain and psychosocial outcomes in musculoskeletal conditions: a systematic review and meta-analysis | Wrong patient population |
| Wen, Z^199^ | 2021 | Effectiveness of resistance exercises in the treatment of rheumatoid arthritis: A meta-analysis | Included data overlaps with another study |
| Wendling, D.^200^ | 2018 | 2018 update of French Society for Rheumatology (SFR) recommendations about the everyday management of patients with spondyloarthritis | Recommendations or guidelines w/o underlying study info |
| Wong, M. M.^201^ | 2016 | The Science of Salt: A Regularly Updated Systematic Review of Salt and Health Outcomes (August to November 2015) | Wrong patient population |
| Xiao, N.^202^ | 2018 | Evaluating the Efficacy and Adverse Effects of Clearing Heat and Removing Dampness Method of Traditional Chinese Medicine by Comparison with Western Medicine in Patients with Gout | Age criteria unclear or includes <18 |
| Xing, Q^203^ | 2020 | Efficacy and Safety of Integrated Traditional Chinese Medicine and Western Medicine on the Treatment of Rheumatoid Arthritis: A Meta-Analysis | Included data overlaps with another study |
| Xuan, Y.^204^ | 2020 | The Efficacy and Safety of Simple-Needling Therapy for Treating Ankylosing Spondylitis: A Systematic Review and Meta-Analysis of Randomized Controlled Trials | Age criteria unclear or includes <18 |
| Yang, J^205^ | 2020 | The Impact of Natural Product Dietary Supplements on Patients with Gout: A Systematic Review and Meta-Analysis of Randomized Controlled Trials | Included data overlaps with another study |
| Yang, Y^206^ | 2020 | Tripterygium Glycosides Combined with Leflunomide for Rheumatoid Arthritis: A Systematic Review and Meta-Analysis | Included data overlaps with another study |
| Yu, Y.^207^ | 2020 | Recommendations in clinical practice guidelines on gout: systematic review and consistency analysis | Wrong study design |
| Yuwen, Y.^208^ | 2012 | Development of clinical practice guidelines in 11 common diseases with Chinese medicine interventions in China | Wrong study design |
| Zao, A.^209^ | 2017 | The role of land and aquatic exercise in ankylosing spondylitis: a systematic review | No validated instrument for RoB or SR evaluation |
| Zeng, L.^210^ | 2022 | Curcumin and Curcuma longa Extract in the Treatment of 10 Types of Autoimmune Diseases: A Systematic Review and Meta-Analysis of 31 Randomized Controlled Trials | Wrong patient population |
| Zhang J.^211^ | 2020 | Intervention of Traditional Chinese Medicine nursing technique on joint pain in patients with rheumatoid arthritis: A Meta-analysis | Not available in English |
| Zhang, D.^212^ | 2020 | Comparative efficacy, safety and cost of oral Chinese patent medicines for rheumatoid arthritis: a Bayesian network meta-analysis | Age criteria unclear or includes <18 |
| Zhao, Q^213^ | 2020 | The effectiveness of aquatic physical therapy intervention on disease activity and function of ankylosing spondylitis patients: A meta-analysis | Included data overlaps with another study |
| Zhao, S.^214^ | 2014 | Systematic review of association between vitamin D levels and susceptibility and disease activity of ankylosing spondylitis | Wrong study design |
| Zheng, W.^215^ | 2012 | Electro-acupuncture-related adverse events: A systematic review | Non-RCTs |
| Zhou, R.^216^ | 2022 | Effect of Sham Acupuncture on Chronic Pain: A Bayesian Network Meta-analysis | Article retracted or not available |
| Zhou, Y^217^ | 2018 | The effectiveness and safety of Tripterygium wilfordii Hook. F extracts in rheumatoid arthritis: A systematic review and meta-analysis | Included data overlaps with another study |
| Zhu, X.^218^ | 2020 | Total glucosides of paeony for the treatment of rheumatoid arthritis: A methodological and reporting quality evaluation of systematic reviews and meta-analyses | Wrong outcomes |
| Zou, Y. Y.^219^ | 2020 | Traditional Chinese Eight Brocade Exercise Prescription for Ankylosing Spondylitis: A Quantitative Synthesis | Age criteria unclear or includes <18 |
| 冷雨飞^220^ | 2020 | 穴位贴敷疗法治疗类风湿性关节炎疗效的 Meta分析 | Not available in English |
| 郭亚丽^221^ | 2021 | 抗阻运动在类风湿关节炎病人中应用效果的系统评价 | Not available in English |

RCTs=randomised controlled trials; ROB=risk of bias; SR=systematic review

## Supplementary Table 2 – Characteristics of included studies

| **Author, Year, Review Type** | **Number of participants (trials)**  **Inclusion criteria by PICOS (Population, Intervention, Control, Outcome, Study design)** | **Relevant Interventions**  **(*Control type)*** | **Extracted Primary Outcome – functional status/QoL** | **Extracted Secondary Outcomes – pain, clinical response, disease activity, biomarkers, safety and tolerability** |
| --- | --- | --- | --- | --- |
| Bjork, M 2022^222^  Meta-analysis | N = NR (55 studies, 42 on RA, 11 on spondyloarthritis, 2 on PsA)  P = 18+ with RA spondyloarthritis or psoriatic arthritis based on established criteria (ACR/modified New York)  I = Physical activity or exercise as a stand-alone intervention  C = Passive (none/waitlist) or active controls (usual care, other exercise, combination therapies)  O = Primary: QoL  Secondary**:** Self-reported function  S = RCTs up to September 2020 | Cardiorespiratory, strength, or mixed exercise  Duration from 15 days to 2 years, median 3 months.  *(Active controls: alternative exercise or physical activity; inactive controls: usual care, waitlist)* | Functional status (Activity performance)  Overall QoL | Pain  Disease activity |
| Byrnes, K 2017^223^  Systematic review | N = 1120 (23 RCTs, 2 on AS)  P = Anyone from 18-70 years, non-smokers, and diagnosed with a medical condition  I = Traditional Pilates exercises with certified instructor  C =Placebo or comparator exercise/treatment  O = No outcomes specified in inclusion criteria  S = RCTs and cohort studies up to September 2020 | Interventions included a kinetic program with Pilates for 48 weeks, and Pilates alone for 12 weeks.  *(Kinetic program alone, normal routine)* | Functional status (BASFI) | Pain  Disease activity (BASDAI) |
| Daily, J 2017 ^224^  Meta-analysis | N = NR (13 RCTs)  P = Patients with RA. Excluded patients with severe deformity, systemic co-morbidities and pregnant patients.  I = *GuiZhiShaoYao-ZhiMu decoction*/GSZD decoction  C =Western medicine or other herbal medicines  O = Effectiveness, CRP, ESR, DMS, Rf, SJC, TJC, grip strength  S= RCTs up to Dec 2016 | All studies included the essential herbs: *Guizhi, Shaoyao, Zhimu.* GSZD commonly contained 9–20 g C. cassia Presl., 9–15 g P. Lactiflora, 10–30 g A. asphodeloides,  Some studies added additional herbs to the basic formulation. Herbs were decocted in water and provided 2-3 times per day. Co-interventions included analgesics, NSAID, steroids as well as in combination with DMARDs (including methotrexate and leflunomide). Duration 4-16 weeks.  *(Western medicine: NSAIDs, leflunomide, methotrexate, other Chinese herbal medicines -*  *glucosides of paeony, Tripterygium glycosides)* |  | Joint counts (TJC, SJC)  DMS  Biomarkers (CRP, ESR, Rf)  AE |
| Feng, C 2021^225^  Meta-analysis | N = 1224 (14 RCTs)  P = Patients with RA using the ACR criteria  I = *GuiZhiShaoYao-ZhiMu* decoction/GSZD decoction and methotrexate together+- other drugs  Excluded interventions with non-guideline methotrexate dosage (i.e. above 5-10mg/day), or combination with other non-pharmacotherapy interventions  C = Methotrexate +- other drugs  O = Primary: ACR20/ACR50, DAS28  S = RCTs up to July 2020 | All studies used GSZD as a water decoction in combination with methotrexate. There was variable combination with other drugs including with NSAIDs, sulfasalazine, steroids. Duration 6-24 weeks.  *(Western medicine alone)* |  | Joint counts (TJC, SJC)  DMS  Biomarkers (ESR, CRP, Rf)  AE |
| FitzGerald, J 2020^226^  Meta-analysis | N = NR (195 studies).  P = Adults (>18) with gout  I = Alcohol, purine, reduction in high fructose corn syrup, dairy protein, DASH/Dietary Approaches to Stop Hypertension, weight loss, other urate-reducing medications, Vit C, cherry extract  C = Any control  O = Gout flares, pain, tophus, patient global, health-related QoL, activity limitation, serum urate, AE  S= Controlled and observational trials up to April 2019 | One trial studied the effect of a purine-restricted diet for six months.  *(No purine restrictions)*  For nutrient supplements, interventions included:  3 trials on dairy protein for three to six months,  2 trials on vitamin C for 2 to 6 months,  1 trial on cherry extract for 6 months*.*  *(No change in diet/usual diet, no treatment or allopurinol, no supplementation)* |  | Gout flares- 6 months  Gout flares- 3 months  Disease activity (sUA reduction  Pain |
| Geng, Q 2022 ^227^  Meta-analysis | N = 892 (9 RCTs)  P = patients with RA using ACR/EULAR criteria  I = Tripterygium glycoside + methotrexate  C = methotrexate monotherapy  O = Primary: ACR20/50/70,  Secondary: SJC, TJC, ESR, RF, CRP, AE  S =RCTs up to September 2021 | Tripterygium glucoside + methotrexate. Dosage from 30-60mg/day. Duration from 3-6 months.  *(Methotrexate alone)* |  | Clinical response (ACR20, ACR50, ACR70)  Joint counts (SJC, TJC)  Biomarkers (ESR, CRP, Rf)  AE |
| Gkiouras, K 2022^228^  Meta-analysis | N = 1443 (23 RCTs)  P = Adults with RA by ACR/EULAR criteria  I = Fatty acids including eicosapentaenoic (EPA) and docosahexaenoic (DHA) acids. (n-3 fatty acids)  C = Placebo, and other interventions if co-administered in both groups  O = Primary: TJC, SJC, CRP, patient global assessment, SDAI  Secondary: pain, ESR, physician global assessment, DAS28, medication changes, AE  S =RCTs up to May 2021 | Varying EPA dosage of 78-4290mg per day, and DHA from 109 to 3052mg per day. Duration from 3-18 months  *(Placebo)* |  | Disease activity (DAS28)  Pain  Biomarkers (ESR, CRP)  Joint counts (TJC, SJC) |
| Guan, Y 2020^229^  Meta-analysis | N = 438 (6 RCTs)  P = Patients with RA  I = vitamin D supplementation as main intervention  C = standard treatment control/placebo  O = serum vit D, TJC, SJC, ESR, VAS, DAS28, CRP, parathyroid hormone/PTH  S = RCTs up to June 2020 | Vitamin D with or without calcium (1 study). Varying dosages from a single 100,000 IU dose, 300,000 IU daily, or 500-50,000 weekly for a 12 to 24 week period  (*Placebo, placebo+calcium)* |  | Disease activity (DAS28)  Pain (VAS)  Joint counts (TJC, SJC)  Biomarkers (ESR, CRP) |
| Gwinnutt 2022^230^  Umbrella review | N = NR (24 reviews and 150 original studies)  P = Adults with rheumatic and musculoskeletal disease (osteoarthritis, RA, systemic lupus erythematosus, axial spondyloarthritis, PsA, systemic sclerosis, gout)  I = Any dietary exposure or intervention, including animal products, experimental diets, fruits/vegetables/plant-based interventions, minerals, supplements, and vitamins  C = Any control  O = Disease activity, physical function, pain, fatigue, erosions, physical comorbidity, mental health, quality of life, work status  S = meta-analyses, systematic reviews, randomised controlled trials and non-randomised controlled trials up to September 2018 | In RA, very heterogenous range of therapies including mussel extracts (as meal replacement and in capsule form), collagen from pig skins, microalgae oil, herbal medicine, pomegranate extract, quercetin and rose hip powder, ambrotose complex, grape juice enriched with potassium with standard grape juice, combinations of supplements (beta-hydroxy-beta-methylbutyrate, glutamine and arginine), Vitamin B6.  Duration of intervention not reported consistently.  *(Placebo, no treatment, alternative diet/supplement*  For psoriatic arthritis:  Marine animal oils, including seal oil and Efamol oil (combination of fish oil and primrose oil). Dosage 0.24-2.4g EPA, 0.13 to 2.6g DHA, 1.1g DPA. Duration 6 weeks to 12 months. (*soy/olive oil placebo, liquid paraffin)*  Selenium/coenzyme Q10/Vitamin E  Duration: 30 days  *(placebo)* | Outcomes extracted from either included meta-analyses or the bespoke meta-analysis where available.  Functional status  Quality of life | Outcomes extracted from either included meta-analyses or the bespoke meta-analysis where available.  Pain  Disease activity  Joint counts (TJC, SJC)  DMS  Biomarkers (CRP, ESR) |
| Gwinnutt, J 2022^231^  Umbrella review | N = NR (79 reviews on exercise, 157 original studies on exercise)  For spondyloarthritis: 16 reviews, 24RCTs  P = People with rheumatic and musculoskeletal diseases (osteoarthritis, RA, systemic lupus erythematosus, axial spondyloarthritis, PsA, systemic sclerosis, gout)  I = Any exercise or weight related intervention  C = Any control  O = Disease activity, physical function, pain, fatigue, erosions, physical comorbidity, mental health, quality of life, work status  S= meta-analyses, systematic reviews, RCT and non-randomised trials up to September 2018 | In spondyloarthritis:  Aerobic exercise, aerobic + muscle strengthening, aquatic, home-based and muscle strengthening exercise was included.  Intervention duration not reported consistently.  *(No exercise, alternative exercises, mindfulness, usual care)* | Outcomes extracted from either included meta-analyses or the bespoke meta-analysis where available.  Functional status  Quality of life | Outcomes extracted from either included meta-analyses or the bespoke meta-analysis where available.  Pain  Disease activity  Biomarkers (CRP, ESR) |
| Hagen, K 2012^232^  Umbrella review | N = 24509 (9 systematic reviews of 224 trials)  Relevantly, 1 review on AS  P = Patients with musculoskeletal pain, osteoarthritis, RA, osteoporosis, AS  I = land-based exercise therapy interventions  C = any control  O = pain, physical disability or function  S = systematic reviews up to March 2012 | For AS, there was one review: Supervised home exercise program (no intervention)  1 trial, 155 patients  *(no intervention)* | Functional status | Pain |
| Han, R 2022 ^233^  Meta-analysis | N = 2441 (23RCT)  P = Adults with RA using ACR/EULAR criteria  I = Chinese herbal medicine + conventional-DMARD.  Excluded herbal medicines used in fumigation, or in combination with other traditional therapies.  C = DMARD  O = Primary: ACR20/50/70, AE  Secondary: DAS28, TJC, SJC, DMS, Grip strength, patient’s global assessment, DGA (Doctor’s global assessment), HAQ, VAS, ESR, CRP., Rf, anti-CCP antibodies  S= RCTs up to October 2020 | Very heterogenous group of CHMs, covering 99 individual herbal medicines. Duration from 4-48 weeks. Five most commonly used CHMs in the included studies (n) were Angelicae Sinensis Radix (10 trials), Paeoniae Radix Alba (10 trials), Cinnamomi Ramulus (9 trials), Glycyrrhizae Radix et Rhizoma (8 trials), and Clematidis Radix et Rhizoma (7 trials). These were in the form of mixed herb decotions (15 trials), herbal extract granules (1 trial), and manufactured herbal tablets (7 trials)  Co-interventions included NSAIDs, prednisone  *(methotrexate, leflunomide, combination of various DMARDs)* | Functional status (HAQ) | Clinical response (ACR20, ACR50, ACR70)  Pain (VAS)  Joint counts (TJC, SJC)  Biomarkers (ESR,CRP,Rf)  DMS  AE |
| Hu, H 2021^234^  Umbrella review | N = 7190 (10 systematic reviews of 97 RCTs)  P = Adults with RA  I = exercise or physical activity  C = other exercise, wait list, usual care, mobility  O = pain, function, fatigue, disease activity, ESR, cardiopulmonary function, grip strength  S = Systematic reviews of RCTs up to September 2019 | Exercise/PA interventions included aerobic, resistance, physical activity, hand exercise, aquatic  Duration and controls not described | Functional status | Pain  Disease activity  ESR |
| Jo, H 2022^235^  Meta-analysis | N = 19716 (186 RCTs)  P = Patients diagnosed with RA  I = orally administered East Asian herbal medicine with clearly described composition. Excluded combination therapies with conventional medications or other therapies. Intervention must be >4weeks duration.  C = conventional medicines, including traditional analgesics, NSAIDs, corticosteroids, and DMARDs.  O = Primary: pain (VAS, NRS, joint pain index, WOMAC, remission rate).  Secondary: TJC, SJC, ESR, CRP. DAS28, HAQ, ACR20. AE.  S = RCTs up to October 2021. Must be >4wks intervention and >30 patients. | Heterogenous group of herbal medicines. The most prescribed herbs were Glycyrrhizae Radix et Rhizoma, Angelicae Sinensis Radix, Radix Paeoniae Alba, Cinnamomi Ramulus, Saposhnikoviae Radix, Gentianae Macrophyllae Radix, Sinomeni Caulis et Rhizoma, Achyranthis Radix, Astragali Radix, Atractylodis Rhizoma Alba, Angelicae Pubescentis Radix, Cnidii Rhizoma. Duration from 4-52 weeks.  *(Analgesics, NSAIDs, corticosteroids and DMARDs, both alone and in combination)* | Functional status (HAQ) | Clinical response (ACR20)  Disease activity (DAS28)  Continuous pain intensity  Joint counts (TJC, SJC)  Biomarkers (ESR, CRP)  AE |
| Kou, H 2022^236^  Meta-analysis | N = 39845 (9 RCTs)  P = Patients with RA  I = Vitamin E supplementation +- combination with other medicines or rehabilitation training  C = non-vitamin E supplement, or other medication/rehab/care  O = total response rate, ADL, limb motor function score, limb spasticity score, pain score, safety  S = RCTs up to December 2021 | Included both Vit E alone and combined in tablets/supplements with variable dosage (90mg to 400mg daily). Some dosages were not specified. Duration 3 months.  *(Placebo, fish oil, other medications)* |  | Disease activity (DAS28)  Joint counts (TJC, SJC)  DMS  Biomarkers (ESR, CRP, Rf)  AE |
| Letarouilly J 2020^237^  Systematic review | N = 316 (6 RCTs, all for RA)  P = Patients with confirmed inflammatory rheumatic disease, including RA, spondyloarthritis and psoriatic arthritis  I = oral supplementation of spices, specifically garlic, cinnamon, curcuma, saffron or ginger.  C = any control group  O = Outcomes that reflect ‘symptoms and disease activity’  S = RCTs, systematic reviews or meta-analyses up to June 2020 | Garlic: 1000mg of garlic powder tablets;  Ginger: 750mg ginger powder BD; Cinnamon 1g BD; Saffron 100mg daily.  Duration ranged from 8 to 12 weeks.  *(Placebo)* | Functional status (HAQ) | Disease activity (DAS28)  Joint counts (TJC, SJC)  Pain (VAS)  Biomarkers (CRP, ESR) |
| Li, X 2013^238^  Meta-analysis | N = 4527 (57 RCTs)  P = Adults 18+ with gout diagnosed using ACR clinical criteria or crystal diagnosis  I = Chinese herbal medicine (decoction with fixed herbs, patent medicine, herbal extract injection) alone or in combination  C = No intervention, placebo, conventional Western medicine (NSAID, colchicine, other urate-lowering agents)  O = Pain, function, health-related QoL. Recurrence/flare, sUA, CRP, swelling/tenderness, safety.  S = RCTs up to December 2012 | Intervention groups were divided into Chinese herbal medicine alone (41 trials) and herbal and conventional medicine in combination (16 trials). Formulations include decoction, granule, capsule, pill of varying herbal compositions. The most common prescription was *Simiao* prescription. Treatment duration varied from 1-60 days.  *(Colchicine, allopurinol, NSAID, uricosuric agents, both alone and in combination)* |  | Outcomes for meta-analysis was performed only for Chinese herbal medicine alone vs. Conventional medicine group.  Disease activity (sUA reduction  Pain intensity  Biomarkers (CRP)  AE |
| Li, S 2021^239^  Network meta-analysis | N = NR (15 RCTs)  P = Patients with RA using ACR/EULAR criteria  I = One of the following Chinese herbal medicines: *Baihuguizhi* decoction (BHGZD), *Dangguiniantong* decoction (DGNTD), *Simiao* pill (SMP), *Xuanbi* decoction (XBD), in combination with a csDMARD  C = csDMARD alone  O = Primary: Effective rate, ACR20/50/70, DAS28, AE, biomarkers (ESR, CRP, RF, TNF-alpha, IL-1),  Secondary: SJC, TJC, morning stiffness time  S = RCTs up to July 2020. | *Baihuguizhi* decoction (BHGZD), *Dangguiniantong* decoction (DGNTD), *Simiao* pill (SMP), *Xuanbi* decoction (XBD), in combination with a csDMARD (methotrexate, leflunomide)  Duration ranged from 2 weeks to 4 months.  *(csDMARD alone)* |  | Clinical response (ACR20,ACR50, ACR70)  Biomarkers (CRP, ESR, Rf)  Disease activity (DAS28)  Joint counts (SJC, TJC)  Morning stiffness time/Duration of morning stiffness (MST/DMS) |
| Li, H 2022 ^240^  Meta-analysis | N = 796 (11 RCTs)  P = 18+ patients with RA using EULAR/ACR criteria  I = Needle acupuncture with or without moxibustion/electrical stimulation, or laser acupuncture  C = Same as intervention group but with no acupuncture, or sham acupuncture  O = Primary: CRP, HAQ, VAS,  RAQoL  Secondary: SJC, TJC, physician global assessment, ESR, interleukin-6  S = RCTs up to March 2022 | Heterogenous group of studies, with acupuncture as stand-alone interventions, as well as combined with exercise or medication. Acupuncture types included needle and laser.  4-22 weeks follow-up    (Methotrexate, sham, csDMARD, aerobic exercise, reflexology) | Functional status (HAQ)  Health-related quality of life (RAQoL) | Pain (VAS)  Joint counts (TJC, SJC)  Biomarkers (CRP, ESR)  AE |
| Li, H 2022 ^241^  Umbrella review | N = NR (27 reviews, including 26 meta-analyses and 1 qualitative review)  24 reviews only included RCT, 3 included mixed studies which were not included in the meta-analysis.  P = Patients with RA  I = Tripterygium Willfordii Hook F extract of any type  C = Any other treatment without TwHf  O = Clinical, physiological, caregiver/patient-reported outcomes, AE  S = Systematic reviews of any study design up to July 2021 | TwHF preparations as monotherapy or with a co-intervention such as methotrexate. Information on duration and dosage was not provided.  *(Methotrexate, leflunomide, “conventional Western medicine” as a group, Chinese patent medicine or placebo as a group, not described)* |  | Joint counts (TJC, SJC)  DMS |
| Liang, H 2015^242^  Meta-analysis | N = 1098 (6RCT)  P = Adults 18+ diagnosed with AS by a rheumatologist  I = Home-based exercise therapy (relaxation, flexibility, range of motion, stretching, strengthening, posture, respiratory exercises)  Excluded combination therapies unless with education, or general exercise advice.  C = Any control  O = BASFI, BASDAI, depression, pain  S = RCT/quasi-randomised controlled trials up to October 2014 | Variety of programs including home-based exercise, including one group-based intervention and one which was combined with education. Duration 6-24 weeks.  *(Other exercise types, medical therapy)* | Functional status (BASFI) | Disease activity (BASDAI)  Pain (VAS) |
| Liang, H 2015^243^  Meta-analysis | N = 221 (5 trials, including 3 RCT, 1 CCT, 1 unclear)  P = Adult with AS diagnosed by a rheumatologist  I = Exercise + TNF inhibitor  C = TNF inhibitor  O = BASMI, BASDAI, BASFI, chest expansion  S = RCTs or CCTs up to June 2015 | Interventions from RCTs included spa rehabilitation and incentive spirometry with conventional exercise in combination with etanercept and two unspecified TNF inhibitors. Duration 2 weeks to 12 months.  *(TNF inhibitor)* | Functional status (BASFI) | Disease activity (BASDAI) |
| Liu Y et al 2020^244^  Meta-analysis | N = 747 (9 RCTs)  P = Patients with RA diagnosed according to the 1987/2010 American Rheumatology Association guidelines  I = *Kunxian* capsule/KXC (a Tripterygium Wilfordii Hook F derivative) +- DMARD for over 12 weeks  C = DMARD  O = Primary: TJC, SJC, DMS  Secondary: ESR, CRP, Rf, anti-CCP, AE  S = RCTs up to June 2019 | TwHF-KXC as monotherapy or in combination with a DMARD (methotrexate, leflunomide, hydroxychloroquine). KXC dosage ranged from 0.9g to 1.8g per day, either as BD or TID frequency.  Duration ranged from 12 weeks to 6 months.  *(DMARDs alone)* |  | DMS  Joint counts (TJC, SJC)  Biomarkers (ESR, CRP, Rf)  AE |
| Luo, J 2017^245^  Meta-analysis | N = 1209 (8 RCTs)  P = Adults with RA using ACR/EULAR criteria  I = total glucosides of paeony (TGP) +- co-interventions, for more than 12 weeks  C = placebo, no treatment, or DMARDs  O = Primary: disease improvement or remission Secondary: AE, pain, Health-related QOL, CRP, ESR  S = RCTs up to January 2017 | Range of comparisons involving either glucosides of paeony as monotherapy or in combination with DMARDs (methotrexate, leflunomide, hydroxychloroquine, sulfasalazine).  Duration ranged from 3 months to 48 weeks. TGP dosage from 0.3-0.6g TID.  *(DMARDs alone)* | Functional status (HAQ) | Clinical response (ACR20, ACR50, ACR70)  Disease activity (DAS28)  Biomarkers (CRP, ESR)  Pain  AE |
| Lu, W 2016^246^  Meta-analysis | N = 2237 (28 RCTs)  P = Gouty arthritis diagnosed according to EULAR/ARA criteria, Mexico 2010, Netherland 2010 or Criteria of Diagnosis and Therapeutic Effect of Disease and Syndromes in TCM criteria.  I = Acupuncture therapy (including manual, electroacupuncture) +- pharmacotherapy or other traditional Chinese medicine  C = Western medicine (no restriction on drug type)  O = Clinical effect, adverse events, inflammatory markers, pain, ESR, CRP  S =RCTs up to October 2015 | Interventions included manual acupuncture or electroacupuncture. The acupuncture was applied alone or in combination with other treatment, such as Chinese herbal medicine, acupoint injection, and local blocking therapy. Frequency was daily or every two days, for 20 to 30 minutes per session, and the treatment course ranged from 5 days to  28 days.  *(Western medicine - various*  *standalone or combination*  *therapy of colchicine,*  *allopurinol, indomethacin, celecoxib, benzbromarone, meloxicam)* |  | Disease activity (sUA reduction)  Pain (VAS)  Biomarkers (ESR, CRP))  AE |
| Lu, H 2022 ^247^  Meta-analysis | N = 874 (12 RCT)  P = Patients with RA  I = Needle acupuncture with or without moxibustion/ electroacupuncture in combination with “Western medicine” (DMARDs, NSAIDs, glucocorticoids, analgesics)  Studies with herbal medicines, laser, auricular, acupressure, point-application and moxibustion alone were excluded.  C = Sham or none  O = Primary: CRP, ESR, Rf  Secondary: VAS, DAS28, SJC, TJC, MS, HAQ  S = RCTs up to January 2021 | Needle acupuncture with or without electroacupuncture, in combination with “Western medicine” which included DMARDs, NSAIDs, glucocorticoids and analgesics   3-12 weeks follow-up    (no acupuncture, sha*m)* | Functional status (HAQ) | Pain (VAS)  Disease activity: DAS28  Joint counts (TJC, SJC)  Biomarkers (CRP, ESR, Rf)  AE |
| Mudano, A 2019^248^  Meta-analysis | N = 345 (7 studies including 2 RCTs and 5 CCT)  P = Patients with RA diagnosed using ACR criteria  I = Exercise with Tai Chi instruction or principles  C = Non-exercise or alternative exercise  O = Primary: ACR20/50/70, DAS, function (HAQ), radiographic progression, AE,  Secondary: TJC, SJC, Patient global assessment, Physician global assessment, acute phase reactants, range of motion, grip  S = RCTs and CCTs (controlled clinical trials) up to September 2018 | Tai Chi duration ranged from 8-12 weeks.  *(Health/exercise info, alternative exercise)* | Functional status (HAQ) | Pain  Clinical response (ACR20)  Joint counts (TJC, SJC)  Withdrawals |
| Nguyen 2021^249^  Meta-analysis | N = NR (13 RCTs, all on RA)  P = adults with chronic inflammatory rheumatic diseases (RA, spondyloarthritis, PsA)  I = oral supplementation of vit A, B, C, D, E or K  C = placebo, standard of care, no intervention  O = clinical activity indexes, HAQ, TJC, SJC, VAS, DMS, flares. BASFI, BASDAI, PsA EULAR/ACR response criteria  S = RCTs up to June 2020 | Interventions included 4 types of vitamins: Vitamin D (including calcitriol,  cholecalciferol, alfacalcidol, 22-oxa-calcitriol in varying dosages from 6 weeks to 1 year); Vitamin E (400-1200mg daily for 12 weeks); Folic acid (5-27.5mg daily for 24 weeks to 1 year); Vitamin K (10mg/day for 8 weeks)  (*Placebo, calcium, reduced folic acid dose*) | Functional status (HAQ) | Disease activity (DAS28)  Pain  Joint counts (SJC, TJC)  DMS  AE |
| Ortolan A et al 2023^250^  Systematic review | N = NR (15 studies, including 12 interventional and 3 observational)  P = Adults >18 with spondyloarthritis (axial spondyloarthritis and psoriatic arthritis)  I = diet or dietary supplement (both generic diets and specific diet types, and supplements)  C = Not specified  O = BASDAI, BASFI, Ankylosing Spondylitis Disease Activity Score /ASDAS, TJC/SJC, DAS28, Disease Activity in PSoriatic Arthritis/DAPSA, minimal disease activity/MDA, Psoriatic Arthritis Response Criteria/PsARC, VAS for pain and global assessment, CRP, ESR  S = RCTs, before-after studies, longitudinal observational studies up to June 2022 | For psoriatic arthritis:  Interventions were a hypocaloric diet for 24 weeks  *(Free diet)*  For AS: the intervention was high dose (4.55g/day) omega-3 fatty acids.  *(Low-dose/1.95g/day omega-3 fatty acids)* |  | Disease activity (minimal disease activity achievement/MDA) |
| Ortolan, A 2023^251^  Systematic review | N = NR (107 studies; 20 on exercise including 17 RCTs/CCTs)  P = Adults with axial spondyloarthritis  I = Any non-pharmacological treatment (relevantly, exercise)  C = Other non-pharmacological treatments, same treatments in different regimen, other non-biological drug treatments, any combination, or placebo.  O = ASAS criteria, BASDAI, ASDAS, BASDAI, VAS of patient global assessment, VAS pain, BASFI, spinal mobility/BASMI, SJC, TJC, global functioning, radiological findings, AE  S = RCT, CCT, cohort studies with comparators up to January 2022. Studies must have at least 50 participants. | The type, intensity and duration of exercise were heterogeneous,  ranging from Tai Chi to high intensity exercise. Supervision ranged from none to physiotherapist supervised. Duration 8 to 24 weeks.  *(Alternative exercise, no exercise)* | Functional status (BASFI) | Disease activity (BASDAI)  Pain |
| Pecourneau, V 2018^252^  Meta-analysis | N = 331 (8 RCTs)  P = Patients with AS diagnosed according to Assessment of SpondyloArthritis international Society/ASAS or modified New York Criteria  I = Exercise (any program) with explanation of program details  C = Any control group  O = BASDAI, BASFI  S = RCTs up to May 2017 | Range of exercise programs including supervised exercise, home-based care, swimming and Pilates. Co-interventions: NSAIDs were used in 7 of the 8 trials, DMARDs in 4 of the 8 trials, and anti-TNF-a agents in 4 of the 8 trials. Frequency 1-6 sessions per week, 20-90min, for 3-16 weeks  *(Usual care, physical therapy, education)* | Functional status (BASFI) | Disease activity (BASDAI) |
| Philippou 2021^253^  Systematic review | N = NR (70 studies)  P = Patients with RA  I = Nutritional intervention, dietary supplement, fasting interventions  C = Other dietary intervention, placebo, no control  O = Lab/clinical/radiological/symptomatic indices or remission indices  S = RCTs and prospective/retrospective observational studies up to October 2018 | Evening primrose oil 2 to 12 capsules per day. Duration ranged from 12 weeks to 15 months.  *(Fish oil)* |  | Disease activity (DAS28)  Pain (VAS)  Joint count (TJC) |
| Regnaux, J 2020^254^  Meta-analysis | N = 1579 (14 RCTs included in meta-analysis)  P = Adults with AS diagnosed using modified New York criteria with critical damage to sacroiliac joint on X-ray.  Excluded non-radiographic axial spondylarthritis.  I = Any kind of exercise with or without co-interventions (planned, structured, and repeated physical activity)  Excluded general movements/activity (i.e. walking, swimming).  C = No exercise (no treatment, waiting list) or usual care  O = Primary: Physical function, pain, patient global assessment, spinal mobility, fatigue, safety  Secondary: QoL, inflammatory markers, physician global assessment, SJC  S = RCTs up to December 2018 | Interventions included both monomodal and multimodal exercise programs. Components included pain relief, breathing, cardio, flexibility/stretching, endurance, motion, proprioception, relaxation, strength training. Variable supervision (none or physio), usually 60min episodes 2-3x a week. Duration 8 to 16 weeks.  *(Usual care, no exercise)* | Functional status (BASFI)  Quality of life (ASQoL) | Pain (VAS)  Biomarkers (ESR, CRP)  AE |
| Schonenberger, K, 2021 ^255^  Meta-analysis | N = 12 studies, 7RCT  P = Adults with RA  I = Mediterranean, vegetarian, vegan, or ketogenic diet.  Excluded non-whole diet interventions (i.e. single foods/supplements)  C = ordinary omnivorous diet  O = Primary: pain (VAS)  Secondary: CRP/ESR, HAQ, DAS28, SJC, TJC, weight, BMI  S = All interventional and observational studies up to November 2021. Only RCTs were included in the meta-analysis. | Dietary interventions included anti-inflammatory + corn oil (1 trial), Mediterranean diet (1 trial), vegan diet (2 trials), vegetarian diet (2 trials)  Co-interventions included corn oil and a dynamic exercise program  Follow-up 9 weeks to 1 year  *(Western diet + corn oil, usual diet, dynamic exercise program alone)* | Functional status (HAQ) | Pain (VAS)  Index counts (SJC, TJC)  Biomarkers (ESR, CRP) |
| Sieczkowska, S 2021 ^256^  Meta-analysis | N = (21 studies, 15 included in meta-analysis, incl 6 RCT, 5 randomised non-controlled trials, 5 non-RCT, 5 BAT).  For RA: 5 studies, but only two RCTs  For AS: 13 studies, but only 3 RCTs  P = Adults with systemic lupus erythematosus, RA, spondyloarthritis, Sjogren’s, systemic sclerosis, AS, IIM, systemic vasculitis  I = Home-based physical activity intervention  C = No exercise or centre-based exercise  O = Quality of life, functional capacity, pain, inflammation + biomarkers, disease activity, adherence  S = Prospective trials up to May 2020 | Exercise interventions included combined exercise and resistance exercise (home-based)  Duration range 4-96 weeks, average ~17 weeks.  *(No exercise or centre-based exercise)* | RA:  Quality of life (RAQoL) | RA:  Disease activity (DAS28)  Pain |
| Sigaux, J 2022^257^  Meta-analysis | N = 710 (30 RCT in MA, 42RCT in SR; 3 RCT on PsA; 1 RCT on AS; 1 RCT on RA + PsA; remainder of RCTs on RA)  P = Patients with rheumatic diseases (RA, AS, PsA)  I = oral supplementation of n-3 and/or n-6 PUFA  C = any (placebo, no treatment, active comparator)  O = DMS, VAS, DAS28, TJC, SJC, HAQ, ESR, CRP  S = RCTs up to October 2020 | Interventions included fish, marine and vegetable oils containing polyunsaturated fatty acids. 3 studies reported on n-6 fatty acids, with dosages from 0.5=2.8g/day. 26 studies reported on n-3 fatty acids, with dosage from 0.3-10g/day. 1 study reported on both n-3 and n-6 fatty acids. Duration varied from 0.5 to 48 weeks.  *(Placebo, no treatment, active comparator)* | Functional status (HAQ) | Pain (VAS)  Disease activity (DAS28)  DMS  Joint counts (TJC, SJC)  Biomarkers (ESR, CRP) |
| Sobue, Y 2022^258^  Meta-analysis | N = (9 trials, 6 RCT systemic exercise, 3 RCT for upper extremity)  P = Adults with RA according to 1987/2010 criteria  I = Exercise therapy  C = Usual care, others  O = Any patient-reported outcomes (i..e HAQ, pain VAS, global VAS, Short Form Health Survery-36/SF-36) and disease activity  S = RCTs up to December 2018 | Exercise interventions included both systemic exercise (dynamic, home-based, resistance, sensorimotor, aquatic, conditioning) and hand/upper extremity focused exercise (hand strengthening and stretching, upper extremity exercise training) from 2 to 16 weeks duration  *(Usual care)* | Functional status (HAQ-DI) | Quality of life (SF-36)  Pain  Disease activity (DAS-28)  AE |
| Sun Z 2014 ^259^  Meta-analysis | N = 494 (8RCT)  P = 18+ with RA  I = Moxibustion alone or with Western medicine. Excluded studies which used other forms of traditional Chinese medicine in addition.  C = Western medicine  O = Primary: ACR 20,50,70, total response rate, Secondary: DAS28  S = All RCTs up to November 2013 | Moxibustion includes indirect, smokeless, aconite, herbal, ginger. Western medicine includes DMARDs, NSAIDs in varying combinations and dosages  3-month follow-up  *(Western medicine alone)* (5 trials)  *(Western medicine)* (3 trials) |  | Clinical response (ACR20)  Disease activity (DAS28)  AE |
| Wan, R 2022 ^260^  Network meta-analysis | N = 2115 (32RCT)  P = Diagnosed with RA according to ACR/EULAR criteria  Excluded participants with another rheumatic disease  I = Acupuncture + DMARD. Excluded participants receiving multiple TCM therapy  C = DMARD or DMARD + Acupuncture  O = Primary: DAS28  Secondary: VAS, DMS, CRP, ESR, RF, AE    S = RCTs up to October 2021 | Acupuncture interventions included moxibustion (12 trials), conventional acupuncture (8 trials), electro-acupuncture (5 trials), acupoint embedding (2 trials), fire needle (2 trials), warm needle (5 trials), auricular acupuncture (1 trial)  All combined with DMARD  4–24-week follow-up  *(Methotrexate 7.5-10mg once weekly, leflunomide 10-20mg once/twice daily, or both)* |  | Disease activity (DAS28)  Pain (VAS)  DMS  Biomarkers (ESR, CRP, Rf)  AE |
| Wang, H 2016^261^  Network=meta-analysis | N = 5255 (22 RCTs)  P = Patients with RA using ACR/EULAR criteria  I = TwHF monotherapy (specifically Tripterygium glycoside tablet and Tripterygium tablet)  C = csDMARD  O = ACR20/50/70, AE  S = RCTs up to January 2015 | TwHF monotherapies. Duration ranged from 12 weeks to 2 years.  Information on dosages was not provided.  *(Methotrexate, leflunomide, sulphasalazine ,cyclosporine, tacrolimus, minocycline, placebo)* |  | Clinical response (ACR20,ACR50, ACR70)  AE |
| Wang, J 2018^262^  Meta-analysis | N = NR (11 RCTs)  P = patients with RA  I = Tripterygium willfordii preparations of any kind without other medicine  C = Any control not including TwHF  O = Primary: Effectiveness, AE  Secondary: ESR, Rf, CRP  S = RCT or quasi-randomised controlled trials up to April 2017 | Preparations included *Xin Feng, Kun Xian, Fu Fang Lei Gong Teng* topical and others. Duration 4-24 weeks.  *(Conventional pharmacotherapies including leflunomide, methotrexate, diclofenac; placebo; other Chinese patient medicines)* |  | Biomarkers (ESR, CRP, Rf)  AE |
| Wang, H 2020^263^  Meta-analysis | N = 1349 (15 RCTs)  P = patients with RA diagnosed using ACR/EULAR criteria  I = modified *Si Miao* pill + western medicine (including DMARDs and NSAIDs)  C = DMARDs +- NSAIDs alone  O = Primary: ESR, CRP, Rf, SJC, TJC,  Secondary: effective rate, DMS, AE  S = RCTs up to April 2020 | Variable combinations of *Si Miao* pill and DMARD/NSAIDs. DMARDs included methotrexate, leflunomide, sulphasalazine, hydroxychloroquine  NSAIDs included diclofenac, meloxicam, voltaren, loxoprofen.  The frequency of the intervention herbal medicine was twice daily. 13 trials used a decoction. The dosage of Phellodendri Chinensis cortex is from 10 to 15 g and that of Coicis Semen is  from 15 to 30 g; the dosage of achyranthis bidentatae radix is  from 15 to 30 g and that of atractylodes rhizome is from 10 to 15 g. Duration from 30 to 90 days.  *(DMARDs or NSAIDs alone)* |  | Joint counts (SJC, TJC)  DMS  Biomarkers (ESR, CRP, Rf)  AE |
| Williams, M 2018 ^264^  Meta-analysis | N = 841 (7RCT)  P = Adults with RA of the hand  I = Exercise for RA of the hand  C = None, usual care, PBO, medication, surgery, other  O = Primary: Hand function, pain, grip strength, ACR50, adherence, AE  Secondary: Other hand impairment measures, HAQ, DAS28, satisfaction, costs, change in splint  S = RCT and CCTs up to June 2017 | Heterogeneous group of hand exercise interventions involving variable combinations of strengthening, stretching and dexterity training.  Mostly conducted at home, from 3 weeks to four years duration.  *(Joint protection advice, no other treatment, usual care)* | Hand function:  Medium term (3-11 months)  Long term (12 months and beyond) | Pain: Short term (2 months and below)  Medium term (3-11 months)  Long term (12 months and beyond)  Joint counts (SJC, TJC):  Medium term (3-11 months)  AE |
| Xu, X 2016^265^  Meta-analysis | N = 248 (4 RCTs)  P = Patients > 60 years with RA  I = Tripterygium glycosides +- DMARDs for 3 months. Excluded combination with other traditional therapies  C = Placebo or DMARD (matching intervention)  O = Primary: SJC, TJC,  Secondary: ESR, CRP, AE  S = RCTs up to June 2015 | Tripterygium glycosides (either 10mg/20mg TID) in addition to DMARDs (leflunomide, NSAIDs, methotrexate). Duration of 6 months.  *(Placebo, DMARDs alone)* |  | Index counts (SJC, TJC)  Biomarkers (ESR, CRP)  AE |
| Ye, X 2020^266^  Meta-analysis | N = 840 (10 RCTs)  P = Patients with RA diagnosed using ACR or physician-diagnosed criteria  I = Yoga as main intervention  C = Non-yoga or medication as long as matching between intervention/control groups  O = Major: Pain, physical function (HAQ-DI), disease activity (TJC, SJC, DAS28), inflammatory cytokines (CRP, ESR, IL-6, TNF-alpha)  Secondary: grip strength  S = RCTs up to May 2020 | Yoga types were mostly non-specific, but the following yoga types were specified in some studies:  Pranayama, Hatha, Vishwas-Raj, Patanjali’s Raj. Duration 40 days to 12 weeks. Co-interventions were used in some trials (DMARDs, NSAIDs).  *(DMARDs alone, NSAID alone, no intervention)* | Functional status (HAQ-DI) | Pain  Disease activity (DAS28)  Joint counts (SJC, TJC)  Biomarkers (ESR, CRP) |
| Ye, H 2022^267^  Meta-analysis | N = 967 (13 RCTs)  P = Rheumatoid arthritis by ACR/EULAR criteria  I = Aerobic exercise at 50-90% maximal HR  C = usual care, education, other exercise, other  O = functional ability, disease activity, joint counts, inflammatory markers, pain, aerobic capacity  S = RCTs up to January 2022 | Heterogenous group including biking, running, sports, exercise circuit. Follow-up from 2-96 weeks. Frequency ranged from 2-3 times a week, and exercise of variable duration (30-90min).  *(Usual care, ROM exercise, education, non-aerobic home-based exercise)* | Functional status (HAQ-DI) | Disease activity (DAS28)  Joint counts (TJC/SJC/RAI)  Biomarkers (CRP, ESR)  Pain  AE |
| Zeng, L 2022^268^  Meta-analysis | N = NR (34 RCTs; 10 on RA, n=632; 2 on Spondyloarthritis, n=197)  P = Inflammatory arthritis (including RA, gout, IBD-arthritis, JIA, osteoarthritis, psoriasis, RA, Spondyloarthritis)  I = Probiotic preparation, +- DMARD or other drugs  C = Same intervention without probiotic preparation  O = Disease efficacy, inflammatory indicators, AE  S = RCTs up to May 2022 | RA trials: Variable dosage (1x10&10 to 1x10&8 CFU) and species (e.g. L. casei, L. acidophilus, and others). Duration 2 weeks to 12 months. Co-interventions included DMARDs/steroids.  *(Placebo, DMARDs/steroids alone)*  Spondyloarthritis trials: Probiotics included streptococcus salivarius, B. lactis, L. acidophilus, L. salivarius, L paracasei, B. infantis, B. bifidum. Duration 12 weeks.  *(Normal diet, placebo)* | Spondyloarthritis:  Functional status (BASFI) | RA:  Disease activity (DAS28)  Joint counts (TJC, SJC)  Biomarkers (ESR, CRP)  AE  Spondyloarthritis:  Disease activity (BASDAI)  AE |
| Zeng, L 2022^269^  Meta-analysis | N = 2396 (29 RCTs; 6 on RA, 1 on gout)  P = any type of arthritis (including RA, AS, osteoarthritis, juvenile idiopathic arthritis, gout/hyperuricaemia)  I = any form of curcumin  C = any non-curcumin intervention (i.e. placebo or conventional therapy)  O = efficacy indicators, inflammatory markers, AE  S = RCTs up to February 2022 | Curcumin and Curcuma longa extract were administered in doses ranging from 120 mg to 1500 mg for a duration of 4-36 weeks. Co-interventions included diclofenac and methotrexate for trials on people with RA.  *(Placebo, diclofenac/methotrexate alone)* |  | Disease activity (DAS28)  Disease activity (sUA reduction)  Joint Counts (TJC, SJC)  Biomarkers (ESR, CRP, Rf)  AE |
| Zhang, Q 2020^270^  Meta-analysis | N = 1056 (13 RCTs)  P = Adults with gout (excluding children and pregnant women) by internationally recognised diagnostic criteria  I = Use of *Guizhi-Shaoyao-Zhimu* decoction/GSZD alone or in combination  C = Conventional treatment  O = clinical efficiency number (NPE), uric acid, ESR, CRP, IL6, AE  S = RCTs up to February 2020 | Oral concoctions of GSZD of variable herbal composition and dosages, usually given twice daily. Duration 1-24 weeks. 7 studies on GSZD as stand-alone therapy, and 6 studies in combination with conventional pharmacotherapy.  *(Colchicine, celecoxib, allopurinol, diclofenac, feuxostat and celexcoxib+colchicine combination, etoncoxib + sodabic*) |  | Disease activity (sUA reduction  Biomarkers (ESR, CRP)  AE |
| Zheng, W 2020^271^  Meta-analysis | N = 3092 (40 RCTs)  P = Patients with RA diagnosed using ACR/EULAR criteria  I = TG (Tripterygium glycoside) + DMARD  Excluded combination with other herbal medicines  C = DMARD alone  O = Primary: DMS, TJC, SJC, VAS, CRP, ESR, RF  Secondary: total clinical effective rate, adverse events  S = RCTs up to September 2020 | Varying Tripterygium glycoside dosages from 60-240mg daily. Duration 4 to 24 weeks. DMARDs (methotrexate, leflunomide) were given as a co-intervention.  *(DMARDs alone)* |  | VAS  Biomarkers (CRP, ESR, Rf)  AE |
| Zhou, L 2014^272^  Meta-analysis | N = 1402 (17 RCTs)  P = Primary gout in the phase of acute arthritis, excluding pregnant women and those with blood diseases or cancer  I = Chinese herbal decoction alone  C = Western medicine only  O = “Cure rate”, serum uric acid, ESR, CRP  S = RCTs up to June 2012 | Heterogenous range of herbal decoctions. Common herbs include atractylodes lancea (10-30g), rhizoma smilacis glabrae, (20-45g), Pseudbubus Cremastra Seupleiones. Duration 7-30 days.  *(Colchicine, allopurinol, NSAIDs, benzbromarone)* |  | Disease activity (sUA reduction  Biomarkers (ESR, CRP)  AE |
| Zhou, B 2020 ^273^  Meta-analysis | N = 337 (6RCT)  P = Patients 18+ with RA diagnosed using ACR criteria. Excluded patients with psychiatric diseases, tumours, severe organ damage, and severe RA.  I = Mindfulness, both formal programs (meditation, mindfulness breathing, yoga) and informal (natural meditation, practicing meditation)  C = Routine nursing  O = VAS, depression and anxiety scores, DAS28, CRP  S = RCTs up to September 2019 | Mindfulness-based interventions included MBSR (mindfulness based stress reduction),  MBCT (mindfulness cognitive therapy),  Vitality training program, and internal family systems. Duration 8 to 36 weeks.  *(Usual care)* |  | Disease activity (DAS28)  Pain (VAS)  Biomarkers (CRP) |

ACR= American College of Rheumatology, ACR20= American College of Rheumatology outcome measures, AE= Adverse Effects, AS= Ankylosing spondylitis, ASQoL = Ankylosing Spondylitis Quality of Life, BASDAI= Bath Ankylosing Spondylitis Disease Activity Index, BASFI= Bath Ankylosing Spondylitis Functional Index, CI= confidence Interval, CRP= C-reactive protein, csDMARD= conventional synthetic disease-modifying anti-rheumatic drug, DAS-28= disease activity score, DMARD= disease-modifying anti-rheumatic drug, EULAR= European Alliance of Associations for Rheumatology, ESR= erythrocyte sedimentation rate, HAQ= health assessment questionnaire, MD= mean difference, NS= Not significant, NSAID= Non-steroidal anti-inflammatory drug, PsA= psoriatic arthritis, RA= rheumatoid arthritis, RaQOL=rheumatoid arthritis quality of life questionnaire, RAI = Ritchie Articular Index, RCT= randomized controlled trial, RF= rheumatoid factor, RoB= risk of bias, RR= risk ratio, SJC= swollen joint count, SMD = standardized mean difference, sUA= serum uric acid, TJC= tender joint count, TwHF= Tripterygium Wilfordii Hook F, WM= western medicine, VAS= visual analogue scale.

## 2.3 Supplementary Table 3 - AMSTAR Findings

| **Lead Author, Year Published** | **Critical Flaw Item No.** | **Critical Flaws Total** | **Non-Critical Flaw Item No.** | **Non-Critical Flaws Total** | **Rating** |
| --- | --- | --- | --- | --- | --- |
| Bjork, 2022^222^ | - | 0 | 3,5,6 | 3 | **Moderate** |
| Byrnes, 2018^223^ | 2,7 | 2 | 3,6,14 | 3 | **Critically Low** |
| Daily, 2017^224^ | 7 | 1 | 3,6,10,12 | 4 | **Low** |
| Feng, 2021^225^ | 7 | 1 | 3,,10,14 | 3 | **Low** |
| Fitzgerald, 2020^226^ | 2,7 | 2 | 3,6,10,14 | 4 | **Critically Low** |
| Geng, 2022^227^ | 2,7 | 2 | 3,10,14 | 3 | **Critically Low** |
| Gkiouras, 2022^228^ | - | 0 | 3,6,10 | 3 | **Moderate** |
| Guan, 2020^229^ | 7 | 1 | 10 | 1 | **Low** |
| Han, 2022^233^ | 2,7 | 2 | 3,10 | 2 | **Critically Low** |
| Jo, 2022^235^ | 2,7 | 2 | 3,12 | 2 | **Critically Low** |
| Kou, 2022^236^ | 7 | 1 | 3,10 | 2 | **Low** |
| Letarouilly, 2020^237^ | 2,7,13,15 | 4 | 3,10 | 2 | **Critically Low** |
| Li, 2013^238^ | 2,7 | 2 | 3,5,6, 10 | 4 | **Critically Low** |
| Li, 2021^239^ | 7, | 1 | 3,5,6,10 | 4 | **Low** |
| Li, 2022^240^ | 7,13 | 2 | 3,10 | 2 | **Critically Low** |
| Liang, 2015^242^ | 2,7,15 | 3 | 5,10,12,14 | 4 | **Critically Low** |
| Liang, 2015^243^ | 2,7,11 | 3 | 1,3,8,10,12 | 5 | **Critically Low** |
| Liu, 2020^244^ | 9,15 | 2 | 3,12 | 2 | **Critically Low** |
| Luo, 2017^245^ | 2,7,13,15 | 4 | 3,10,12 | 3 | **Critically Low** |
| Lu, 2016^246^ | 7 | 1 | 3 | 1 | **Low** |
| Lu, 2022^247^ | 2,7,13 | 3 | 3,10,12 | 3 | **Critically Low** |
| Mudano, 2019^248^ | 7,11,13 | 3 | 3,10,12,14 | 4 | **Critically Low** |
| Nguyen, 2020^249^ | 11 | 1 | 0 | 0 | **Low** |
| Ortolan, 2023^250^ | 2,7,9,13,15 | 5 | 3,5,10 | 3 | **Critically Low** |
| Ortolan, 2023^251^ | 2,7,15 | 3 | 3,6,10,14 | 4 | **Critically Low** |
| Pecourneau, 2018^252^ | 7,15 | 2 | 3,10,14 | 3 | **Critically Low** |
| Philippou, 2021^253^ | 2,7,13,15 | 4 | 1,3,5,6,10,12,16 | 7 | **Critically Low** |
| Regnaux, 2019^254^ | 7,15 | 2 | 3,10,14 | 3 | **Critically Low** |
| Schonenberger, 2021^255^ | 15 | 1 | 3 | 1 | **Low** |
| Sieczkowska, 2021^256^ | 7 | 1 | 3,10 | 2 | **Low** |
| Sigaux, 2022^257^ | 7,11,15 | 3 | 3,12,14 | 3 | **Critically Low** |
| Sobue, 2022^258^ | 7 | 1 | 3,10,12 | 3 | **Low** |
| Sun, 2014^259^ | 2,7,11,13 | 4 | 3,5,6,10 | 4 | **Critically Low** |
| Wan, 2022^260^ | 2,7,11 | 3 | 3,5,10,12,14 | 5 | **Critically Low** |
| Wang, 2016^261^ | 7,13 | 2 | 3,5,10,12 | 4 | **Critically Low** |
| Wang, 2018^262^ | 7,13 | 2 | 3,10,12,14 | 4 | **Critically Low** |
| Wang, 2020^263^ | 2,7,15 | 3 | 1,3,5,10,12 | 5 | **Critically Low** |
| Williams, 2018^264^ | 7,13 | 2 | 1,3,5,10,12 | 5 | **Critically Low** |
| Xu, 2016^265^ | 2 | 1 | 3 | 1 | **Low** |
| Ye, 2020^266^ | 7 | 1 | 3,5,12 | 3 | **Low** |
| Ye, 2022^267^ | 2 | 1 | 3,10 | 2 | **Low** |
| Zeng, 2022^268^ | 7,15 | 2 | 3,6,10,12 | 4 | **Critically Low** |
| Zeng, 2022^269^ | 2,15 | 2 | 3,12 | 2 | **Critically Low** |
| Zhang, 2020^270^ | 15 | 1 | 3,12 | 2 | **Low** |
| Zheng, 2021^271^ | 2,7 | 2 | 3,6,10,12 | 4 | **Critically Low** |
| Zhou, 2014^272^ | 2,7,13 | 3 | 10,12 | 2 | **Critically Low** |
| Zhou, 2020^273^ | 2,7,13,15 | 4 | 1,3,5,10,12,14 | 6 | **Critically Low** |

## Supplementary Table 4 - Findings from studies on rheumatoid arthritis

| **Lead Author, Year Published, Study Design** | **Intervention** | **Control** | | **Primary Outcome Findings**  **(+ = GRADE performed by original author)**  **(* = GRADE unable to be completed)** | **Secondary outcome findings**  **(+ = GRADE performed by original author)**  **(* = GRADE unable to be completed)** |
| --- | --- | --- | --- | --- | --- |
| ACUPUNCTURE & MOXIBUSTION | | | | | |
| Li, H 2022 ^240^  Meta-analysis | Needle or laser acupuncture | Any (sham or oral medications) | | Functional status (HAQ):  MD = -0.18, 95% CI: −0.26 to −0.10 (7 trials, n=not reported)*  RaQoL: MD = −2.32, 95% CI:−4.40 to −0.25 (3 trials, n=not reported)* | Pain (VAS): MD = −1.00, 95% CI: −1.96 to −0.05 (6 trials, n=not reported)  TJC: MD = −1.24, 95% CI: −2.11 to −0.37 (7 trials, n=not reported)  SJC: NS (6 trials, n=not reported)  ESR: MD = −3.03, 95% CI: −5.80 to −0.26 (9 trials, n=not reported)  CRP: MD = −8.05, 95% CI:−15.01 to −1.09 (11 trials, n=not reported)  No AEs associated with acupuncture were found |
| Lu, H 2022 ^247^  Meta-analysis | Needle acupuncture + WM | WM | | Functional status (HAQ):  NS (5 trials, n=not reported; very low certainty evidence; downgraded twice for risk of bias and imprecision, once for inconsistency and indirectness) | CRP: MD = −6.299, 95% CI: −9.082 to −3.517 (11 trials, n=not reported)  ESR: MD = −6.563, 95% CI: −8.604 to −4.522 (10 trials, n=not reported)  RF: NS (5 trials, n=not reported)  VAS: MD = −1.089, 95% CI: −1.575 to −0.602 (7 trials, n=not reported)  DAS-28: MD = −0.633, 95% CI: −1.006 to −0.259 (7 trials, n=not reported)  SJC: MD = −1.921, 95% CI: −3.635 to −0.207 (6 trials, n=not reported)  TJC: MD = −1.491, 95% CI: −2.941 to −0.042 (6 trials, n=not reported) |
| Sun Z 2014 ^259^  Meta-analysis | Moxibustion^[[1]](#endnote-2)^ with WM | WM | |  | Clinical response (ACR20): NS (2 trials, n=80; very low certainty evidence; downgraded for risk of bias, inconsistency and twice for imprecision)  ACR50: RR = 1.57, 95% CI: 1.25 to 1.99 (5 trials, n=242)  ACR70: NS (1 trial, n=40)  AE: Several common adverse outcomes (nausea and vomiting,  liver injury, leucopenia, rash, etc.) from drug therapy in moxibustion combined with western medical therapies were reported (4 trials) |
| Wan, R 2022 ^260^  Network meta-analysis | Moxibustion + DMARD | DMARD | |  | Disease activity (DAS28): MD= −0.62, 95% CI: −1.00 to −0.24 (7 trials, n=not reported)*  VAS: MD = −1.01, 95% CI: −1.63 to −0.40, (6 trials, n=not reported)  DMS: NS (7 trials, n=not reported)  CRP: MD = -4.19, 95% CI: −5.49 to −2.89 (11 trials, n=not reported)  ESR: MD = −10.47, 95% CI: −13.22 to -7.73 (10 trials, n=not reported)  RF: MD = −0.50, 95% CI: −0.80 to −0.20 (10 trials, n=not reported) |
|  | Acupuncture + DMARD | DMARD | |  | Disease activity (DAS28): MD= -0.92, 95% CI: -1.61 to -0.22 (2 trials, n=not reported)*  VAS: MD = -1.56, 95% CI: −2.94 to −0.19 (3 trials, n=not reported)  DMS: MD = −0.85, 95% CI: −1.12 to −0.59 (4 trials, n=not reported)  CRP: MD = −5.44, 95% CI: −6.88 to −4.00 (7 trials, n=not reported)  ESR: MD = −8.65, 95% CI: −11.28 to −6.01 (7 trials, n=not reported)  RF: MD = −1.19, 95% CI: −1.93 to −0.45 (5 trials, n=not reported) |
|  | Electroacupuncture + DMARD | DMARD | |  | Disease activity (DAS28): MD= -2.00, 95% CI: −2.59 to −1.41 (1 trial, n=not reported)*  VAS: MD = −2.19, 95% CI: −2.77 to −1.61 (2 trials, n=not reported)  DMS: MD = −2.07, 95% CI: −2.85 to −1.29 (1 trial, n=not reported)  CRP: MD = −6.68, 95% CI: −10.91 to −2.44 (4 trials, n=not reported)  ESR: MD = −10.59, 95% CI: −12.98 to −8.20 (4 trials, n=not reported)  RF: MD = −0.62, 95% CI: −1.22 to −0.02 (2 trials, n=not reported) |
|  | Warm needle acupuncture + DMARD | DMARD | |  | Disease activity (DAS28): NS (2 trials, n=not reported)*  VAS: MD = -1.23, 95% CI: −1.92 to −0.54 (1 trial, n=not reported)  DMS: MD = −0.59, 95% CI: −0.88 to −0.29 (2 trials, n=not reported)CRP: MD = −3.40, 95% CI: −4.42 to −2.38 (3 trials, n=not reported)  ESR: MD = −8.81, 95% CI: −11.35 to −6.67 (3 trials n=not reported)  RF: MD = −0.86, 95% CI: −1.20 to −0.52 (4 trials, n=not reported) |
|  | Acupoint catgut embedding+ DMARD | DMARD | |  | Disease activity (DAS28): NS (2 trials, n=not reported)* |
|  | Auricular (ear) needle + DMARD | DMARD | |  | Disease activity (DAS28): MD= −1.24, 95% CI: −1.30 to −1.18 (1 trials, n=not reported)*  DMS: MD = −2.67, 95% CI: −3.37 to −1.96 (1 trial, n= not reported)  CRP: MD = −3.49, 95% CI: −3.77 to −3.21 (1 trial, n=not reported)  ESR: MD = −11.02, 95% CI: −12.10 to −9.94 (1 trial, n=not reported) |
|  | Pooled acupuncture types | DMARD | |  | Ten studies reported adverse reactions. On the whole, the number of adverse reactions of different acupuncture therapies combined with DMARDs was lower than that of DMARDs, and there are no serious adverse reactions reported. |
| DIET | | | | | |
| Schonenberger, K, 2021^255^  Meta-analysis | Anti-inflammatory diets (Mediterranean, vegetarian & vegan diets) | Omnivorous Diet | | Functional status (HAQ): MD= −0.20, 95% CI: −0.36 to −0.05 (4 trials, n=202; very low certainty evidence; downgraded twice for RoB, once for imprecision)+ | Pain (VAS): MD= −9.22mm, 95% CI: −14.15 to −4.29mm (7 trials, n=326)  SJC: SMD = −0.60, 95% CI: −1.08 to −0.11 (4 trials, n=214)  TJC: NS (4 trials, n=212)  ESR: NS (4 trials, n=177)  CRP: NS (5 trials, n=267) |
| EXERCISE | | | | | |
| Sieczkowska, S 2021 ^256^  Meta-analysis | Home exercise | | No/ centre-based exercise | Quality of life (RAQoL): NS (1 trial, n=152)* | Disease activity (DAS28): NS (1 trial, n=152) |
| Ye, H 2022 ^267^  Meta-analysis | Aerobic exercise | | Any | Functional status (HAQ-DI):  MD=−0.25, 95% CI: −0.38 to −0.11 (11 trials, n=735, very low certainty evidence; downgraded twice for RoB, inconsistency and once for imprecision) | Disease activity (DAS28): NS (8 trials, n=573)  Joint count (TJC/SJC/RAI:) NS (4 trials, n=305)  CRP: NS (5 trials, n=298)  ESR: NS (5 trials, n=305)  Pain (VAS/McGill): SMD= −0.46, 95% CI: −0.90 to −0.01 (4 trials, n=247)  AE: None found (6 trials, n=not reported) |
| Sobue, Y, 2022^258^  Meta-analysis | Exercise therapy (systemic and upper extremity) | | Usual care, others | Functional status (HAQ-DI): MD= −0.35, 95% CI: -0.60 to -0.10 (5 trials, n=347; very low certainty evidence; downgraded for serious risk of bias, inconsistency, imprecision.) | Pain: SMD= -2.04, 95% CI: −3.77 to −0.32 (3 trials, n=244)  DAS28: MD= NS (3 trials, n=167)  SF-36: (eight domains) (2 trials, n=162)  Functional skills: MD= 21.39, 95% CI: 13.71 to 29.07  Physical aspects: NS  Pain: MD= 13.21, 95% CI: 7.36 to 19.05  General Health: MD= 13.75, 95% CI: 5.77 to 21.73  Vitality: MD= 7.51, 95% CI: 1.41 to 13.62  Social aspects: MD= 11.04, 95% CI: 0.64 to 21.43  Emotional aspects: NS  Mental Health: MD= 10.34, 95% CI: 2.11 to 18.57 |
| Williams, M 2018 ^264^  Meta-analysis | Exercise for hand arthritis | | Any | Hand function:  Medium term (3-11 months) MD= 4.5, 95% CI: 1.58 to 7.42 (1 trial, n=449; moderate certainty evidence; downgraded one level for risk of bias (high risk of performance and detection bias in measuring self reported hand function))+    Long-term (12 months+): MD= 4.3, 95% CI: 0.86 to 7.74 (1 trial, n=438; moderate certainty evidence; downgraded one level for risk of bias (high risk of performance and detection bias in measuring self reported hand function))+ | Pain:  Short term: MD= −27.98, 95% CI −48.93 to −7.03 (2 trials, n=124).  Medium term: NS (1 trial, n=445)  Long-term: NS (1 trial, n=437)  SJC (medium term): SMD= -0.16, 95% CI: −0.34 to 0.01 (2 trials, n=492)  TJC (medium term): SMD= −0.19, 95% CI: −0.37 to −0.01 (2 trials, n=492)  AE: None found (1 trial, n=490) |
| Hu, H 2021^234^  Umbrella review + meta-analysis | Pooled exercise | | Other exercise, usual care | Functional status: SMD= −0.32, 95% CI: −0.50 to −0.14 (11 trials, n=not reported)*  GRADE unable to be conduct due to lack of individual RoB data | Pain: SMD= −0.27, 95% CI: −0.41 to −0.14 (17 reviews, n=not reported)  Disease activity: NS (8 reviews, n=not reported)  ESR: NS (8 reviews, n=not reported) |
|  | Aerobic+ strength | |  | Functional status:  SMD= −0.43, 95% CI: −0.76 to −0.10 (4 trials, n=not reported, moderate certainty evidence; downgraded for allocation/blinding in 1 trial) | Pain: NS (5 reviews, n=not reported)  Disease activity: NS (3 reviews, n=not reported)  ESR: NS (1 review, n=not reported) |
|  | Strength | |  | Functional status: NS (3 trials, n=not reported, very low certainty evidence; downgraded for sample size< 400, allocation concealment (2), blinding (1), point estimates vary widely)+ | Pain: SMD= −0.61, 95% CI: −0.98 to −0.24 (3 reviews, n=not reported)  Disease activity: NS (2 reviews, n=not reported)  ESR: SMD= −0.54, 95% CI: −0.95 to −0.12 (2 reviews, n=not reported) |
|  | Aquatic | |  | Functional status: NS (1 review, n=not reported, moderate certainty evidence; downgraded for sample size <400)+ | Pain: NS (1 review, n=not reported) |
| Bjork, M 2022 ^222^  Meta-analysis | Physical activity and exercise | | Active Control (another type of treatment/activity) | Functional status (activity performance): NS (42 trials, n=not reported; moderate certainty evidence; rationale not provided)+  Overall QoL: NS (7 trials, n=not reported; low certainty evidence; rationale not provided)+ | Pain: NS (17 trials, n=not reported) |
|  |  |  | Inactive Control (usual care/no intervention) | Functional status (activity performance): SMD= −0.25, 95% CI: −0.37 to −0.13 (29 trials, n=not reported; moderate certainty evidence; rationale not provided)+  Overall QoL: SMD= 0.50, 95% CI: 0.10 to 0.90 (5 trials, n=not reported; low certainty evidence; rationale not provided)+ | Pain: SMD = −0.24, 95% CI: −0.29 to −0.14 (14 trials, n=not reported) |
| CHINESE HERBAL MEDICINE | | | | | |
| Han, R 2022 ^233^  Meta-analysis | Chinese herbal medicine + DMARD | | DMARD | Functional status (HAQ): MD= −0.43, 95% CI: −0.60 to −0.26 (6 trials, n=818; very low certainty evidence; downgraded once for RoB, inconsistency and imprecision) | ACR20: RR= 1.36, 95% CI: 1.24 to 1.48 (10 trials, n=1075)  ACR50: RR= 1.40, 95% CI: 0.99 to 1.99 (6 trials, n=790)  ACR70: RR= 1.83 95% CI: 1.19 to 2.83 (6 trials, n=718)  Disease activity (DAS28): MD= −1.27, 95% CI: 1.84 to −0.69 (7 trials, n=896)  Pain (VAS): MD= −1.25, 95% CI: −1.63 to −0.86 (3 trials, n=332)  TJC: MD= −1.71, 95% CI: −2.41 to −1.01 (15 trials, n=1771)  SJC: MD= -1.09, 95% CI: -2.46 to 0.29 (16 trials, n=1893)  DMS: SMD= −1.07, 95% CI: −1.51 to −0.63 (13 trials, n=1360)  ESR: MD= −8.34, 95% CI: −11.70 to −4.98 (21 trials, n=2217)  CRP: SMD= −0.89, 95% CI: −1.17 to −0.61 (19 trials, n=1999)  RF: SMD = −0.89, 95% CI: −1.22 to −0.55 (16 trials, n=1529)  AE: RR= −0.40, 95% CI: −0.30 to −0.53 (19 trials, n=2011) |
| Jo, H 2022^235^  Meta-analysis | East Asian oral herbal medicine monotherapy | | Conventional pharmacotherapy | Functional status (HAQ): NS (8 trials, n=1046; very low certainty evidence; downgraded for serious bias, CI less overlapping/high heterogeneity, and 95% passes 0/1.)+ | ACR20: NS (7 trials, n=1294)  DAS-28: NS (10 trials, n-1025)  Continuous pain intensity: SMD= −1.06, 95% CI: −1.33 to −0.72 (43 trials, n=4970)  TJC: MD= −1.76, 95% CI: −2.00 to −1.51 (82 trials, n=8484)  SJC: MD= −1.49, 95% CI: −1.68 to −1.31 (83 trials, n=8476)  ESR: MD= −7.16 mm/h, 95% CI: −8.33 to −5.98 (89 trials, n=8866)  CRP: MD= −4.94 mg/L, 95% CI: −6.09 to −3.79 (74 trials, n=7252)  AE: OR= 0.2548, 95% CI: 0.15 to 0.44 (21 trials, n=2448) |
| Wang, H 2016^261^  Network-meta analysis | TwHF monotherapy | | Methotrexate |  | Clinical response (ACR20):  Indirect: OR= 15.56, 95% CI: 1.63 to 148.5  Direct: NS (number of trials not reported, n=not reported)*  Forest plots for individual comparisons were not provided.  ACR50:  Indirect: NS  Direct: NS  ACR70:  Indirect NS  Direct NS  AE:  Indirect NS  Direct NS  (number of trials not reported, n=not reported)* |
|  |  |  | Leflunomide |  | Clinical response (ACR20)  Indirect: OR= 12.09, 95% CI: 1.31 to 111.5 (number of trials not reported, n=not reported)*  ACR50: Indirect NS  ACR70: Indirect NS  AE: Indirect NS  (number of trials not reported, n=not reported)* |
|  |  |  | Sulphasalazine |  | Clinical response (ACR20)  Indirect: OR= 11.76, 95% CI: 1.21 to 113.9  Direct: OR = 3.81, 95% CI: 1.79 to 8.09 (number of trials not reported, n=not reported)*  ACR50  Indirect: NS  Direct: OR= 9.67, 95% CI: 2.69 to 34.72  ACR70  Indirect: NS  Direct: OR= 12, 95% CI: 1.49 to 96.98  AE  Indirect: NS  Direct: NS  (number of trials not reported, n=not reported)* |
|  |  |  | Cyclosporine A |  | Clinical response (ACR20)  Indirect: NS (number of trials not reported, n=not reported) *  ACR50: Indirect: NS  ACR70: Indirect: NS  AE: Indirect: NS  (number of trials not reported, n=not reported)* |
|  |  |  | Tacrolimus |  | Clinical response (ACR20)  Indirect: OR= 14.77, 95% CI: 1.59 to 137.2 (number of trials not reported, n=not reported)*  ACR50: Indirect: NS  ACR70: Indirect: NS  AE: Indirect: NS  (number of trials not reported, n=not reported)* |
|  |  |  | MINO |  | Clinical response (ACR20)  Indirect: OR= 17.04, 95% CI: 1.76 to 165.3 (number of trials not reported, n=not reported)* |
|  |  |  | Placebo |  | Clinical response (ACR20)  Indirect: OR= 33.33, 95% CI: 4.17 to 100.0  Direct: OR= 36.77, 95% CI: 1.91 to 708.0  (number of trials not reported, n=not reported)*  ACR50:  Indirect: OR= 11.11, 95% CI: 1.09 to 100.0  Direct: OR= 21.97, 95% CI: 1.15 to 419.2  ACR70:  Indirect: NS  Direct: NS  AE:  Indirect: NS  Direct: NS  (number of trials not reported, n=not reported)* |
| Geng, Q 2022 ^227^  Meta-analysis | Tripterygium glycosides + methotrexate | | Methotrexate |  | Clinical response (ACR20): RR= 1.13, 95% CI: 1.04 to 1.23 (8 trials, n=862; low certainty evidence; downgraded for RoB, imprecision) +  ACR50: RR= 1.28, 95% CI: 1.13 to 1.46 (7 trials; n=724)  ACR70: RR= 1.65, 95% CI: 1.18 to 2.31 (3 trials,n=376)  SJC: MD= −2.74, 95% CI: −3.95 to −1.54 (9 trials, n=892)  TJC: MD= −2.63, 95% CI: −3.56 to −1.69 (9 trials, n=892)  ESR: MD= −15.71, 95% CI: −21.40 to −10.01 (9 trials, n=892)  CRP: SMD= −1.00, 95% CI:−1.58 to −0.42 (9 trials, n=892)  RF: MD= −45.72, 95% CI: −74.86 to −16.58 (4 trials, n=418)  Hepatic AE: NS (4 trials, n=444)  Leukopenia AE: NS (6 trials, n=612)  Gastrointestinal AE: NS (7 trials, n=750)  Cutaneous AE: NS (4 trials, n=444)  Irregular menstruation AE: NS (3 trials, n=376) |
| Li, H 2022 ^241^  Umbrella review | TwHF monotherapy | | Leflunomide |  | SJC: NS  TJC: SMD= −2.23, 95% CI: −3.27 to −1.19 (number of trials not reported, n=not reported) |
|  |  |  | WM + Chinese patent medicine or placebo |  | SJC: NS  TJC: MD= −5.41, 95% CI: −7.46 to −3.37 (number of trials not reported, n=not reported) |
|  | TwHf + DMARD | | DMARD |  | SJC: SMD= −1.72, 95% CI: −2.04 to −1.41 (number of trials not reported, n=not reported) |
|  | TwHF + Methotrexate | | Methotrexate |  | SJC: MD= 3.01, 95% CI: 2.09 to 3.93  TJC: SMD= −1.11, 95% CI: −1.96 to −0.26  DMS: SMD= −1.51, 95% CI: −2.31 to −0.71  RF: SMD= −1.51, 95% CI: −2.31 to −0.71  (number of trials not reported, n=not reported) |
|  | TwHF + Leflunomide | | Leflunomide |  | SJC: NS  TJC: SMD= −2.97, 95% CI: −4.22 to −1.72  DMS: SMD= −2.29, 95% CI: −3.36 to -1.12  RF: SMD= −2.29, 95% CI: −3.36 to −1.12  (number of trials not reported, n=not reported) |
|  |  |  | Leflunomide + WM + Chinese patent medicine or placebo |  | SJC: NS  TJC: NS  DMS: MD= −0.29, 95% CI: −0.42 to −0.12  Rf: MD= −0.29, 95% CI: −0.42 to −0.12  (number of trials not reported, n=not reported) |
| Wang, J 2018^262^  Meta-analysis | TwHF monotherapy | | Pooled control |  | ESR: MD= −3.59, 95% CI: −6.72 to −0.46 (9 trials, n=1516)  RF: MD= −5.41, 95% CI: −7.46 to −3.37 (3 trials, n=399)  CRP: MD= −1.03, 95% CI: −1.76 to −0.29 (7 trials, n=816)  AE: RR= 0.82, 95% CI: 0.70 to 0.97 (8 trials, n=1520) |
|  |  |  | Conventional pharmacotherapy |  | AE: RR= 0.08, 95% CI: 0.67 to 0.95 (6 trials, n=1254) |
|  |  |  | Other Chinese patent medicines/placebo |  | AE: NS (2 trials, n=266) |
| Xu, X 2016^265^  Meta-analysis | TwHf + DMARD for patients aged 60+ | | DMARD |  | SJC: MD = −1.58, 95% CI: −1.64 to −1.51 (4 trials, n=248)  TJC: MD = −1.73 95% CI: −2.50 to −0.97 (4 trials, n=248)  CRP: MD = −10.23, 95% CI: −11.23 to −9.23 (4 trials, n=248)  ESR: MD = −10.74, 95% CI: −12.47 to −9.00 (4 trials, n=248)  Nausea and vomiting AE= NS (4 trials, n=248)  Liver injury AE: NS (4 trials, n=248)  Leukopaenia AE: NS (4 trials, n=248)  Rash AE: NS (2 trials, n=140) |
| Zheng, W 2020^271^  Meta-analysis | TwHF + Methotrexate | | Methotrexate |  | VAS: SMD= −1.74, 95% CI: −2.13 to −1.35 (18 trials, n=not reported)  CRP: SMD = −1.27, 95% CI: −1.53 to −1.02 (22 trials, n=not reported)  ESR: SMD= −1.76, 95% CI: −2.11 to −1.42 (27 trials, n=not reported)  RF: SMD= −1.51, 95% CI: −2.00 to −1.03 (16 trials, n=not reported) |
|  | TwHF + Leflunomide | | Leflunomide |  | VAS: SMD= −1.91, 95% CI: −2.44 to −1.38 (1 trial, n=not reported)  CRP: SMD= −2.32, 95% CI: −3.55 to −1.08 (6 trials, n=not reported)  ESR: SMD= −2.24, 95% CI: −2.98 to −1.49 (8 trials, n=not reported)  RF: SMD= −3.03, 95% CI: −3.92 to −2.14 (7 trials, n=not reported) |
|  | TwHf + DMARD | | DMARD |  | AE: NS  Serious/life-threatening AEs were not found. |
| Liu, Y 2020  Meta-Analysis^244^ | TwHF - Kunxian Capsule monotherapy | | DMARD |  | TJC: NS (1 trial,n=159)  SJC: NS (1 trial, n=159)  DMS: MD= −8.28, 95% CI: −12.80 to −3.76 (2 trials, n=219)  ESR: MD = −4.66, 95% CI: −6.35 to −2.97 (3 trials, n=299)  CRP: MD = −7.29, 95% CI: −8.63 to −5.96 (3 studies, n=299)  RF: NS (2 trials, n=239) |
|  | TwHF – Kunxian Capsule + DMARD | | DMARD |  | TJC: NS (3 trials, n=160)  SJC: MD= −1.42, 95% CI: −2.57 to −0.27 (3 trials, n=160)  DMS: MD= −12.19, 95% CI: −21.63 to −2.75 (4 trials, n=232)  ESR: MD= −7.68, 95% CI: −11.01 to−4.35 (6 trials, n=448)  CRP: MD= −3.98, 95% CI: −6.52 to −1.43 (6 trials, n=448) RF: MD= −19.07, 95% CI: −29.76 to−8.39 (5 trials, n=408) |
|  | Pooled TwHf- Kunxian Capusle +- DMARD | | DMARD |  | TJC: MD= −1.07, 95% CI: −1.95 to −0.18 (4 trials, n=319)  SJC: NS (4 trials, n=319)  DMS: MD= −9.01, 95% CI: −13.08 to −4.93 (6 trials, n=451)  ESR: MD= −5.27, 95% CI: −6.78 to −3.77 (9 trials ,n=747)  CRP: MD= −5.04, 95% CI:−7.28 to −2.80 (9 trials, n=747)  RF: NS (7 trials, n= 647)  AE: No of AEs were 44 patients in KXC group, and 53 patients in the control group. (6 trials, n=535). Gastrointestinal discomfort and abnormal liver function were most common. |
| Li, S 2021^239^  Network meta-analysis | BHGZD + csDMARD | | DMARD without Chinese herbal medicine |  | Clinical response (ACR20): RR= 2.63, 95% CI: 1.02 to 6.75 (1 trial, n=110)*  ACR50: RR= 2.63, 95% CI:1.02 to 6.75 (1 trial, n=110)  ACR70: NS (1 trial, n=110)  DAS28: NS (1 trial, n=30)  CRP: MD= −6.19, 95% CI: −8.47 to −3.91 (2 trials, n=140)  ESR: MD= −6.70, 95% CI: −9.31 to −4.08 (2 trails, n=140)  Rf: MD= −0.93, 95% CI: −1.27 to −0.58 (2 trails, n=140)  TJC: MD= −2.93, 95% CI: −3.62 to −2.24 (1 trial, n=110)  DMS: MD= −15.60, 95% CI: −18.96 to −12.24 (1 trial, n=110)  AE: 23.64% (13/55) vs csDMARD 27.42% (99/361) |
|  | DGNTD + csDMARD | |  |  | Clinical response (ACR20): NS (1 trial, n=80)*  ACR50: NS (1 trial, n=80)  ACR70: NS (1 trial, n=80)  CRP: MD= −1.91, 95% CI: −3.09 to −0.72 (2 triral,s n=120)  ESR: MD= −2.37, 95% CI: −3.74 to −1.00 (2 trials, n=120)  RF: MD= −1.21, 95% CI: −1.86 to −0.56 (2 trials, n=120)  DMS: NS (1 trial, n=60)  AE: 6.77% (9/133) vs csDMARD 27.42% (99/361) |
|  | SMPPD + csDMARD | |  |  | Disease activity (DAS28): NS (2 trials, n=135)*  CRP: MD= −6.64, 95% CI: −8.99 to −4.30 (5 trials, n=307)  ESR: MD= −9.23, 95% CI: −11.77 to −6.70 (5 trials, n=307)  RF: MD= −7.72, 95% CI: −11.38 to −4.06 (4 trials, n=250)  SJC: MD= −1.14, 95% CI: −1.73 to −0.55 (3 trials, n=190)  TJC: MD= −1.24, 95% CI: −2.19 to −0.30 (3 trials, n=190)  DMS: MD= −19.90, 95% CI: −20.99 to −18.81 (2 trials, n=112)  AE: 6.45% (8/124) vs csDMARD 27.42% (99/361) |
|  | XBDPD + DMARD | |  |  | Disease activity (DAS28): SMD= −0.88, 95% CI: −1.29 to −0.47 (1 trial, n=100)*  CRP: MD= −9.31, 95% CI: −10.99 to −7.63 (1 trial, n=100)  ESR: MD= −12.35, 95% CI: −15.22 to −9.48 (1 trial, n=100)  RF: MD= −0.54, 95% CI: −0.94 to −0.14 (1 trial, n=100)  SJC: MD= −1.97, 95% CI: −2.29 to −1.65 (1 trial, n=100)  TJC: MD= −3.86, 95% CI: −4.52 to −3.20 (1 trial, n=100)  DMS: MD= −11.96, 95% CI: −14.81 to −9.11 (2 t rials, n=206)  Intervention AE incidence: 26.79% (15/56) vs csDMARD AE incidence 27.42% (99/361) |
| Wang, H 2020 ^263^  Meta-analysis | *Si Miao* Pill +WM | | WM |  | ESR: MD= −10.61, 95% CI: −12.19 to −9.03 (11 trials, n=892)  CRP: MD= −6.50, 95% CI: −8.43 to −4.56 (7 trials, n=477)  RF: MD= −17.31, 95% CI: −24.34 to −10.27 (7 trials, n=607)  SJC: MD= −1.63, 95% CI: −2.29 to −0.97 (6 trials, n=566)  TJC: MD= −1.98, 95% CI: −2.34 to −1.62 (4 trials, n=348)  DMS: MD= −24.37, 95% CI: −29.41 to -19.33 (5 trials, n=428)  AE: OR= 0.49, 95% CI: 0.30 to 0.81 (8 trials, n=651) |
| Daily, J 2017 ^224^  Meta-analysis | Guizhi-Shaoyao-Zhimu decoction +- conventional medication | | Any control (NSAIDs, analgesics, DMARDs, other herbal medicines) |  | CRP: NS  ESR: SMD= −4.91 95% CI: −8.72 to −1.11  DMS: SMD= −14.8, 95% CI: −27.3 to −2.39  RF: NS  SJC: SMD= −0.89, 95% CI: −1.40 to −0.38  TJC: NS  (no of trials not reported, n=not reported)  AE: 8 trials reported AE in control groups. 2 trials reported AE in intervention group. (n=not reported) |
| Feng, C 2021^225^  Meta-analysis | Guizhi-Shaoyao-Zhimu decoction + Methotrexate +- DMARDs/NSAIDs/ steroids | | Methotrexate +- DMARDs/NSAIDs/ steroids |  | TJC: SMD= –0.93, 95% CI: –1.28 to –0.57 (3 trials, n=not reported)  SJC: SMD= –0.81, 95% CI: –1.05 to –0.57 (4 trials, n=not reported)  DMS: SMD= –1.58, 95% CI: –2.38 to –0.78 (7 trials, n=not reported)  ESR: SMD= –1.52, 95% CI:–2.10 to –0.93 (10 trials, n=not reported)  CRP: SMD= –1.08, 95% CI: –1.48 to 0.68 (9 trials, n=not reported)  RF: SMD= −1.36, 95% CI: –2.14 to –0.58 (7 trials, n=not reported)  Safety:  Total AE: RR= 0.46, 95% CI: 0.26 to 0.83 (7 trials, n=615)  Gastrointestinal AE: RR= 0.46, 95% CI: 0.24 to 0.88 (6 trials, n= not reported)  Liver AE: NS (4 trials, n=not reported)  Neurological AE: NS (2 trials, n=not reported)  Other AE: NS (2 trials, n=not reported) |
| Luo, J 2017^245^  Meta-analysis | Glucosides of paeony + DMARD | | DMARD | Functional status (HAQ) MD = 0.4, 95% CI:-0.15 to -0.65 (1 trial, n=90; low certainty of evidence; downgraded for high risk of bias, sample size was not optimal.)+  We note a discrepancy where the authors reported that HAQ improved in the intervention group however the mean difference suggests a deterioration in functional status. | ACR20: RR =1.25, 95% CI: 1.13 to 1.38 (4 trials, n=329)  ACR50: RR= 1.29, 95% CI: 1.09 to 1.53 (4 trials, n=329)  ACR70: RR= 1.67, 95% CI: 1.15 to 2.43 (3 trials, n=269)  DAS28: MD= −0.32, 95% CI: −0.62 to −0.02 (1 trial, n=194)  Pain: MD = 1.3, 95% CI: 0.4 to 2.2 (1 trial, n=90)  CRP: NS (1 trial, n=90)  ESR: MD = 9.75, 95% CI: 0.57 to 18.92 (2 trials, n=284)  AE: RR= 0.64, 95% CI: 0.50 to 0.83 (7 trials, n=654)  We note a discrepancy where the authors reported that pain and ESR improved in the intervention group however the mean difference suggests a deterioration. |
| OTHER HERBAL MEDICINES | | | | | |
| Letarouilly J 2020^237^  Systematic review | Garlic | | Placebo | Functional status (HAQ): NS (1 trial, n=70, low risk of bias)* | One trial reported improvements in DAS-28, tender joint count (TJC), pain (VAS), and CRP. The reduction in VAS not clinically significant. The mean variation of DAS-28 ESR in the garlic group (−0.8) corresponded to a moderate EULAR response. There was no difference between the group regarding SJC, and ESR (1 trial, n=70) |
|  | Ginger | | Placebo |  | Disease activity (DAS-28) 1/1 trial reported a “significant decrease”(1 trial, n=63, low risk of bias)*  There were significant decreases in the ginger group compared to the placebo group regarding DAS28, ESR and CRP. (1 trial, n=63) |
|  | Cinnamon | | Placebo |  | Disease activity (DAS-28): 1/1 trial reported a “significant decrease” (1 trial, n=36, low risk of bias)*  “There was a significant decrease in the (cinnamon) group comparedto the placebo group regarding DAS-28, SJC, TJC, VAS pain, and CRP. Variations in DAS-28 were important in the treated group, with a mean variation corresponding to a high EULAR response. On the contrary, there was no significant difference between the two groups regarding ESR.”(1 trial, n=36) |
|  | Saffron | | Placebo |  | Disease activity (DAS-28): 1/1 trial reported a “significant decrease” (1 trial, n=66)*  “There was a significant decrease in the (saffron) group compared to the placebo group regarding DAS-28 ESR, SJC, VAS pain, and ESR. On the contrary, there was no significant difference between the two groups regarding TJC, morning stiffness, and CRP. The mean variation in DAS-28 corresponded to a moderate EULAR response.” (1 trial, n=66) |
| Zeng, L 2022^269^  Meta-analysis | Curcumin | | Any non-curcumin |  | Disease activity (DAS28): MD= −1.06, 95% CI: −1.53 to −0.59 (5 trials, n= 275; very low certainty evidence; downgraded twice for RoB, once for inconsistency and imprecision)  TJC: NS (2 trials, n=85)  SJC: NS (2 trials, n=85)  ESR: SMD= −3.09, 95% CI: −4.60 to −1.58 (5 trials, n=191)  CRP: MD= −0.35, 95% CI: −0.55 to −0.15 (4 trials, n=104)  RF: MD= −51.30, 95% CI: −60.59 to −42.01 (2 trials, n=60)  AE: NS (4 trials, n=251) |
| Philippou 2021^253^  Systematic review | Evening primrose oil | | Any |  | Disease activity (DAS-28) – “Significant decreases in Disease Activity Score-28 (DAS28) in the … groups taking … evening primrose oil, were demonstrated, as well as a reduction in the number of painful joints and pain assessed by visual analog scales.” (2 trials, n=not reported)* |
| Gwinnutt 2022^230^  Umbrella review | Pomegranate | | Placebo | Functional status NS (1 trial, n=not reported; very low certainty of evidence; rationale not provided)+ | Pain: NS  TJC, SJC: NS  CRP, ESR: NS  (1 trial, n=not reported) |
|  | Ginger + curcumin + black pepper (Curcumex) | | Placebo |  | Disease activity: SMD= −2.74, 95% CI: −3.45 to −2.03 (1 trial, n=not reported; very low certainty evidence; rationale not provided)+  TJC: SMD= −2.75, 95% CI: −3.46 to −2.03  SJC: SMD= −2.14, 95% CI: −2.77 to −1.50  ESR: SMD= −1.05, 95% CI: −1.60 to −0.51  (1 trial, n=not reported) |
|  | Ambrotose complex  (aloe vera extract) | | Placebo | Functional status: NS (1 trial, n=not reported; low certainty evidence; rationale not provided)+  QoL: NS (1 trial, n=not reported)* | Pain: NS  (1 trial, n=not reported) |
|  | Rose hip powder | | Placebo | Functional status: NS (1 trial, n=not reported; low certainty evidence; rationale not provided)+  QoL: NS (1 trial, n=not reported;low certainty of evidence; rationale not provided)+ | Pain: NS  Disease activity: NS  (1 trial, n=not reported) |
| NUTRIENT SUPPLEMENTS | | | | | |
| Zeng, L 2022^268^  Meta-analysis | Probiotics | | Any w/o probiotic |  | Disease activity (DAS28):  NS (4 trials, n=243; very low certainty evidence; downgraded for RoB, twice for inconsistency, twice for imprecision)  TJC: NS (4 trials, n=210)  SJC: NS (4 trials, n=210)  ESR: NS (3 trials, n=150)  CRP: SMD= −1.57, 95% CI: −2.98 to −0.15 (5 trials, n=264)  AE: no serious AEs found. Otherwise, mainly gastrointestinal adverse events. (5 trials, n=not reported) |
| Nguyen, Y 2021 ^249^  Meta-analysis | Vit E | | Placebo |  | Pain (VAS): NS (2 trials, n=85) |
|  | Folic acid | | Placebo | Functional status (HAQ): NS (1 double-blind trial, n=94)* | DAS28: NS (1 trial, n=40)  AE: NS (1 trial, n=40)  TJC: NS (1 trial, n=94)  SJC: NS (1 trial, n=94) |
|  | Vitamin K | | Placebo | Disease activity DAS28: NS (1 double-blind trial, n=64)* |  |
| Guan, Y 2020^229^  Meta-analysis | Vitamin D | | Placebo, placebo+calcium |  | Disease activity (DAS28): MD=−0.41, 95% CI: −0.59 to −0.23 (5 trials, n=416; low certainty evidence; serious bias (reporting, attrition), serious inconsistency)+  Pain (VAS): NS (5 trials, n=416)  TJC: MD= −1.44, 95% CI: −2.74 to −0.14 (2 trials, n=200)  SJC: NS (2 trials, n=200)  ESR: MD= −3.40, 95% CI: −6.62 to −0.18 (4 trials, n=314)  CRP: NS (4 trials, n=318) |
| Kou, H 2022^236^  Meta-analysis | Vitamin E, alone or in combination | | Placebo, fish oil, other medications |  | Disease activity (DAS28): NS (2 trials, n=104; low certainty evidence; downgraded one level of serious ROB, and two levels for very serious imprecision)  ESR: NS (5 trials, n=259)  CRP: NS 5 trials, n=259)  RF: NS (2 trials, n=70)  TJC: NS (3 trials, n=189)  SJC: NS (4 trials, n=216)  DMS: NS (3 trials, n=131)  AE: NS (2 trials, n=127) |
| Gkiouras, K 2022^228^  Meta-analysis | Omega-3 Fatty Acids | | Placebo |  | Disease activity (DAS28):  NS (5 trials, n=366; very low certainty evidence; serious risk of bias, serious risk of inconsistencies, very serious imprecision.)+  TJC: NS (8 trials, n=315)  SJC: NS (7 trials, n=276)  Pain: NS (12 trials, n=556)  CRP: NS (11 trials, n=554)  ESR: NS (9 trials, n=332) |
| Sigaux, J 2022 ^257^  Meta-analysis | Omega-3 or omega-6 PUFA | | Any control | Functional status (HAQ) (3-month): SMD= −1.48, 95% CI: 2.92 to – 0.04 (3 trials, n=204; very low certainty; downgraded for RoB, twice for inconsistency, once for imprecision)  Functional status (HAQ) (6-month): SMD= −0.67, 95% CI: −0.97 to −0.37 (3 trials, n=184; low certainty; downgraded for RoB and imprecision) | Overall pooled effects:  TJC: SMD= −0.39, 95% CI: −0.63 to −0.15 (24 trials, n=not reported)  SJC: SMD= −0.23, 95% CI: −0.42 to −0.04 (20 trials, n=not reported)  DMS: SMD= −0.40, 95% CI: −0.62 to -0.18 (20 trials, n=not reported)  Pain (VAS): SMD= −0.53, 95% CI: −1.03 to −0.03 (26 trials, n=not reported)  DAS28: SMD= −0.48, 95% CI: −0.80 to −0.17 (10 trials, n=not reported)  CRP: NS (20 trials, n=not reported)  ESR: SMD= −0.21, 95% CI: −0.39 to −0.04 (18 trials, n=not reported) |
| Gwinnutt 2022^230^  Umbrella review | Collagen | | Any control | Functional status: NS (1 trial, n=not reported; very low certainty of evidence; rationale not provided)+ | Pain: NS  Disease Activity: NS  TJC, SJC: NS  (1 trial, n=not reported) |
|  | Mussel extracts | | Any control | Functional status: NS (1 trial, n=not reported; very low certainty of evidence; rationale not provided)+ | Pain: NS (1 trial, n=not reported)  Disease activity: SMD= −0.94, 95% CI: −1.58 to −0.29 (1 trial, n=not reported)  TJC: 2 trials, no meta-analysis performed. 1 trial was significant (SMD= −0.79, 95% CI: −1.43 to −0.16), the other NS.  SJC: NS (2 trials, n= not reported)  DMS: NS (1 trial, n=not reported)  CRP: NS (2 trials, n= not reported)  ESR: NS (2 trials, n=not reported) |
|  | Creatine | | Placebo | Functional status: NS (1 trial, n=not reported; very low certainty of evidence; rationale not provided)+ | Disease activity: NS (1 trial, n=not reported) |
|  | Potassium | | Placebo |  | Disease activity: SMD= −2.98, 95% CI: −3.94 to −2.02 (1 trial, n=not reported; very low certainty evidence; rationale not provided)+  Pain: SMD= −2.63, 95% CI: −3.54 to −1.73  TJC: SMD= −2.20, 95% CI: −3.03 to −1.36  SJC: SMD= −2.76, 95% CI: −3.69 to −1.84  CRP: SMD= −0.80, 95% CI: −1.48 to −0.12  ESR: SMD= −1.93, 95% CI: −2.73 to −1.13  (1 trial, n=not reported) |
|  | Vitamin B6 | | Any control |  | Disease activity: NS (1 trial, n=not reported; very low certainty evidence; rationale not provided)+  TJC, SJC: NS  CRP: NS  (1 trial, n=not reported) |
|  | Microalgae oil | | Placebo | Functional status: NS (1 trial, n=not reported; very low certainty evidence; rationale not provided)+ | Disease activity: NS  TJC, SJC: NS  DMS: NS  CRP, ESR: NS  (1 trial, n=not reported) |
|  | Beta-hydroxy -beta- methylbutyrate + glutamine + arginine combination | | Placebo | Functional status: NS (1 trial, n=not reported; low certainty of evidence; rationale not provided)+ | Disease activity: SMD= −0.69, 95% CI: −1.33 to −0.05  ESR: NS  (1 trial, n=not reported) |
|  | Alpha-lipoic acid | | Placebo | Functional status NS (1 trial, n=not reported; low certainty evidence; rationale not provided) | Pain: NS (1 trial, n=not reported)  CRP: NS (2 trials, n=not reported) |
|  | Glucosamine | | Placebo |  | CRP, ESR: NS (1 trial, n=not repoted) |
|  | Linoleic acid | | Placebo |  | CRP, ESR: NS (1 trial, n=not repoted) |
|  | Quercetin | | Placebo | Functional status: SMD= −0.68, 95% CI: −1.18 to −0.18 (2 trials, n=not reported; low certainty of evidence; rationale not provided)+ | Pain: NS (2 trials, n=not reported)  Disease activity: NS (1 trial, n=not reported)  TJC, SJC: NS (1 trial, n=not reported)  CRP: NS (2 trials, n=not reported)  ESR: NS (2 trials n=not reported) |
|  | Antioxidants | | Placebo | Functional status: SMD= −0.86, 95% CI: −1.44 to −0.28 (1 trial, n=not reported; low certainty of evidence; rationale not provided)+ | Pain: SMD= −1.17, 95% CI: −1.77 to −0.57  TJC, SJC: NS  ESR: NS  (1 trial, n=not reported)  Disease activity: 2 trials, no meta-analysis performed, one trial significant, the other NS |
| MIND-BODY THERAPIES | | | | | |
| Ye, X 2020^266^  Meta-analysis | Yoga | | Any non-yoga intervention | Functional status (HAQ-DI):  SMD= −0.32, 95% CI: −0.58 to −0.05 (5 trials, n=525; low certainty evidence; downgraded for RoB and inconsistency) | Pain: NS (4 trials, n=517)  DAS28: SMD= −0.39, 95% CI: −0.68 to −0.10 (4 trials, n=342)  TJC: NS (2 trials, n= 72)  SJC: NS (2 trials, n= 72)  ESR: NS (2 trials, n=119)  CRP: NS (2 trials, n=152) |
|  | Yoga | | Usual care | Functional status (HAQ-DI): NS (2 trials, n=98; very low certainty evidence; downgraded for RoB, inconsistency and twice for imprecision) | DAS28: NS (2 trials, n=152) |
|  | Yoga + Medication | | Medication | Functional status (HAQ-DI): SMD= −0.58, 95% CI: −1.09 to -0.07 (2 trials, n=63; very low certainty evidence; downgraded for RoB and twice for imprecision) | Pain: NS (2 trials, n=73)  DAS28: SMD= −0.59, 95% CI: −0.88 to −0.30 (2 trials, n= 190) |
| Zhou, B 2020 ^273^  Meta-analysis | Mindfulness | | Routine nursing |  | Disease activity (DAS28): MD= −0.29, 95% CI: −0.38 to −0.19 (2 trials, n=166; very low certainty; downgraded twice for RoB, once for imprecision)  Pain (VAS): MD= −0.65, 95% CI: −1.11 to −0.18 (4 trials, n=300)  CRP: NS (2 trials, n=129) |
| Mudano, A 2019^248^  Meta-analysis | Tai Chi | | No Tai Chi | Functional status (HAQ): MD= −0.33, 95% CI: −0.79 to 0.12 (2 trials, n=63, very low certainty evidence; downgraded twice for imprecision [low number of participants, 95%CI includes null value] and study limitations [high withdrawal rate, lack of blinding])+ | Pain: NS (1 trial, n=20)  ACR20: NS (1 trial, n=20)  SJC: NS (1 trial, n=20)  TJC: NS (1 trial, n=20)  Withdrawals: NS (1 trial, n=46) |

ACR20= American College of Rheumatology outcome measures, AE= adverse effects, CI= confidence Interval, CRP= C-reactive protein, DAS-28= disease activity score, DMARD= disease-modifying anti-rheumatic drug, DMS= daily morning stiffness, ESR= erythrocyte sedimentation rate, HAQ= health assessment questionnaire, McGill = refers to the short form McGill Pain Questionnaire, MD= mean difference, NS= not significant, NSAID= nonsteroidal anti-inflammatory drug, OR= odds ratio, RaQOL=rheumatoid arthritis quality of life questionnaire, RAI = Ritchie Articular Index, RF= rheumatoid factor, RoB= risk of bias, RR= risk ratio, SJC= swollen joint count, SMD = standardized mean difference, TJC= tender joint count, TwHF= Tripterygium Wilfordii Hook F, WM= western medicine, VAS= visual analogue scale

## Supplementary Table 5 - Findings from studies on spondyloarthritis

| **Lead Author, Year Published, Study Design** | **Intervention** | **Control** | **Primary Outcome Findings**  **(+ = GRADE performed by original author)**  **(* = GRADE unable to be completed)** | **Secondary outcome findings**  **(+ = GRADE performed by original author)**  **(* = GRADE unable to be completed)** |
| --- | --- | --- | --- | --- |
| EXERCISE | | | | |
| Pecourneau, V 2018^252^  Meta-analysis | Exercise | Any control (usual care, physical therapy, education) | Functional status (BASFI): MD= −0.72, 95% CI: −1.03 to −0.40 (8 trials, n=not reported; very low certainty of evidence; downgraded twice for RoB, once for imprecision) | Disease activity (BASDAI): MD= −0.90, 95% CI: −1.52 to −0.27 (6 trials, n=not reported) |
| Ortolan, A 2023^251^  Systematic Review | Exercise | Any | Functional status – Moderate or high effect sizes (range in RCTs of exercise for BASFI was 0.04-0.92) | Disease activity and pain - moderate or high effect sizes (range in RCTs of exercise for ASDAS: 0.29–0.94, BASDAI: 0.14–1.43). |
| Regnaux, J 2020^254^  Meta-analysis | Any exercise | No intervention | Functional status (BASFI): MD= −1.3, 95% CI: −1.7 to −0.9 (7 trials, n=312; moderate certainty evidence; downgraded for risk of detection bias/lack of blinding of participants)+  Quality of life (ASQoL): NS (2 trials, n=85) | Pain (VAS): MD= −2.1, 95% CI: −3.6 to −0.6 (6 trials, n=288)  CRP: NS (2 trials, n=84)  ESR: MD= −5.36, 95% CI: −10.31 to −0.41 (2 trials, n=84)  AE: 1 AE was reported in 67 exercise participants, none in 43 control participants (2 trials, n=110) |
|  |  | Usual care | Functional status (BASFI): MD=−0.4, 95% CI: −0.6 to −0.2 (5 trials, n=1068; moderate certainty evidence; downgraded for risk of detection bias/lack of blinding of participants)+  Quality of life (ASQoL): NS (2 trials, n=809) | Pain (VAS):MD= −0.5, 95% CI: −0.9 to −0.1 (2 trials, n=911)  AE: 1 AE was reported in 67 exercise participants, none in 43 control participants (2 trials, n=110) |
| Bjork, M 2022 ^222^  Meta-analysis | Physical activity or exercise | Active (alternative exercise or physical activity) | For spondyloarthritis:  Activity performance: NS (5 trials, n=not reported; very low certainty evidence; downgraded one level each for inconsistency, imprecision and publication bias detected on funnel plot)  QoL: NS (1 trial, n=not reported)* | For spondyloarthritis:  Pain: NS (1 trial, n=not reported)  Disease activity: NS (1 trial, n=not reported) |
|  | Physical activity or exercise | Inactive (no treatment, waiting list, usual care) | For spondyloarthritis:  Activity performance: SMD= −0.60, 95% CI: −1.09 to −0.12 (4 trials, n=not reported; low certainty evidence; downgraded one level each for inconsistency and imprecision)  For psoriatic arthritis:  Activity performance: SMD= −0.65, 95% CI: −1.28 to −0.02 (1 trial, n=not reported)*  QoL: NS (1 trial, n=not reported)* | For spondyloarthritis:  Pain: SMD= −0.74, 95% CI: −1.02 to −0.45 (1 trial, n=not reported)  Disease activity: SMD= −0.67, 95% CI: −0.91 to −0.42 (3 trials, n=not reported) |
| Liang, H 2015^243^  Meta-analysis | Exercise + TNF inhibitor | TNF inhibitor | Functional status (BASFI): No meta-analysis was performed of the RCTs alone. However, none of the trials demonstrated significant findings. (3 trials, n=136)* | BASDAI: No meta-analysis was performed of the RCTs alone. However, neither trial demonstrated significant findings. (2 trials, n=76) |
| Hagen, K 2012^232^  Umbrella review | Home-based exercise | No intervention | Functional status: NS (1 trial reported in 1 review, n=155)* | Pain: SMD= 0.49, 95% CI: 0.17 to 0.81 (1 review, n=155) |
| Liang, H 2015^242^  Meta-analysis | Home-based exercise | Other exercise types, medical therapy | Functional status (BASFI):  MD= −0.39, 95% CI: −0.57 to −0.20 (6 trials, n=1098, very low certainty evidence; downgraded for inconsistency, imprecision and publication bias) | BASDAI: MD= −0.50, 95% CI: −0.99 to −0.02 (6 trials, n=1098)  Pain (VAS): NS (2 trials, n=822) |
| Gwinnutt, J 2022^231^  Umbrella review | Aerobic exercise | Any control | Functional status: NS (4 trials, n=not reported, low certainty evidence; rationale not provided)+  QoL: NS (2 trials, n=not reported)* | Pain: NS (2 trials, n=not reported)  Disease activity: NS (4 trials, n=not reported)  CRP: NS (2 trials, n=not reported)  ESR: NS (1 trial, n=not reported) |
|  | Combined aerobic and strength exercise | Any control | Functional status:  5 significant results from reviews  SMD= −0.72, 95% CI: 1.03 to −0.40  SMD= −0.39, 95% CI: −0.58 to −0.18  SMD =−0.51, 95% CI: −0.81 to −0.21  MD= −0.39, 95% CI: −0.57 to −0.20  From bespoke MA of trials: SMD= −0.87, 95% CI: −1.58 to −0.16 (6 meta-analyses, 4 trials, n=not reported, high certainty evidence; rationale not provided)+  QoL: NS (1 trial, n=not reported)* | Pain:  One significant result from reviews SMD= −0.42, 95% CI: −0.74 to −0.09, the other NS. (2 reviews, n=not reported)  One significant result from trials  SMD= −0.90, 95% CI: −1.32 to −0.48 (5 trials, no meta-analysis, n=not reported)  Disease activity: 5 significant results from reviews  SMD= −0.90, 95% CI: −1.52 to −0.27  SMD= −0.47, 95% CI: −0.84 to −0.09  MD= −0.50, 95% CI: −0.99 to −0.02  MD= −0.58, 95% CI: −1.10 to −0.06  SMD= −0.58, 95% CI: −0.94 to −0.22  (6 meta-analyses, n=not reported)  Significant result from trials: SMD= −0.56, 95% CI: −1.09 to −0.02 (3 trials, n=not reported)  CRP: NS (2 trials, n=not reported) |
|  | Aquatic | Land exercise/other exercises | Functional status: NS (2 trials, n=not reported; low certainty evidence; rationale not provided)+ | Pain: NS (2 trials, n=not reported)  Disease activity: NS (2 trials, n=not reported) |
|  | Strength | Any control | Functional status: SMD= −0.36, 95% CI: −0.60 to −0.13 (6 trials, n=not reported; moderate certainty evidence; rationale not provided)+  QoL: NS (2 trials, n=not reported)* | Pain: SMD -1.88 (-2.59, -1.17) (2 trials, n=not reported)  Disease activity: SMD = -0.37 (-0.72, -0.02) (6 trials, n=not reported)  CRP: NS (2 trials, n=not reported)  ESR: NS (2 trials, n=not reported) |
| Byrnes, K 2017^223^  Systematic Review | Pilates | Placebo or alternative exercise/ treatment | Functional status (BASFI): 2/2 trials showed significant improvement from either Pilates or a combined exercise program including Pilates compared to control. | 1/1 trial showed significant improvements in pain and BASDAI. |
| DIET | | | | |
| Ortolan, A 2023^250^  Systematic Review (for psoriatic arthritis) | Hypocaloric diet | Self-managed diet |  | For psoriatic arthritis:  Minimal Disease Activity (MDA) at 6-months: 1/1 trial showed that hypocaloric diet was significant in achievement of minimal disease activity.  HR= 1.85, 95% CI: 1.02 to 3.34  “The frequency of MDA achievement increased with increasing weight loss categories.” |
| NUTRIENT SUPPLEMENTS | | | | |
| Zeng, L 2022^268^  Meta-analysis | Probiotics | Placebo | Functional status BASFI: NS (1 trial, n=not reported)*  Only one trial | BASDAI: NS (1 trial, n=not reported)  AE: NS (2 trials, n=not reported) |
| Ortolan A et al 2023^250^  Systematic review | Omega-3 fatty acids (high-dose) | Low-dose omega-3 fatty acids |  | For ankylosing spondylitis:  Disease activity (BASDAI): 1/1 trials (n=24) noted a significant decrease in the high-dose group to the low-dose group. * |
| Gwinnutt 2022^230^  Umbrella review | Marine animal  omega-3 | Any control | For psoriatic arthritis:  Functional status NS (1 trial, n=not reported; low certainty evidence; rationale not provided)+ | For psoriatic arthritis:  Pain: NS (1 trial, n=not reported)  TJC: NS (2 trials, n=not reported))  SJC: SMD= −0.67, 95% CI: −1.31 to −0.03 (1 trial, n=not reported)  ESR: NS (1 trial, n=not reported)  CRP: NS (1 trial, n=not reported) |
|  | Selenium, coenzymeQ10, Vitamin E combination | Placebo |  | For psoriatic arthritis:  Disease severity: SMD= −8.03, 95% CI: −10.25 to −5.81 (1 trial, n=not reported; very low certainty evidence; rationale not provided)+ |
|  | Alpha-linoleic acid/linoleic acid/ /PUFAs/protein | Placebo |  | For spondyloarthritis  Disease activity: NS (1 review, n=not reported; very low certainty evidence; rationale not provided) |
|  | Long chain fatty acids | Placebo |  | For spondyloarthritis  Disease activity: NS (1 review, n=not reported; very low certainty evidence; rationale not provided)  ESR: "Significant association” (1 review, n=not reported) |

AE= Adverse Effects, ASQoL = Ankylosing Spondylitis Quality of Life, BASDAI= Bath Ankylosing Spondylitis Disease Activity Index, BASFI= Bath Ankylosing Spondylitis Functional Index, CI= confidence interval, CRP= C-reactive protein, DAS-28= disease activity score, DMARD= disease-modifying anti-rheumatic drug, ESR= erythrocyte sedimentation rate, HAQ= health assessment questionnaire, HR= hazard ratio, MD= mean difference, NS= Not significant, QoL = quality of life, RCT = randomized controlled trial, RoB= risk of bias, RR= risk ratio, SJC= swollen joint count, SMD = standardized mean difference, TJC= tender joint count, TNF= tumor necrosis factor VAS= visual analogue scale

## Supplementary Table 6 - Findings from studies on gout

| **Lead Author, Year Published, Study Design** | **Intervention** | **Control** | **Primary Outcome Findings**  **(+ = GRADE performed by original author)**  **(* = GRADE unable to be completed)** | **Secondary outcome findings**  **(+ = GRADE performed by original author)**  **(* = GRADE unable to be completed)** |
| --- | --- | --- | --- | --- |
| ACUPUNCTURE | | | | |
| Lu, W 2016^246^  Meta-analysis | Acupuncture therapy (manual/electro) as stand-alone or combination therapy | Western Medicine |  | Disease activity (sUA reduction):  MD = 41.30, 95% CI: 24.86 to 57.74 (22 trials, n=1910; very low certainty evidence; downgraded twice for RoB, once for inconsistency)  Pain (VAS): MD= 1.92, 95% CI: 0.96 to 2.87 (7 trials, n=551)  ESR reduction: MD= 1.75, 95% CI: 0.11 to 3.38 (5 trials, n=588)  CRP reduction: NS (4 trials, n=428)  AE: OR= 0.08, 95% CI: 0.03 to 0.23 (10 trials, n=807) |
| DIET | | | | |
| FitzGerald, J 2020 ^226^  Meta-analysis | Limited purine diet | No purine restriction |  | Gout flares – 6 months: NS (1 trial, n=29)*  Disease activity (sUA reduction):  NS (1 trial, n=29; very low certainty evidence; serious risk of bias, indirectness, imprecision)+ |
|  | Dairy protein | No increase in dairy protein intake |  | Gout flares – 3 months: NS (1 trial, n=80, moderate certainty evidence; downgraded for imprecision)  Gout flares – 6 months: NS (1 trial, n=29, very low certainty evidence; downgraded once for RoB, indirectness, twice for imprecision)  Disease activity (sUA reduction): NS (3 trials, n=134; moderate certainty evidence; serious imprecision)+  Pain: NS (1 trial, n=80)  AE: NS (1 trial, n=80) |
| CHINESE HERBAL MEDICINES | | | | |
| Zhou, L 2014^272^  Meta-analysis | Chinese herbal decoction | Western medicine |  | Disease activity (sUA reduction): SMD= 0.35, 95% CI: 0.03 to 0.67 (8 trials, n=519; very low certainty evidence; downgraded twice for RoB, inconsistency, once for indirectness, imprecision, publication bias)  CRP: NS (3 trials, n=181)  ESR: NS (5 trials, n=290)  AE: RR= 0.06, 95% CI: 0.03 to 0.13 (7 trials, n=507) |
| Li, X 2013^238^  Meta-analysis | Chinese herbal medicine +/- Western medicine | Western medicine |  | Disease activity (sUA reduction): MD= −50.10, 95% CI: −54.37 to −45.83 (40 trials, n=2975; moderate certainty evidence; randomisation is not clearly reported)+  Pain control: NS (12 trials, n=885)  CRP: MD= −1.02, 95% CI: −1.28 to −0.76 (13 trials, n=972)  AE: RR= 0.11, 95% CI: 0.08 to 0.15 (37 trials, n=not reported) |
| Zhang, Q 2020^270^  Meta-analysis | Guizhi-Shaoyao-Zhimu decoction alone | Conventional medicine |  | Disease activity (sUA reduction): MD = −54.06, 95% CI: −69.95 to −38.17 (7 trials, n=593; very low certainty evidence; downgraded twice for RoB, once for inconsistency, indirectness and imprecision)  ESR: SMD= −0.30, 95% CI: −0.56 to −0.03 (5 trials, n=348)  CRP: MD= −1.63, 95% CI: −2.36 to −0.91 (5 trials, n=348)  AE: RR= 0.15, 95% CI: 0.03 to 0.68 (3 trials, n=300) |
|  | Guizhi-Shaoyao-Zhimu decoction + conventional medicine | Conventional medicine |  | Disease activity (sUA reduction): MD= 36.28, 95% CI: 52.58 to 19.98 (4 trials, n=293; very low certainty evidence; downgraded for RoB, inconsistency, indirectness, imprecision)  ESR: SMD= −0.78, 95% CI: −1.35 to −0.21 (4 trials, n=313)  CRP: MD= −1.63, 95% CI: −1.88 to −1.37 (4 trials, n=313) |
| OTHER HERBAL MEDICINES | | | | |
| Zeng, L 2022^269^  Meta-analysis | Curcumin | Placebo |  | Disease activity (sUA reduction): NS (1 trial, n=not reported)*  AE: NS (1 trial, n=not reported) |
| FitzGerald, J 2020^226^  Meta-analysis | Cherry extract | No supplementation |  | Gout flares 6 months: NS (1 trial, n=29;very low certainty evidence; serious RoB, indirectness, imprecision)  Disease activity (sUA reduction): NS (1 trial, n=29; very low certainty evidence; serious risk of bias, indirectness, imprecision)+ |
| NUTRIENT SUPPLEMENTS | | | | |
| FitzGerald, J 2020^226^  Meta-analysis | Vitamin C | No supplementation/ allopurinol |  | Gout flares: NS (1 trial, n=29, low certainty evidence)+  sUA reduction: clinically insignificant changes at 2 and 6 months (2 trial, n=69; very low certainty evidence; serious risk of bias, indirectness, imprecision)+ |

AE= Adverse Effects, CI= Confidence Interval, CRP= C-reactive protein, DAS-28= disease activity score, DMARD= disease-modifying anti-rheumatic drug, ESR= erythrocyte sedimentation rate, HAQ= health assessment questionnaire, MD= mean difference, NS= Not significant, QoL= quality of life, RoB= risk of bias, RR= risk ratio, SJC= swollen joint count, SMD = standardized mean difference, sUA = serum uric acid, TJC= tender joint count, VAS= visual analogue scale.

Reference List

1. Abdulrazaq M, Innes JK, Calder PC. Effect of omega-3 polyunsaturated fatty acids on arthritic pain: A systematic review. Nutrition. 2017;39-40:57-66.

2. Ahc M. Dietary Influences on Rheumatoid Disease. Integrative Medicine Alert. 2021;24(7):1-4.

3. Akram A, Georgiou P, Shi W, Proute MC, Serhiyenia T, Pradeep R, et al. Impact of Change in Lifestyle and Exercise on Cognitive Function in Patients With Rheumatoid Arthritis: A Systematic Review. Cureus. 2021;13(9):e18268.

4. Al-Qubaeissy KY, Fatoye FA, Goodwin PC, Yohannes AM. The effectiveness of hydrotherapy in the management of rheumatoid arthritis: a systematic review. Musculoskeletal Care. 2013;11(1):3-18.

5. Alexanderson H, Bostrom C. Exercise therapy in patients with idiopathic inflammatory myopathies and systemic lupus erythematosus - A systematic literature review. Best Practice & Research in Clinical Rheumatology. 2020;34(2):101547.

6. Andrés M, Sivera F, Buchbinder R, Pardo Pardo J, Carmona L. Dietary supplements for chronic gout. Cochrane Database of Systematic Reviews. 2021(11).

7. Antico A, Tampoia M, Tozzoli R, Bizzaro N. Can supplementation with vitamin D reduce the risk or modify the course of autoimmune diseases? A systematic review of the literature. Autoimmunity Reviews. 2012;12(2):127-36.

8. Aqaeinezhad Rudbane SM, Rahmdel S, Abdollahzadeh SM, Zare M, Bazrafshan A, Mazloomi SM. The efficacy of probiotic supplementation in rheumatoid arthritis: a meta-analysis of randomized, controlled trials. Inflammopharmacology. 2018;26(1):67-76.

9. Bagherniya M, Darand M, Askari G, Guest PC, Sathyapalan T, Sahebkar A. The Clinical Use of Curcumin for the Treatment of Rheumatoid Arthritis: A Systematic Review of Clinical Trials. Adv Exp Med Biol. 2021;1291:251-63.

10. Baillet A, Vaillant M, Guinot M, Juvin R, Gaudin P. Efficacy of resistance exercises in rheumatoid arthritis: meta-analysis of randomized controlled trials. Rheumatology. 2012;51(3):519-27.

11. Balchin C, Tan AL, Golding J, Bissell LA, Wilson OJ, McKenna J, et al. Acute effects of exercise on pain symptoms, clinical inflammatory markers and inflammatory cytokines in people with rheumatoid arthritis: a systematic literature review. Ther Adv Musculoskelet Dis. 2022;14:1759720x221114104.

12. Batterhan SI, Heywood S, Keating JL. Systematic review and meta-analysis comparing land and aquatic exercise for people with hip or knee arthritis on function, mobility, and other health outcomes...Reprinted with permission. Original source: Batterham SI, Heywood S, Keating JL. Systematic review and meta-analysis comparing land and aquatic exercise for people with hip or knee arthritis on function, mobility and other health outcomes. BMC Musculoskeletal Disorders. 2011; 12:123. Journal of Aquatic Physical Therapy. 2013;20(2):10-24.

13. Bawa FL, Mercer SW, Atherton RJ, Clague F, Keen A, Scott NW, et al. Does mindfulness improve outcomes in patients with chronic pain? Systematic review and meta-analysis. British Journal of General Practice. 2015;65(635):e387-400.

14. Bellan M, Andreoli L, Nerviani A, Piantoni S, Avanzi GC, Soddu D, et al. Is cholecalciferol a potential disease-modifying anti-rheumatic drug for the management of rheumatoid arthritis? Clinical and Experimental Rheumatology. 2020;38(2):343-9.

15. Bergstra SA, Murgia A, Te Velde AF, Caljouw SR. A systematic review into the effectiveness of hand exercise therapy in the treatment of rheumatoid arthritis. Clinical Rheumatology. 2014;33(11):1539-48.

16. Bird JK, Troesch B, Warnke I, Calder PC. The effect of long chain omega-3 polyunsaturated fatty acids on muscle mass and function in sarcopenia: A scoping systematic review and meta-analysis. Clinical Nutrition ESPEN. 2021;46:73-86.

17. Bloomfield HE, Koeller E, Greer N, MacDonald R, Kane R, Wilt TJ. Effects on Health Outcomes of a Mediterranean Diet With No Restriction on Fat Intake: A Systematic Review and Meta-analysis. Annals of Internal Medicine. 2016;165(7):491-500.

18. Bodur H, Yurdakul FG, Ataman S, Garip Y, Nas K, Fikriye Figen Ayhan FFA, et al. Turkish League Against Rheumatism Consensus Report: Recommendations For Management of Axial Spondyloarthritis. Archives of Rheumatology. 2018;33(1):1-16.

19. Boison D, Adinortey CA, Babanyinah GK, Quasie O, Agbeko R, Wiabo-Asabil GK, et al. Costus afer: A Systematic Review of Evidence-Based Data in support of Its Medicinal Relevance. Scientifica. 2019;2019:3732687.

20. Boniface G, Gandhi V, Norris M, Williamson E, Kirtley S, O'Connell NE. A systematic review exploring the evidence reported to underpin exercise dose in clinical trials of rheumatoid arthritis. Rheumatology. 2020;59(11):3147-57.

21. Boudjani R, Challal S, Semerano L, Sigaux J. Impact of different types of exercise programs on ankylosing spondylitis: a systematic review and meta-analysis. Disability and rehabilitation. 2022;45(24):1-12.

22. Burghardt RD, Kazim MA, Ruther W, Niemeier A, Strahl A. The impact of physical activity on serum levels of inflammatory markers in rheumatoid arthritis: a systematic literature review. Rheumatology International. 2019;39(5):793-804.

23. Bussing A, Ostermann T, Ludtke R, Michalsen A. Effects of yoga interventions on pain and pain-associated disability: a meta-analysis. Journal of Pain. 2012;13(1):1-9.

24. Cai G, Wang L, Fan D, Xin L, Liu L, Hu Y, et al. Vitamin D in ankylosing spondylitis: review and meta-analysis. Clinica Chimica Acta. 2015;438:316-22.

25. Cai X, Chen XM, Xia X, Bao K, Wang RR, Peng JH, et al. The bone-protecting efficiency of Chinese medicines compared with Western medicines in rheumatoid arthritis: A systematic review and meta-analysis of comparative studies. Frontiers in Pharmacology. 2018;9(AUG) (no pagination):914.

26. Calder PC. Omega-3 polyunsaturated fatty acids and inflammatory processes: nutrition or pharmacology? British Journal of Clinical Pharmacology. 2013;75(3):645-62.

27. Carneiro S, Palominos PE, Anti SMA, Assad RL, Goncalves RSG, Chiereghin A, et al. Brazilian Society of Rheumatology 2020 guidelines for psoriatic arthritis. Advances in Rheumatology. 2021;61(1):69.

28. Carsons SE, Vivino FB, Parke A, Carteron N, Sankar V, Brasington R, et al. Treatment Guidelines for Rheumatologic Manifestations of Sjogren's Syndrome: Use of Biologic Agents, Management of Fatigue, and Inflammatory Musculoskeletal Pain. Arthritis Care & Research. 2017;69(4):517-27.

29. Casagrande PO, Coimbra DR, de Souza LC, Andrade A. Effects of yoga on depressive symptoms, anxiety, sleep quality, and mood in patients with rheumatic diseases: systematic review and meta-analysis. Pm & R. 2022;20(7):20.

30. Charoenngam N. Vitamin D and Rheumatic Diseases: A Review of Clinical Evidence. International Journal of Molecular Sciences. 2021;22(19):01.

31. Chen L, Luo Z, Wang M, Cheng J, Li F, Lu H, et al. The Efficacy and Mechanism of Chinese Herbal Medicines in Lowering Serum Uric Acid Levels: A Systematic Review. Frontiers in Pharmacology. 2020;11 (no pagination):578318.

32. Chen PE, Liu CY, Chien WH, Chien CW, Tung TH. Effectiveness of Cherries in Reducing Uric Acid and Gout: A Systematic Review. Evidence-Based Complementary and Alternative Medicine. 2019;2019 (no pagination):9896757.

33. Chen XM, Huang RY, Huang QC, Chu YL, Yan JY. Systemic Review and Meta-Analysis of the Clinical Efficacy and Adverse Effects of Zhengqing Fengtongning Combined with Methotrexate in Rheumatoid Arthritis. Evidence-Based Complementary and Alternative Medicine. 2015;2015 (no pagination):910376.

34. Chen XM, Wu JQ, Huang QC, Zhang JY, Pen JH, Huang ZS, et al. Systematic review and meta-analysis of the efficacy and safety of biqi capsule in rheumatoid arthritis patients. Experimental and Therapeutic Medicine. 2018;15(6)(6):5221-30.

35. Choi TY, Ang L, Ku B, Jun JH, Lee MS. Evidence map of cupping therapy. Journal of Clinical Medicine. 2021;10(8) (no pagination)(8).

36. Cohen M. Rosehip - an evidence based herbal medicine for inflammation and arthritis. Australian Family Physician. 2012;41(7):495-8.

37. Cramer H, Lauche R, Langhorst J, Dobos G. Yoga for rheumatic diseases: a systematic review. Rheumatology. 2013;52(11):2025-30.

38. Cramp F, Berry J, Gardiner M, Smith F, Stephens D. Health behaviour change interventions for the promotion of physical activity in rheumatoid arthritis: a systematic review. Musculoskeletal Care. 2013;11(4):238-47.

39. Cramp F, Hewlett S, Almeida C, Kirwan JR, Choy EH, Chalder T, et al. Non-pharmacological interventions for fatigue in rheumatoid arthritis. Cochrane Database of Systematic Reviews. 2013(8):CD008322.

40. Crevelario De Melo R, Victoria Ribeiro AA, Luquine Jr CD, De Bortoli MC, Toma TS, Barreto JOM. Effectiveness and safety of yoga to treat chronic and acute pain: A rapid review of systematic reviews. BMJ Open. 2021;11(12) (no pagination).

41. Daien C, Czernichow S, Letarouilly JG, Nguyen Y, Sanchez P, Sigaux J, et al. Dietary recommendations of the French Society for Rheumatology for patients with chronic inflammatory rheumatic diseases. Joint, Bone, Spine: Revue du Rhumatisme. 2022;89(2):105319.

42. Daily JW, Yang M, Park S. Efficacy of Turmeric Extracts and Curcumin for Alleviating the Symptoms of Joint Arthritis: A Systematic Review and Meta-Analysis of Randomized Clinical Trials. Journal of Medicinal Food. 2016;19(8):717-29.

43. Demmelmaier I, Iversen MD. How Are Behavioral Theories Used in Interventions to Promote Physical Activity in Rheumatoid Arthritis? A Systematic Review. Arthritis Care & Research. 2018;70(2):185-96.

44. Deng KF, Li LH, Pan TZ, Ning H, Lu HL, Huang YX, et al. Meta-analysis and trial sequential analysis on blood uric acid and joint function in gouty arthritis treated with fire needling therapy in comparison with western medication<sup></sup>. World Journal of Acupuncture - Moxibustion. 2022;32(1):49-60.

45. Diao M, Peng J, Wang D, Wang H. Peripheral vitamin D levels in ankylosing spondylitis: A systematic review and meta-analysis. Frontiers in Medicine. 2022;9:972586.

46. DiRenzo D, Crespo-Bosque M, Gould N, Finan P, Nanavati J, Bingham CO, 3rd. Systematic Review and Meta-analysis: Mindfulness-Based Interventions for Rheumatoid Arthritis. Current Rheumatology Reports. 2018;20(12):75.

47. Djalilova DM, Schulz PS, Berger AM, Case AJ, Kupzyk KA, Ross AC. Impact of Yoga on Inflammatory Biomarkers: A Systematic Review. Biological Research for Nursing. 2019;21(2):198-209.

48. Dos Santos LP, Santo R, Ramis TR, Portes JKS, Chakr R, Xavier RM. The effects of resistance training with blood flow restriction on muscle strength, muscle hypertrophy and functionality in patients with osteoarthritis and rheumatoid arthritis: A systematic review with meta-analysis. PLoS ONE [Electronic Resource]. 2021;16(11):e0259574.

49. Ebrahimzadeh A, Abbasi F, Ebrahimzadeh A, Jibril AT, Milajerdi A. Effects of curcumin supplementation on inflammatory biomarkers in patients with Rheumatoid Arthritis and Ulcerative colitis: A systematic review and meta-analysis. Complementary Therapies in Medicine. 2021;61:N.PAG-N.PAG.

50. Elma O, Yilmaz ST, Deliens T, Coppieters I, Clarys P, Nijs J, et al. Do nutritional factors interact with chronic musculoskeletal pain? A systematic review. Journal of Clinical Medicine. 2020;9(3) (no pagination).

51. Feng ZT, Xu J, He GC, Cai SJ, Li J, Mei ZG. A systemic review and meta-analysis of the clinical efficacy and safety of total glucosides of peony combined with methotrexate in rheumatoid arthritis. Clinical Rheumatology. 2018;37(1):35-42.

52. Ford AR, Siegel M, Bagel J, Cordoro KM, Garg A, Gottlieb A, et al. Dietary Recommendations for Adults With Psoriasis or Psoriatic Arthritis From the Medical Board of the National Psoriasis Foundation: A Systematic Review. JAMA Dermatology. 2018;154(8):934-50.

53. Forsyth C, Kouvari M, D'Cunha NM, Georgousopoulou EN, Panagiotakos DB, Mellor DD, et al. The effects of the Mediterranean diet on rheumatoid arthritis prevention and treatment: a systematic review of human prospective studies. Rheumatology International. 2018;38(5):737-47.

54. Foulkes AC, Ferguson F, Grindlay DJC, Williams HC, Griffiths CEM, Warren RB. What's new in psoriasis treatment? An analysis of systematic reviews published in 2015. Clinical & Experimental Dermatology. 2018;43(7):759-65.

55. Fraenkel L, Bathon JM, England BR, St Clair EW, Arayssi T, Carandang K, et al. 2021 American College of Rheumatology Guideline for the Treatment of Rheumatoid Arthritis. Arthritis & Rheumatology. 2021;73(7):1108-23.

56. Franco AS, Freitas TQ, Bernardo WM, Pereira RMR. Vitamin D supplementation and disease activity in patients with immune-mediated rheumatic diseases: A systematic review and meta-analysis. Medicine. 2017;96(23):e7024.

57. Gandhi GR, Jothi G, Mohana T, Vasconcelos ABS, Montalvao MM, Hariharan G, et al. Anti-inflammatory natural products as potential therapeutic agents of rheumatoid arthritis: A systematic review. Phytomedicine. 2021;93:153766.

58. Garza V. Unconventional Pain Management for People With Systemic Lupus Erythematosus: A Systematic Literature Review. Journal of Applied Rehabilitation Counseling. 2020;51(1):3-15.

59. Geenen R, Overman CL, Christensen R, Asenlof P, Capela S, Huisinga KL, et al. EULAR recommendations for the health professional's approach to pain management in inflammatory arthritis and osteoarthritis. Annals of the Rheumatic Diseases. 2018;77(6):797-807.

60. Geneen LJ, Moore RA, Clarke C, Martin D, Colvin LA, Smith BH. Physical activity and exercise for chronic pain in adults: an overview of Cochrane Reviews. Cochrane Database of Systematic Reviews. 2017;4(4):CD011279.

61. Genel F, Kale M, Pavlovic N, Flood VM, Naylor JM, Adie S. Health effects of a low-inflammatory diet in adults with arthritis: a systematic review and meta-analysis. Journal of Nutritional Science. 2020;9:e37.

62. Gilbey A, Perezgonzalez JD. Health benefits of deer and elk velvet antler supplements: a systematic review of randomised controlled studies. New Zealand Medical Journal. 2012;125(1367):80-6.

63. Gilbey A, Ernst E, Tani K. A systematic review of reviews of systematic reviews of acupuncture. Focus on Alternative & Complementary Therapies. 2013;18(1):8-18.

64. Gillani SW, Abdul MIM, Zaghloul HA, Ansari IA, Ata-Ur-Rahman S, Farooqui S. Management of diabetes and arthritis -A systematic review. Tropical Journal of Pharmaceutical Research. 2018;17(5):967-73.

65. Gioxari A, Kaliora AC, Marantidou F, Panagiotakos DP. Intake of omega-3 polyunsaturated fatty acids in patients with rheumatoid arthritis: A systematic review and meta-analysis. Nutrition. 2018;45:114-24.e4.

66. Gualdron AJ. Alternative medicine as treatment of osteoarthritis and rheumatoid arthritis. Systematic review of literature and meta-analysis. Revista Colombiana de Reumatologia. 2012;19(4):234-44.

67. Gwinnutt JM, Wieczorek M, Balanescu A, Bischoff-Ferrari HA, Boonen A, Cavalli G, et al. 2021 EULAR recommendations regarding lifestyle behaviours and work participation to prevent progression of rheumatic and musculoskeletal diseases. Annals of the Rheumatic Diseases. 2022;08(1):08.

68. Hall A, Copsey B, Richmond H, Thompson J, Ferreira M, Latimer J, et al. Effectiveness of Tai Chi for Chronic Musculoskeletal Pain Conditions: Updated Systematic Review and Meta-Analysis. Physical Therapy. 2017;97(2):227-38.

69. Hammond A, Prior Y. The effectiveness of home hand exercise programmes in rheumatoid arthritis: a systematic review. British Medical Bulletin. 2016;119(1):49-62.

70. Harpham C, Harpham QK, Barker AR. The effect of exercise training programs with aerobic components on C-reactive protein, erythrocyte sedimentation rate and self-assessed disease activity in people with ankylosing spondylitis: A systematic review and meta-analysis. International Journal of Rheumatic Diseases. 2022;25(6):635-49.

71. He Y, Li Y, Li J, Zhang Y, Li N. Analysis of the Most-Cited Systematic Review or Meta-Analysis in Acupuncture Research. Evidence-Based Complementary and Alternative Medicine. 2021;2021 (no pagination):3469122.

72. Hoving JL, Lacaille D, Urquhart DM, Hannu TJ, Sluiter JK, Frings‐Dresen MHW. Non‐pharmacological interventions for preventing job loss in workers with inflammatory arthritis. Cochrane Database of Systematic Reviews. 2014(11):CD010208.

73. Hu J, Mao Y, Zhang Y, Ye D, Wen C, Xie Z. Moxibustion for the treatment of ankylosing spondylitis: a systematic review and meta-analysis. Annals of Palliative Medicine. 2020;9(3):709-20.

74. Hu X, Chen J, Tang W, Chen W, Sang Y, Jia L. Effects of exercise programmes on pain, disease activity and function in ankylosing spondylitis: A meta-analysis of randomized controlled trials. European Journal of Clinical Investigation. 2020;50(12):e13352.

75. Huang X, Zhu Z, Wu G, Min P, Fei P, Zhu T, et al. Efficacy and safety of external application of Traditional Chinese Medicine for the treatment of acute gouty arthritis: a systematic review and Meta-analysis. Journal of Traditional Chinese Medicine. 2019;39(3):297-306.

76. Huang Y, Wang H, Chen Z, Wang Y, Qin K, Huang Y, et al. Synergistic and Hepatoprotective Effect of Total Glucosides of Paeony on Ankylosing Spondylitis: A Systematic Review and Meta-Analysis. Frontiers in Pharmacology. 2019;10:231.

77. Huo X, Liang L, Ding X, Bihazi A, Xu H. Efficacy and Safety of Acupuncture with Western Medicine for Rheumatoid Arthritis: A Systematic Review and Meta-analysis. Acupuncture & Electro-Therapeutics Research. 2021;46(4):371-82.

78. Huston P, McFarlane B. Health benefits of tai chi: What is the evidence? Canadian Family Physician. 2016;62(11):881-90.

79. Ilchovska DD, Barrow DM. An Overview of the NF-kB mechanism of pathophysiology in rheumatoid arthritis, investigation of the NF-kB ligand RANKL and related nutritional interventions. Autoimmunity Reviews. 2021;20(2):102741.

80. Imoto AM, Amorim FF, Palma H, Lombardi Junior I, Salomon AL, Peccin MS, et al. Evidence for the efficacy of Tai Chi for treating rheumatoid arthritis: an overview of systematic reviews. Sao Paulo Medical Journal = Revista Paulista de Medicina. 2021;139(2):91-7.

81. Jabbari M, Barati M, Khodaei M, Babashahi M, Kalhori A, Tahmassian AH, et al. Is collagen supplementation friend or foe in rheumatoid arthritis and osteoarthritis? A comprehensive systematic review. International Journal of Rheumatic Diseases. 2022;25(9):973-81.

82. Katchamart W, Narongroeknawin P, Chevaisrakul P, Dechanuwong P, Mahakkanukrauh A, Kasitanon N, et al. Evidence-based recommendations for the diagnosis and management of rheumatoid arthritis for non-rheumatologists: Integrating systematic literature research and expert opinion of the Thai Rheumatism Association. International Journal of Rheumatic Diseases. 2017;20(9):1142-65.

83. Kazemi A, Soltani S, Ghorabi S, Keshtkar A, Daneshzad E, Nasri F, et al. Effect of probiotic and synbiotic supplementation on inflammatory markers in health and disease status: A systematic review and meta-analysis of clinical trials. Clinical Nutrition. 2020;39(3):789-819.

84. Kelley GA, Kelley KS, Hootman JM. Effects of exercise on depression in adults with arthritis: a systematic review with meta-analysis of randomized controlled trials. Arthritis Research & Therapy. 2015;17(1):21.

85. Kelley GA, Kelley KS. Exercise reduces depressive symptoms in adults with arthritis: Evidential value. World Journal of Rheumatology. 2016;6(2):23-9.

86. Kelley GA, Kelley KS, Callahan LF. Aerobic Exercise and Fatigue in Rheumatoid Arthritis Participants: A Meta-Analysis Using the Minimal Important Difference Approach. Arthritis Care & Research. 2018;70(12):1735-9.

87. Kelley GA, Kelley KS, Callahan LF. Community-deliverable exercise and anxiety in adults with arthritis and other rheumatic diseases: a systematic review with meta-analysis of randomised controlled trials. BMJ Open. 2018;8(2):e019138.

88. Kelley GA, Kelley KS. Effects of exercise on depressive symptoms in adults with arthritis and other rheumatic disease: a systematic review of meta-analyses. Journal of the Academy of Chiropractic Orthopedists. 2014;11(4):21-3.

89. Kessler J, Chouk M, Ruban T, Prati C, Wendling D, Verhoeven F. Psoriatic arthritis and physical activity: a systematic review. Clinical Rheumatology. 2021;40(11):4379-89.

90. Khabbazi A, Javadivala Z, Seyedsadjadi N, Malek Mahdavi A. A Systematic Review of the Potential Effects of Nigella sativa on Rheumatoid Arthritis. Planta Medica. 2020;86(7):457-69.

91. Klaps S, Haesevoets S, Verbunt J, Koke A, Janssens L, Timmermans A, et al. The Influence of Exercise Intensity on Psychosocial Outcomes in Musculoskeletal Disorders: A Systematic Review. Sports & Health. 2022;14(6):19417381221075354.

92. Ko SH, Chi CC, Yeh ML, Wang SH, Tsai YS, Hsu MY. Lifestyle changes for treating psoriasis. Cochrane Database of Systematic Reviews. 2019;7(7):CD011972.

93. Lane B, McCullagh R, Cardoso JR, McVeigh JG. The effectiveness of group and home‐based exercise on psychological status in people with ankylosing spondylitis: A systematic review and meta‐analysis. Musculoskeletal Care. 2022;20(4):758-71.

94. Lane B, McCullagh R, Cardoso JR, McVeigh JG. The effectiveness of group and home-based exercise on psychological status in people with ankylosing spondylitis: A systematic review and meta-analysis. Musculoskeletal Care. 2022;18(4):18.

95. Larkin L, Kennedy N. Correlates of physical activity in adults with rheumatoid arthritis: a systematic review. Journal of Physical Activity & Health. 2014;11(6):1248-61.

96. Lee JA, Son MJ, Choi J, Jun JH, Kim JI, Lee MS. Bee venom acupuncture for rheumatoid arthritis: a systematic review of randomised clinical trials. BMJ Open. 2014;4(11):e006140.

97. Lee MS, Ernst E. Systematic reviews of t'ai chi: an overview. British Journal of Sports Medicine. 2012;46(10):713-8.

98. Lee WB, Woo SH, Min BI, Cho SH. Acupuncture for gouty arthritis: a concise report of a systematic and meta-analysis approach. Rheumatology. 2013;52(7):1225-32.

99. Lee YH, Bae SC. Vitamin D level in rheumatoid arthritis and its correlation with the disease activity: a meta-analysis. Clinical & Experimental Rheumatology. 2016;34(5):827-33.

100. Lee YH, Bae SC, Song GG. Omega-3 polyunsaturated fatty acids and the treatment of rheumatoid arthritis: a meta-analysis. Archives of Medical Research. 2012;43(5):356-62.

101. Li H, Guo F, Luo YC, Zhu JP, Wang JL. Efficacy of tripterygium glycosides tablet in treating ankylosing spondylitis: a systematic review and meta-analysis of randomized controlled trials. Clinical Rheumatology. 2015;34(11):1831-8.

102. Li J, Yang J, Wu S, Wang MR, Zhu JM. Effects of acupuncture on rheumatoid arthritis: A systematic review and meta-analysis. African Journal of Traditional, Complementary and Alternative Medicines. 2016;13(2):61-71.

103. Li TP, Zhang AH, Miao JH, Sun H, Yan GL, Wu FF, et al. Applications and potential mechanisms of herbal medicines for rheumatoid arthritis treatment: a systematic review. RSC advances. 2019;9(45):26381-92.

104. Li X, Bi X, Wang S, Zhang Z, Li F, Zhao AZ. Therapeutic Potential of omega-3 Polyunsaturated Fatty Acids in Human Autoimmune Diseases. Frontiers in Immunology. 2019;10:2241.

105. Li Z, Chen M, Tang C. Improvement of Acupuncture Therapy on Relapse of Patients with Gouty Arthritis: A Pairwise and Bayesian Network Meta-analysis. Acupuncture & Electro-Therapeutics Research. 2022;47(2):167-82.

106. Liang H, Xu L, Tian X, Wang S, Liu X, Dai Y, et al. The comparative efficacy of supervised- versus home-based exercise programs in patients with ankylosing spondylitis: A meta-analysis. Medicine. 2020;99(8):e19229.

107. Liang H, Wu Y, Zhang W, Deng P, Huang FS, Du X, et al. Efficacy and Safety of Acupuncture Combined with Herbal Medicine in Treating Gouty Arthritis: Meta-Analysis of Randomized Controlled Trials. Evidence-Based Complementary & Alternative Medicine: eCAM. 2021;2021:8161731.

108. Lin J, Liu J, Davies ML, Chen W. Serum Vitamin D Level and Rheumatoid Arthritis Disease Activity: Review and Meta-Analysis. PLoS ONE [Electronic Resource]. 2016;11(1):e0146351.

109. Lin SS, Liu CX, Zhang JH, Wang H, Zhai JB, Mao JY, et al. Efficacy and Safety of Sinomenine Preparation for Ankylosing Spondylitis: A Systematic Review and Meta-Analysis of Clinical Randomized Controlled Trials. Evidence-Based Complementary and Alternative Medicine. 2020;2020 (no pagination):4593412.

110. Liu B, Meng X, Ma Y, Li H, Liu Y, Shi N, et al. Clinical safety of total glucosides of paeony adjuvant therapy for rheumatoid arthritis treatment: a systematic review and meta-analysis. BMC Complementary Medicine and Therapies. 2021;21(1):102.

111. Liu WH, Chen C, Wang F, Guo SN, Hao Y, Li SD. Development trend and current situation of acupuncture-moxibustion indications. World Journal of Acupuncture - Moxibustion. 2020;30(4):245-50.

112. Liu W, Qian X, Ji W, Lu Y, Wei G, Wang Y. Effects and safety of Sinomenine in treatment of rheumatoid arthritis contrast to methotrexate: a systematic review and Meta-analysis. Journal of Traditional Chinese Medicine. 2016;36(5):564-77.

113. Liu Y, Tu S, Gao W, Wang Y, Liu P, Hu Y, et al. Extracts of Tripterygium wilfordii Hook F in the Treatment of Rheumatoid Arthritis: A Systemic Review and Meta-Analysis of Randomised Controlled Trials. Evidence-Based Complementary & Alternative Medicine: eCAM. 2013;2013:410793.

114. Lowe JR, Briggs AM, Whittle S, Stephenson MD. A systematic review of the effects of probiotic administration in inflammatory arthritis. Complementary Therapies in Clinical Practice. 2020;40:N.PAG-N.PAG.

115. Ma SY, Wang Y, Xu JQ, Zheng L. Cupping therapy for treating ankylosing spondylitis: The evidence from systematic review and meta-analysis. Complementary Therapies in Clinical Practice. 2018;32:187-94.

116. Ma Y, Zhang X, Fan D, Xia Q, Wang M, Pan F. Common trace metals in rheumatoid arthritis: A systematic review and meta-analysis. Journal of Trace Elements in Medicine & Biology. 2019;56:81-9.

117. Macedo MS. Systematic review summary - Non-pharmacological interventions for fatigue in rheumatoid arthritis. Singapore Nursing Journal. 2014;41(2):48-50.

118. Macfarlane GJ, Paudyal P, Doherty M, Ernst E, Lewith G, MacPherson H, et al. A systematic review of evidence for the effectiveness of practitioner-based complementary and alternative therapies in the management of rheumatic diseases: rheumatoid arthritis. Rheumatology. 2012;51(9):1707-13.

119. Manara M, Bortoluzzi A, Favero M, Prevete I, Scire CA, Bianchi G, et al. Italian Society of Rheumatology recommendations for the management of gout. Reumatismo. 2013;65(1):4-21.

120. Marikar Bawa FL, Mercer SW, Atherton RJ, Clague F, Keen A, Scott NW, et al. Does mindfulness improve outcomes in patients with chronic pain? Systematic review and meta-analysis. British Journal of General Practice. 2015;65(635):e387-e400.

121. Marques A, Santos E, Nikiphorou E, Bosworth A, Carmona L. Effectiveness of self-management interventions in inflammatory arthritis: a systematic review informing the 2021 EULAR recommendations for the implementation of self-management strategies in patients with inflammatory arthritis. RMD Open. 2021;7(2):05.

122. Martins NA, Furtado GE, Campos MJ, Leitao JC, Filaire E, Ferreira JP. Exercise and ankylosing spondylitis with New York modified criteria: a systematic review of controlled trials with meta-analysis. Acta Reumatologica Portuguesa. 2014;39(4):298-308.

123. McKenna S, Donnelly A, Fraser A, Comber L, Kennedy N. Does exercise impact on sleep for people who have rheumatoid arthritis? A systematic review. Rheumatology International. 2017;37(6):963-74.

124. McKenna SG, Herring MP, Donnelly A, Comber L, Fraser A, Kennedy NM. Exercise Effects On Depressive and Anxiety Symptoms, Fatigue And Pain in Rheumatoid Arthritis: A Meta-Analysis: 1953 Board #214 May 31 3:30 PM - 5:00 PM...American College of Sports Medicine Annual Meeting, May 29-June 2, 2018, Minneapolis, Minnesota. Medicine & Science in Sports & Exercise. 2018;50(5S):471-2.

125. Medrado LN, Mendonca MLM, Budib MB, Oliveira-Junior SA, Martinez PF. Effectiveness of aquatic exercise in the treatment of inflammatory arthritis: systematic review. Rheumatology International. 2022;42(10):1681-91.

126. Miles EA, Calder PC. Influence of marine n-3 polyunsaturated fatty acids on immune function and a systematic review of their effects on clinical outcomes in rheumatoid arthritis. British Journal of Nutrition. 2012;107:S171-84.

127. Millner JR, Barron JS, Beinke KM, Butterworth RH, Chasle BE, Dutton LJ, et al. Exercise for ankylosing spondylitis: An evidence-based consensus statement. Seminars in Arthritis & Rheumatism. 2016;45(4):411-27.

128. Mohammed AT, Khattab M, Ahmed AM, Turk T, Sakr N, A MK, et al. The therapeutic effect of probiotics on rheumatoid arthritis: a systematic review and meta-analysis of randomized control trials. Clinical Rheumatology. 2017;36(12):2697-707.

129. Moi JH, Sriranganathan MK, Edwards CJ, Buchbinder R. Lifestyle interventions for acute gout. Cochrane Database of Systematic Reviews. 2013;2013(11):CD010519.

130. Moi JH, Sriranganathan MK, Falzon L, Edwards CJ, van der Heijde DM, Buchbinder R. Lifestyle interventions for the treatment of gout: a summary of 2 Cochrane systematic reviews. Journal of Rheumatology - Supplement. 2014;92:26-32.

131. Musumeci L, Maugeri A, Russo C, Lombardo GE, Cirmi S, Navarra M. Citrus Flavonoids and Autoimmune Diseases: A Systematic Review of Clinical Studies. Current Medicinal Chemistry. 2022;29(19):29.

132. Nagy G, Roodenrijs NMT, Welsing PMJ, Kedves M, Hamar A, van der Goes MC, et al. EULAR points to consider for the management of difficult-to-treat rheumatoid arthritis. Annals of the Rheumatic Diseases. 2022;81(1):20-33.

133. Nelson J, Sjoblom H, Gjertsson I, Ulven SM, Lindqvist HM, Barebring L. Do Interventions with Diet or Dietary Supplements Reduce the Disease Activity Score in Rheumatoid Arthritis? A Systematic Review of Randomized Controlled Trials. Nutrients. 2020;12(10):29.

134. Nelson NL, Churilla JR. Massage Therapy for Pain and Function in Patients With Arthritis: A Systematic Review of Randomized Controlled Trials. American Journal of Physical Medicine & Rehabilitation. 2017;36(5):665-72.

135. Ng JY, Azizudin AM. Rheumatoid arthritis and osteoarthritis clinical practice guidelines provide few complementary and alternative medicine therapy recommendations: a systematic review. Clinical Rheumatology. 2020;39(10):2861-73.

136. Nielsen SM, Zobbe K, Kristensen LE, Christensen R. Nutritional recommendations for gout: An update from clinical epidemiology. Autoimmunity Reviews. 2018;17(11):1090-6.

137. Nishishinya Aquino MB, Pereda CA, Munoz-Ortego J. Efficacy of acupuncture in rheumatic diseases with spine involvement: Systematic review. Revista Internacional de Acupuntura. 2019;13(2)(6):49-56.

138. O'Dwyer T, O'Shea F, Wilson F. Exercise therapy for spondyloarthritis: a systematic review. Rheumatology International. 2014;34(7):887-902.

139. O'Dwyer T, O'Shea F, Wilson F. Physical activity in spondyloarthritis: a systematic review. Rheumatology International. 2015;35(3):393-404.

140. Orla G, Bechet S, Walshe M. Modified Diet Use in Adults with Temporomandibular Disorders related to Rheumatoid Arthritis: A Systematic Review. Mediterranean Journal of Rheumatology. 2020;31(2):183-9.

141. Osthoff A-KR, Niedermann K, Braun J, Adams J, Brodin N, Dagfinrud H, et al. 2018 EULAR recommendations for physical activity in people with inflammatory arthritis and osteoarthritis. Annals of the Rheumatic Diseases. 2018;77(9):1251-60.

142. Palabindala V. 2017 - Guideline: In acute gout, steroids, NSAIDs, or low-dose colchicine are recommended; lifestyle changes are not supported. ACP Journal Club. 2017;166(4):1-.

143. Pan X, Lopez-Olivo MA, Nayak P, Suarez-Almazor ME. Quality Assessment of Controlled Trials Evaluating Chinese Herbal Medicine in Patients With Rheumatoid Arthritis: A Systematic Review. Value in Health. 2014;17(7):A547.

144. Pan X, Lopez-Olivo MA, Song J, Pratt G, Suarez-Almazor ME. Systematic review of the methodological quality of controlled trials evaluating Chinese herbal medicine in patients with rheumatoid arthritis. BMJ Open. 2017;7(3):e013242.

145. Parperis K, Papachristodoulou E, Kakoullis L, Rosenthal AK. Management of calcium pyrophosphate crystal deposition disease: A systematic review. Seminars in Arthritis & Rheumatism. 2021;51(1):84-94.

146. Parvova I, Hristov E, Rangelov A. ANalysis of adverse drug reactions in the treatment of rheumatological diseases with biological medicinal products- A systematic review of scientific publications. Rheumatology (Bulgaria). 2019;27(4):3-17.

147. Pei J-H, Ma T, Nan R-L, Chen H-X, Zhang Y-B, Gou L, et al. Mindfulness-Based Cognitive Therapy for Treating Chronic Pain A Systematic Review and Meta-analysis. Psychology, health & medicine. 2021;26(3):333-46.

148. Pereira L, Monteiro R. Tailoring gut microbiota with a combination of Vitamin K and probiotics as a possible adjuvant in the treatment of rheumatic arthritis: a systematic review. Clinical Nutrition ESPEN. 2022.

149. Peres D, Sagawa Y, Jr., Dugue B, Domenech SC, Tordi N, Prati C. The practice of physical activity and cryotherapy in rheumatoid arthritis: systematic review. European journal of physical & rehabilitation medicine. 2017;53(5):775-87.

150. Peter WF, Swart NM, Meerhoff GA, Vlieland TPMV. Clinical Practice Guideline for Physical Therapist Management of People With Rheumatoid Arthritis. PTJ: Physical Therapy & Rehabilitation Journal. 2021;101(8):1-16.

151. Phang JK, Kwan YH, Goh H, Tan VIC, Thumboo J, Ostbye T, et al. Complementary and alternative medicine for rheumatic diseases: A systematic review of randomized controlled trials. Complementary Therapies in Medicine. 2018;37:143-57.

152. Philippou E, Petersson SD, Erodotou S, Giallouri E, Rodomar C, Nikiphorou E. Dietary intake, dietary interventions, nutrient supplements and rheumatoid arthritis: systematic review of the evidence. Proceedings of the Nutrition Society. 2019;78(OCE1):1-.

153. Porras M, Rada G, Duran J. Effects of Mediterranean diet on the treatment of rheumatoid arthritis. Medwave. 2019;19(5):e7640.

154. Pourhabibi-Zarandi F, Shojaei-Zarghani S, Rafraf M. Curcumin and rheumatoid arthritis: A systematic review of literature. International Journal of Clinical Practice. 2021;75(10):e14280.

155. Qiu R, Zhang X, Zhao C, Li M, Shang H. Comparison of the efficacy of dispensing granules with traditional decoction: A systematic review and meta-analysis. Annals of Translational Medicine. 2018;6(3) (no pagination)(3):38.

156. Raad T, Griffin A, George ES, Larkin L, Fraser A, Kennedy N, et al. Dietary Interventions with or without Omega-3 Supplementation for the Management of Rheumatoid Arthritis: A Systematic Review. Nutrients. 2021;13(10):04.

157. Ramos A, Dominguez J, Gutierrez S. Acupuncture for rheumatoid arthritis. Medwave. 2018;18(6):e7284.

158. Rausch Osthoff AK, Juhl CB, Knittle K, Dagfinrud H, Hurkmans E, Braun J, et al. Effects of exercise and physical activity promotion: Meta-analysis informing the 2018 EULAR recommendations for physical activity in people with rheumatoid arthritis, spondyloarthritis and hip/knee osteoarthritis. RMD Open. 2018;4(2) (no pagination)(2):e000713.

159. Resende GG, Meirelles ES, Marques CDL, Chiereghin A, Lyrio AM, Ximenes AC, et al. The Brazilian Society of Rheumatology guidelines for axial spondyloarthritis - 2019. Advances in Rheumatology. 2020;60(1):19.

160. Rongen-van Dartel SA, Repping-Wuts H, Flendrie M, Bleijenberg G, Metsios GS, van den Hout WB, et al. Effect of Aerobic Exercise Training on Fatigue in Rheumatoid Arthritis: A Meta-Analysis. Arthritis Care & Research. 2015;67(8):1054-62.

161. Roodenrijs NMT, Hamar A, Kedves M, Nagy G, van Laar JM, van der Heijde D, et al. Pharmacological and non-pharmacological therapeutic strategies in difficult-to-treat rheumatoid arthritis: a systematic literature review informing the EULAR recommendations for the management of difficult-to-treat rheumatoid arthritis. RMD Open. 2021;7(1):01.

162. Sahebari M, Rezaieyazdi Z, Khodashahi M. Selenium and Autoimmune Diseases: A Review Article. Current Rheumatology Reviews. 2019;15(2):123-34.

163. Salmon VE, Hewlett S, Walsh NE, Kirwan JR, Cramp F. Physical activity interventions for fatigue in rheumatoid arthritis: a systematic review. Physical Therapy Reviews. 2017;22(1/2):12-22.

164. Sanchez P, Letarouilly JG, Nguyen Y, Sigaux J, Barnetche T, Czernichow S, et al. Efficacy of Probiotics in Rheumatoid Arthritis and Spondyloarthritis: A Systematic Review and Meta-Analysis of Randomized Controlled Trials. Nutrients. 2022;14(2):14.

165. Santos EJF, Duarte C, Ferreira RJO, Pinto AM, Moreira A, Vaz C, et al. Portuguese multidisciplinary recommendations for non-pharmacological and non-surgical interventions in patients with rheumatoid arthritis. Acta Reumatologica Portuguesa. 2021;46(1):40-54.

166. Santos EJF, Duarte C, Marques A, Cardoso D, Apóstolo J, da Silva JAP, et al. Effectiveness of non-pharmacological and non-surgical interventions for rheumatoid arthritis: An umbrella review. JBI Database of Systematic Reviews and Implementation Reports. 2019;17(7):1494-531.

167. Saracoglu I, Kurt G, Okur EO, Afsar E, Seyyar GK, Calik BB, et al. The effectiveness of specific exercise types on cardiopulmonary functions in patients with ankylosing spondylitis: a systematic review. Rheumatology International. 2017;37(3):409-21.

168. Sawant O, Khan T. Management of psoriatic arthritis: An overview of synthetic, recombinant dna, monoclonal antibody and nature-derived agents. International Journal of Pharmaceutical Sciences and Research. 2021;12(6):3090-103.

169. Seca S, Miranda D, Cardoso D, Nogueira B, Greten HJ, Cabrita A, et al. Effectiveness of Acupuncture on Pain, Physical Function and Health-Related Quality of Life in Patients with Rheumatoid Arthritis: A Systematic Review of Quantitative Evidence. Chinese Journal of Integrative Medicine. 2019;25(9):704-9.

170. Senftleber NK, Nielsen SM, Andersen JR, Bliddal H, Tarp S, Lauritzen L, et al. Marine Oil Supplements for Arthritis Pain: A Systematic Review and Meta-Analysis of Randomized Trials. Nutrients. 2017;9(1):06.

171. Sharma M. Yoga as an alternative and complementary approach for arthritis: a systematic review. Journal of Evidence-Based Complementary & Alternative Medicine. 2014;19(1):51-8.

172. Shekelle PG, Newberry SJ, FitzGerald JD, Motala A, O'Hanlon CE, Tariq A, et al. Management of Gout: A Systematic Review in Support of an American College of Physicians Clinical Practice Guideline. Annals of Internal Medicine. 2017;166(1):37-51.

173. Shen B, Sun Q, Chen H, Li Y, Du X, Li H, et al. Effects of moxibustion on pain behaviors in patients with rheumatoid arthritis: A meta-analysis. Medicine. 2019;98(30):e16413.

174. Sieczkowska SM, Casagrande PdO, Coimbra DR, Vilarino GT, Andreato LV, Andrade A. Effect of yoga on the quality of life of patients with rheumatic diseases: Systematic review with meta-analysis. Complementary Therapies in Medicine. 2019;46:9-18.

175. Sieczkowska SM, Coimbra DR, Vilarino GT, Andrade A. Effects of resistance training on the health-related quality of life of patients with rheumatic diseases: Systematic review with meta-analysis and meta-regression. Seminars in Arthritis & Rheumatism. 2020;50(2):342-53.

176. Singh JA, Saag KG, Bridges SL, Jr., Akl EA, Bannuru RR, Sullivan MC, et al. 2015 American College of Rheumatology Guideline for the Treatment of Rheumatoid Arthritis. Arthritis & Rheumatology. 2016;68(1):1-26.

177. Singh JA, Guyatt G, Ogdie A, Gladman DD, Deal C, Deodhar A, et al. Special Article: 2018 American College of Rheumatology/National Psoriasis Foundation Guideline for the Treatment of Psoriatic Arthritis. Arthritis & Rheumatology. 2019;71(1):5-32.

178. Smolen JS, Braun J, Dougados M, Emery P, Fitzgerald O, Helliwell P, et al. Treating spondyloarthritis, including ankylosing spondylitis and psoriatic arthritis, to target: recommendations of an international task force. Annals of the Rheumatic Diseases. 2014;73(1):6-16.

179. Straube S, Derry S, Straube C, Moore RA. Vitamin D for the treatment of chronic painful conditions in adults. Cochrane Database of Systematic Reviews. 2015;2015(5):CD007771.

180. Tam LS, Wei JC, Aggarwal A, Baek HJ, Cheung PP, Chiowchanwisawakit P, et al. 2018 APLAR axial spondyloarthritis treatment recommendations. International Journal of Rheumatic Diseases. 2019;22(3):340-56.

181. Tang X, Liu Z, Yang Z, Xu S, Wang M, Chen X, et al. The Effect of Chinese Medicine Compound in the Treatment of Rheumatoid Arthritis on the Level of Rheumatoid Factor and Anti-Cyclic Citrullinated Peptide Antibodies: A Systematic Review and Meta-Analysis. Frontiers in Pharmacology. 2021;12:686360.

182. Tanski W, Swiatoniowska-Lonc N, Tabin M, Jankowska-Polanska B. The Relationship between Fatty Acids and the Development, Course and Treatment of Rheumatoid Arthritis. Nutrients. 2022;14(5):28.

183. Tao W, Zhang Q, Wang L. Investigation of the Clinical Efficacy of Acupuncture Combined with Traditional Chinese Medicine Fumigation in the Treatment of Rheumatoid Arthritis by Meta-Analysis. Contrast Media and Molecular Imaging. 2022;2022 (no pagination):7998725.

184. Theodoridis X, Grammatikopoulou MG, Stamouli EM, Talimtzi P, Pagkalidou E, Zafiriou E, et al. Effectiveness of oral vitamin D supplementation in lessening disease severity among patients with psoriasis: A systematic review and meta-analysis of randomized controlled trials. Nutrition. 2021;82:111024.

185. Tierney M, Fraser A, Kennedy N. Physical activity in rheumatoid arthritis: a systematic review. Journal of Physical Activity & Health. 2012;9(7):1036-48.

186. Trinh K, Zhou F, Belski N, Deng J, Wong CY. The Effect of Acupuncture on Hand and Wrist Pain Intensity, Functional Status, and Quality of Life in Adults: A Systematic Review. Medical Acupuncture. 2022;34(1):34-48.

187. Tsiogkas SG, Grammatikopoulou MG, Gkiouras K, Zafiriou E, Papadopoulos I, Liaskos C, et al. Effect of Crocus sativus (Saffron) Intake on Top of Standard Treatment, on Disease Outcomes and Comorbidities in Patients with Rheumatic Diseases: Synthesis without Meta-Analysis (SWiM) and Level of Adherence to the CONSORT Statement for Randomized Controlled Trials Delivering Herbal Medicine Interventions. Nutrients. 2021;13(12):27.

188. van den Berg R, Baraliakos X, Braun J, van der Heijde D. First update of the current evidence for the management of ankylosing spondylitis with non-pharmacological treatment and non-biologic drugs: a systematic literature review for the ASAS/EULAR management recommendations in ankylosing spondylitis. Rheumatology. 2012;51(8):1388-96.

189. Vedder D, Walrabenstein W, Heslinga M, de Vries R, Nurmohamed M, van Schaardenburg D, et al. Dietary Interventions for Gout and Effect on Cardiovascular Risk Factors: A Systematic Review. Nutrients. 2019;11(12):04.

190. Verhoeven F, Guillot X, Prati C, Mougin F, Tordi N, Demougeot C, et al. Aerobic exercise for axial spondyloarthritis - its effects on disease activity and function as compared to standard physiotherapy: A systematic review and meta-analysis. International Journal of Rheumatic Diseases. 2019;22(2):234-41.

191. Wagenaar CA, van de Put M, Bisschops M, Walrabenstein W, de Jonge CS, Herrema H, et al. The Effect of Dietary Interventions on Chronic Inflammatory Diseases in Relation to the Microbiome: A Systematic Review. Nutrients. 2021;13(9):15.

192. Wang F, Tian ZG, Lv CH, Geng CW. Baguan Therapy Is an Alternative in Treating Ankylosing Spondylitis: A Systematic Review Based on Existing Randomized Controlled Trials. Complementary Medical Research. 2019;26(2):118-25.

193. Wang HR, Fu Q, Liu Z, Li ML, Zhai SQ. A systematic review and meta-analysis of randomized controlled trials: Skin-patch of Chinese herbal medicine for patients with acute gouty arthritis. Journal of Advanced Nursing. 2018;25:25.

194. Wang J, Cui M, Jiao H, Tong Y, Xu J, Zhao Y, et al. Content analysis of systematic reviews on effectiveness of traditional Chinese medicine. Journal of Traditional Chinese Medicine. 2013;33(2):156-63.

195. Wang X, Zu Y, Huang L, Yu J, Zhao H, Wen C, et al. Treatment of rheumatoid arthritis with combination of methotrexate and Tripterygium wilfordii: A meta-analysis. Life Sciences. 2017;171:45-50.

196. Wang Y, Lu S, Wang R, Jiang P, Rao F, Wang B, et al. Integrative effect of yoga practice in patients with knee arthritis: A PRISMA-compliant meta-analysis. Medicine. 2018;97(31):e11742.

197. Wang Y, Zhou Z, Chen L, He X, Li H, Huang Y, et al. Efficacy of Duhuo Jisheng Decoction in Treating Ankylosing Spondylitis: Clinical Evidence and Potential Mechanisms. Evidence-Based Complementary & Alternative Medicine: eCAM. 2022;2022:3305773.

198. Ward L, Stebbings S, Cherkin D, Baxter GD. Yoga for functional ability, pain and psychosocial outcomes in musculoskeletal conditions: a systematic review and meta-analysis. Musculoskeletal Care. 2013;11(4):203-17.

199. Wen Z, Chai Y. Effectiveness of resistance exercises in the treatment of rheumatoid arthritis: A meta-analysis. Medicine. 2021;100(13):e25019.

200. Wendling D, Lukas C, Prati C, Claudepierre P, Gossec L, Goupille P, et al. 2018 update of French Society for Rheumatology (SFR) recommendations about the everyday management of patients with spondyloarthritis. Joint, Bone, Spine: Revue du Rhumatisme. 2018;85(3):275-84.

201. Wong MM, Arcand J, Leung AA, Raj TS, Trieu K, Santos JA, et al. The Science of Salt: A Regularly Updated Systematic Review of Salt and Health Outcomes (August to November 2015). Journal of Clinical Hypertension. 2016;18(10):1054-62.

202. Xiao N, Chen H, He SY, Xue CX, Sui H, Chen J, et al. Evaluating the Efficacy and Adverse Effects of Clearing Heat and Removing Dampness Method of Traditional Chinese Medicine by Comparison with Western Medicine in Patients with Gout. Evidence-Based Complementary & Alternative Medicine: eCAM. 2018;2018:8591349.

203. Xing Q, Fu L, Yu Z, Zhou X. Efficacy and Safety of Integrated Traditional Chinese Medicine and Western Medicine on the Treatment of Rheumatoid Arthritis: A Meta-Analysis. Evidence-Based Complementary and Alternative Medicine. 2020;2020:4348709.

204. Xuan Y, Huang H, Huang Y, Liu D, Hu X, Geng L. The Efficacy and Safety of Simple-Needling Therapy for Treating Ankylosing Spondylitis: A Systematic Review and Meta-Analysis of Randomized Controlled Trials. Evidence-Based Complementary & Alternative Medicine: eCAM. 2020;2020:4276380.

205. Yang J, Li G, Xiong D, Chon TY, Bauer BA. The Impact of Natural Product Dietary Supplements on Patients with Gout: A Systematic Review and Meta-Analysis of Randomized Controlled Trials. Evidence-Based Complementary & Alternative Medicine: eCAM. 2020;2020:7976130.

206. Yang YJ, Deng Y, Liao LL, Peng J, Peng QH, Qin YH. Tripterygium Glycosides Combined with Leflunomide for Rheumatoid Arthritis: A Systematic Review and Meta-Analysis. Evidence-based Complementary and Alternative Medicine. 2020;2020 (no pagination).

207. Yu Y, Wang D, Zhou Q, Wang C, Ma X, Gao Y, et al. Recommendations in clinical practice guidelines on gout: systematic review and consistency analysis. Clinical & Experimental Rheumatology. 2020;38(5):964-72.

208. Yuwen Y, Shi NN, Wang LY, Xie YM, Han XJ, Lu AP. Development of clinical practice guidelines in 11 common diseases with Chinese medicine interventions in China. Chinese Journal of Integrative Medicine. 2012;18(2):112-9.

209. Zao A, Cantista P. The role of land and aquatic exercise in ankylosing spondylitis: a systematic review. Rheumatology International. 2017;37(12):1979-90.

210. Zeng L, Yang T, Yang K, Yu G, Li J, Xiang W, et al. Curcumin and Curcuma longa Extract in the Treatment of 10 Types of Autoimmune Diseases: A Systematic Review and Meta-Analysis of 31 Randomized Controlled Trials. Frontiers in Immunology. 2022;13:896476.

211. Zhang J, Tang R, Zhenyao XIE. Intervention of Traditional Chinese Medicine nursing technique on joint pain in patients with rheumatoid arthritis: A Meta-analysis. Nursing of Integrated Traditional Chinese & Western Medicine. 2020;6(8):20-5.

212. Zhang D, Lyu JT, Zhang B, Zhang XM, Jiang H, Lin ZJ. Comparative efficacy, safety and cost of oral Chinese patent medicines for rheumatoid arthritis: a Bayesian network meta-analysis. BMC Complementary Medicine and Therapies. 2020;20(1):210.

213. Zhao Q, Dong C, Liu Z, Li M, Wang J, Yin Y, et al. The effectiveness of aquatic physical therapy intervention on disease activity and function of ankylosing spondylitis patients: A meta-analysis. Psychology, health & medicine. 2020;25(7):832-43.

214. Zhao S, Duffield SJ, Moots RJ, Goodson NJ. Systematic review of association between vitamin D levels and susceptibility and disease activity of ankylosing spondylitis. Rheumatology. 2014;53(9):1595-603.

215. Zheng W, Zhang J, Shang H. Electro-acupuncture-related adverse events: A systematic review. Medical Acupuncture. 2012;24(2):77-81.

216. Zhou R, Zhu YJ, Chen X, Ma HC, Liu YH, Chang XS, et al. Effect of Sham Acupuncture on Chronic Pain: A Bayesian Network Meta-analysis. Pain Medicine. 2022;22(4):22.

217. Zhou YY, Xia X, Peng WK, Wang QH, Peng JH, Li Y, et al. The effectiveness and safety of Tripterygium wilfordii Hook. F extracts in rheumatoid arthritis: A systematic review and meta-analysis. Frontiers in Pharmacology. 2018;9(APR) (no pagination).

218. Zhu X, Shen X, Hou X, Luo Y, Fu X, Cao M, et al. Total glucosides of paeony for the treatment of rheumatoid arthritis: A methodological and reporting quality evaluation of systematic reviews and meta-analyses. International Immunopharmacology. 2020;88:106920.

219. Zou YY, Zhang HY, Xue L, Ye JJ, Hu GY. Traditional Chinese Eight Brocade Exercise Prescription for Ankylosing Spondylitis: A Quantitative Synthesis. Complementary Medical Research. 2020;27(6):449-53.

220. 冷雨飞, 胡佳琪, 詹刘莉, 胡琳莉, 杜世正, 孙志岭. 抗阻运动在类风湿关节炎病人中应用效果的系统评价. Chinese Nursing Research. 2020;34(17):3041-8.

221. 郭亚丽, 李海婷, 贾伞伞, 文曹丽, 张云. 穴位贴敷疗法治疗类风湿性关节炎疗效的 Meta分析. Chinese Journal of Integrative Nursing. 2021;7(1):50-5.

222. Bjork M, Dragioti E, Alexandersson H, Esbensen BA, Bostrom C, Friden C, et al. Inflammatory Arthritis and the Effect of Physical Activity on Quality of Life and Self-Reported Function: A Systematic Review and Meta-Analysis. Arthritis Care & Research. 2022;74(1):31-43.

223. Byrnes K, Wu PJ, Whillier S. Is Pilates an effective rehabilitation tool? A systematic review. Journal of Bodywork & Movement Therapies. 2018;22(1):192-202.

224. Daily JW, Zhang T, Cao S, Park S. Efficacy and Safety of GuiZhi-ShaoYao-ZhiMu Decoction for Treating Rheumatoid Arthritis: A Systematic Review and Meta-Analysis of Randomized Clinical Trials. Journal of Alternative & Complementary Medicine. 2017;23(10):756-70.

225. Feng C, Chen R, Wang K, Wen C, Xu Z. Chinese traditional medicine (GuiZhi-ShaoYao-ZhiMu decoction) as an add-on medication to methotrexate for rheumatoid arthritis: a meta-analysis of randomized clinical trials. Therapeutic Advances in Chronic Disease. 2021;12:2040622321993438.

226. FitzGerald JD, Dalbeth N, Mikuls T, Brignardello-Petersen R, Guyatt G, Abeles AM, et al. 2020 American College of Rheumatology Guideline for the Management of Gout. Arthritis Care & Research. 2020;72(6):744-60.

227. Geng Q, Liu B, Ma Y, Li H, Shi N, Ouyang G, et al. Effects and Safety of the Tripterygium Glycoside Adjuvant Methotrexate Therapy in Rheumatoid Arthritis: A Systematic Review and Meta-Analysis. Evidence-Based Complementary and Alternative Medicine. 2022;2022 (no pagination):1251478.

228. Gkiouras K, Grammatikopoulou MG, Myrogiannis I, Papamitsou T, Rigopoulou EI, Sakkas LI, et al. Efficacy of n-3 fatty acid supplementation on rheumatoid arthritis' disease activity indicators: a systematic review and meta-analysis of randomized placebo-controlled trials. Critical Reviews in Food Science & Nutrition. 2022:1-15.

229. Guan Y, Hao Y, Guan Y, Bu H, Wang H. The Effect of Vitamin D Supplementation on Rheumatoid Arthritis Patients: A Systematic Review and Meta-Analysis. Frontiers in Medicine. 2020;7:596007.

230. Gwinnutt JM, Wieczorek M, Rodriguez-Carrio J, Balanescu A, Bischoff-Ferrari HA, Boonen A, et al. Effects of diet on the outcomes of rheumatic and musculoskeletal diseases (RMDs): systematic review and meta-analyses informing the 2021 EULAR recommendations for lifestyle improvements in people with RMDs. RMD Open. 2022;8(2):06.

231. Gwinnutt JM, Wieczorek M, Cavalli G, Balanescu A, Bischoff-Ferrari HA, Boonen A, et al. Effects of physical exercise and body weight on disease-specific outcomes of people with rheumatic and musculoskeletal diseases (RMDs): systematic reviews and meta-analyses informing the 2021 EULAR recommendations for lifestyle improvements in people with RMDs. RMD Open. 2022;8(1):03.

232. Hagen KB, Dagfinrud H, Moe RH, Osteras N, Kjeken I, Grotle M, et al. Exercise therapy for bone and muscle health: an overview of systematic reviews. BMC Medicine. 2012;10:167.

233. Han R, Ren HC, Zhou S, Gu S, Gu YY, Sze DM, et al. Conventional disease-modifying anti-rheumatic drugs combined with Chinese Herbal Medicines for rheumatoid arthritis: A systematic review and meta-analysis. Journal of Traditional & Complementary Medicine. 2022;12(5):437-46.

234. Hu H, Xu A, Gao C, Wang Z, Wu X. The effect of physical exercise on rheumatoid arthritis: An overview of systematic reviews and meta-analysis. Journal of Advanced Nursing. 2021;77(2):506-22.

235. Jo H-G, Seo J, Lee D. Clinical evidence construction of East Asian herbal medicine for inflammatory pain in rheumatoid arthritis based on integrative data mining approach. Pharmacological Research. 2022;185:106460.

236. Kou H, Qing Z, Guo H, Zhang R, Ma J. Effect of vitamin E supplementation in rheumatoid arthritis: a systematic review and meta-analysis. European Journal of Clinical Nutrition. 2022;25(2):25.

237. Letarouilly JG, Sanchez P, Nguyen Y, Sigaux J, Czernichow S, Flipo RM, et al. Efficacy of Spice Supplementation in Rheumatoid Arthritis: A Systematic Literature Review. Nutrients. 2020;12(12):11.

238. Li XX, Han M, Wang YY, Liu JP. Chinese herbal medicine for gout: a systematic review of randomized clinical trials. Clinical Rheumatology. 2013;32(7):943-59.

239. Li S, Liu D, Chen Z, Wei S, Xu W, Li X, et al. Comparative efficacy and safety of four classical prescriptions for clearing damp-heat recommended by clinical guidelines in treating rheumatoid arthritis: a network meta-analysis. Annals of Palliative Medicine. 2021;10(7):7298-328.

240. Li H, Man S, Zhang L, Hu L, Song H. Clinical Efficacy of Acupuncture for the Treatment of Rheumatoid Arthritis: Meta-Analysis of Randomized Clinical Trials. Evidence-Based Complementary and Alternative Medicine. 2022;2022 (no pagination):5264977.

241. Li H, Hu R, Xu S, Dai Z, Wu X, Hu J, et al. Tripterygium wilfordii Hook. f. Preparations for Rheumatoid Arthritis: An Overview of Systematic Reviews. Evidence-Based Complementary and Alternative Medicine. 2022;2022:3151936.

242. Liang H, Zhang H, Ji H, Wang C. Effects of home-based exercise intervention on health-related quality of life for patients with ankylosing spondylitis: a meta-analysis. Clinical Rheumatology. 2015;34(10):1737-44.

243. Liang H, Li WR, Zhang H, Tian X, Wei W, Wang CM. Concurrent Intervention With Exercises and Stabilized Tumor Necrosis Factor Inhibitor Therapy Reduced the Disease Activity in Patients With Ankylosing Spondylitis: A Meta-Analysis. Medicine. 2015;94(50):e2254.

244. Liu YF, Zhang Z, Zhang JJ, Chen Z, Tu SH, Xing GL, et al. The Derivative of Tripterygium wilfordii Hook F - Kunxian Capsule, Attenuated Rheumatoid Arthritis: A Systematic Review and Meta-Analysis. Evidence-Based Complementary and Alternative Medicine. 2020;2020 (no pagination):4178140.

245. Luo J, Jin DE, Yang GY, Zhang YZ, Wang JM, Kong WP, et al. Total glucosides of paeony for rheumatoid arthritis: A systematic review of randomized controlled trials. Complementary Therapies in Medicine. 2017;34:46-56.

246. Lu WW, Zhang JM, Lv ZT, Chen AM. Update on the Clinical Effect of Acupuncture Therapy in Patients with Gouty Arthritis: Systematic Review and Meta-Analysis. Evidence-Based Complementary & Alternative Medicine: eCAM. 2016;2016:9451670.

247. Lu HL, Chang CM, Hsieh PC, Wang JC, Kung YY. The effects of acupuncture and related techniques on patients with rheumatoid arthritis: A systematic review and meta-analysis. Journal of the Chinese Medical Association: JCMA. 2022;85(3):388-400.

248. Mudano AS, Tugwell P, Wells GA, Singh JA. Tai Chi for rheumatoid arthritis. Cochrane Database of Systematic Reviews. 2019;9(9):CD004849.

249. Nguyen Y, Sigaux J, Letarouilly JG, Sanchez P, Czernichow S, Flipo RM, et al. Efficacy of Oral Vitamin Supplementation in Inflammatory Rheumatic Disorders: A Systematic Review and Meta-Analysis of Randomized Controlled Trials. Nutrients. 2020;13(1):30.

250. Ortolan A, Felicetti M, Lorenzin M, Cozzi G, Ometto F, Striani G, et al. The impact of diet on disease activity in spondyloarthritis: A systematic literature review. Joint Bone Spine. 2023;90(2):105476.

251. Ortolan A, Webers C, Sepriano A, Falzon L, Baraliakos X, Landewe RB, et al. Efficacy and safety of non-pharmacological and non-biological interventions: a systematic literature review informing the 2022 update of the ASAS/EULAR recommendations for the management of axial spondyloarthritis. Annals of the Rheumatic Diseases. 2023;82(1):142-52.

252. Pecourneau V, Degboe Y, Barnetche T, Cantagrel A, Constantin A, Ruyssen-Witrand A. Effectiveness of Exercise Programs in Ankylosing Spondylitis: A Meta-Analysis of Randomized Controlled Trials. Archives of Physical Medicine & Rehabilitation. 2018;99(2):383-9.e1.

253. Philippou E, Petersson SD, Rodomar C, Nikiphorou E. Rheumatoid arthritis and dietary interventions: systematic review of clinical trials. Nutrition Reviews. 2021;79(4):410-28.

254. Regnaux JP, Davergne T, Palazzo C, Roren A, Rannou F, Boutron I, et al. Exercise programmes for ankylosing spondylitis. Cochrane Database of Systematic Reviews. 2019;10(10):CD011321.

255. Schonenberger KA, Schupfer AC, Gloy VL, Hasler P, Stanga Z, Kaegi-braun N, et al. Effect of anti-inflammatory diets on pain in rheumatoid arthritis: A systematic review and meta-analysis. Nutrients. 2021;13(12) (no pagination)(12).

256. Sieczkowska SM, Smaira FI, Mazzolani BC, Gualano B, Roschel H, Pecanha T. Efficacy of home-based physical activity interventions in patients with autoimmune rheumatic diseases: A systematic review and meta-analysis. Seminars in Arthritis & Rheumatism. 2021;51(3):576-87.

257. Sigaux J, Mathieu S, Nguyen Y, Sanchez P, Letarouilly JG, Soubrier M, et al. Impact of type and dose of oral polyunsaturated fatty acid supplementation on disease activity in inflammatory rheumatic diseases: a systematic literature review and meta-analysis. Arthritis Research & Therapy. 2022;24(1):100.

258. Sobue Y, Kojima T, Ito H, Nishida K, Matsushita I, Kaneko Y, et al. Does exercise therapy improve patient-reported outcomes in rheumatoid arthritis? A systematic review and meta-analysis for the update of the 2020 JCR guidelines for the management of rheumatoid arthritis. Modern Rheumatology. 2022;32(1):96-104.

259. Sun ZL, Xu X, Du SZ, Jiang X. Moxibustion for treating rheumatoid arthritis: A systematic review and meta-analysis of randomized controlled trials. European Journal of Integrative Medicine. 2014;6(6):621-30.

260. Wan R, Fan Y, Zhao A, Xing Y, Huang X, Zhou L, et al. Comparison of Efficacy of Acupuncture-Related Therapy in the Treatment of Rheumatoid Arthritis: A Network Meta-Analysis of Randomized Controlled Trials. Frontiers in Immunology. 2022;13:829409.

261. Wang HL, Jiang Q, Feng XH, Zhang HD, Ge L, Luo CG, et al. Tripterygium wilfordii Hook F versus conventional synthetic disease-modifying anti-rheumatic drugs as monotherapy for rheumatoid arthritis: a systematic review and network meta-analysis. BMC Complementary & Alternative Medicine. 2016;16:215.

262. Wang J, Chen N, Fang L, Feng Z, Li G, Mucelli A, et al. A Systematic Review about the Efficacy and Safety of Tripterygium wilfordii Hook.f. Preparations Used for the Management of Rheumatoid Arthritis. Evidence-Based Complementary & Alternative Medicine: eCAM. 2018;2018:1567463.

263. Wang H, Huang Y, Shen P, Wang Y, Qin K, Huang Y, et al. Modified Si-Miao Pill for Rheumatoid Arthritis: A Systematic Review and Meta-Analysis. Evidence-Based Complementary & Alternative Medicine: eCAM. 2020;2020:7672152.

264. Williams MA, Srikesavan C, Heine PJ, Bruce J, Brosseau L, Hoxey-Thomas N, et al. Exercise for rheumatoid arthritis of the hand. Cochrane Database of Systematic Reviews. 2018;7(7):CD003832.

265. Xu X, Li QJ, Xia S, Wang MM, Ji W. Tripterygium Glycosides for Treating Late-onset Rheumatoid Arthritis: A Systematic Review and Meta-analysis. Alternative Therapies in Health & Medicine. 2016;22(6):32-9.

266. Ye X, Chen Z, Shen Z, Chen G, Xu X. Yoga for Treating Rheumatoid Arthritis: A Systematic Review and Meta-Analysis. Frontiers in Medicine. 2020;7:586665.

267. Ye H, Weng H, Xu Y, Wang L, Wang Q, Xu G. Effectiveness and safety of aerobic exercise for rheumatoid arthritis: a systematic review and meta-analysis of randomized controlled trials. BMC Sports Science, Medicine and Rehabilitation. 2022;14(1):17.

268. Zeng L, Deng Y, He Q, Yang K, Li J, Xiang W, et al. Safety and efficacy of probiotic supplementation in 8 types of inflammatory arthritis: A systematic review and meta-analysis of 34 randomized controlled trials. Frontiers in Immunology. 2022;13:961325.

269. Zeng L, Yang T, Yang K, Yu G, Li J, Xiang W, et al. Efficacy and Safety of Curcumin and Curcuma longa Extract in the Treatment of Arthritis: A Systematic Review and Meta-Analysis of Randomized Controlled Trial. Frontiers in Immunology. 2022;13:891822.

270. Zhang Q, Li R, Liu J, Peng W, Fan W, Gao Y, et al. Efficacy and tolerability of Guizhi-Shaoyao-Zhimu decoction in gout patients: a systematic review and Meta-analysis. Pharmaceutical Biology. 2020;58(1):1023-34.

271. Zheng W, Mei Y, Chen C, Cai L, Chen H. The effectiveness and safety of Tripterygium wilfordii glycosides combined with disease-modifying anti-rheumatic drugs in the treatment of rheumatoid arthritis: A systematic review and meta-analysis of 40 randomized controlled trials. Phytotherapy Research. 2021;35(6):2902-24.

272. Zhou L, Liu L, Liu X, Chen P, Liu L, Zhang Y, et al. Systematic review and meta-analysis of the clinical efficacy and adverse effects of Chinese herbal decoction for the treatment of gout. PLoS ONE [Electronic Resource]. 2014;9(1):e85008.

273. Zhou B, Wang G, Hong Y, Xu S, Wang J, Yu H, et al. Mindfulness interventions for rheumatoid arthritis: A systematic review and meta-analysis. Complementary Therapies in Clinical Practice. 2020;39:101088.

1. Moxibustion refers to the burning of moxa (mugwort leaves) on or near acupuncture points [↑](#endnote-ref-2)
